# Obesity-Related Cancers in Relation to Use of Statins and Testosterone Replacement Therapy Among Older Women: SEER-Medicare 2007–2015

**DOI:** 10.3390/ph18091413

**Published:** 2025-09-19

**Authors:** Maryam R. Hussain, Shannon Wu, Diane Saab, Biai Digbeu, Omer Abdelgadir, Jesus Gibran Hernandez-Perez, Luisa E. Torres-Sanchez, Tammy Leonard, Miguel Cano, Yong-Fang Kuo, Alejandro Villasante-Tezanos, David S. Lopez

**Affiliations:** 1 School of Public and Population Health, University of Texas Medical Branch, Galveston, TX 77555, USA; shawu@utmb.edu (S.W.); disaab@utmb.edu (D.S.); bidigbeu@utmb.edu (B.D.); yokuo@utmb.edu (Y.-F.K.); alvillas@utmb.edu (A.V.-T.); 2 Department of Internal Medicine, University of New Mexico Health Sciences Center, Albuquerque, NM 87131, USA; 3 Graduate School of Biomedical Science, University of Texas Medical Branch, Galveston, TX 77555, USA; omabdelg@utmb.edu; 4 O’Donnell School of Public Health, University of Texas Southwestern Medical Center, Dallas, TX 75390, USA; tammy.leonard@utsouthwestern.edu (T.L.); miguel.cano@utsouthwestern.edu (M.C.); david.lopez3@utsouthwestern.edu (D.S.L.); 5 Center for Population Health Research, National Institute of Public Health (INSP), Cuernavaca 62100, Morelos, Mexico; nut.jgibran@hotmail.com (J.G.H.-P.); ltorress@insp.mx (L.E.T.-S.); 6 Harold Simmons Comprehensive Cancer Center, University of Texas Southwestern Medical Center, Dallas, TX 75235, USA

**Keywords:** statins, testosterone replacement therapy, obesity-related cancer, breast cancer, colorectal cancer, ovarian cancer, endometrial cancer

## Abstract

**Background/Objectives**: The associations of statins and testosterone replacement therapy (TTh) with the risk of obesity-related cancers (ORC, breast [BrCa], colorectal [CRC], ovarian, and endometrial cancers) in older women remain poorly understood. This study examined the associations between the use of statins and TTh with risk of ORC and its cancer-specific sites in women aged 65 years and older. **Methods**: A retrospective cohort study was conducted using 2007–2015 SEER-Medicare data, including 142,772 women aged ≥ 65 years. We identified 52,086 women with incident ORC (BrCa [n = 32,707], CRC [n = 11,070], ovarian [n = 2601], and endometrial [n = 5708] cancers). The primary exposures were use of statins and TTh. Weighted multivariable time-dependent Cox proportional hazards and models were conducted to estimate hazard ratios (HRs) of incident ORC. **Results**: We found an inverse association of statins with incident [HR, 0.76; 95% CI: 0.74, 0.78], high-grade [HR, 0.75; 95% CI: 0.72, 0.78], and advanced-stage [HR, 0.91; 95% CI: 0.88, 0.95] ORC. Concurrent use of statins and TTh was associated with a reduced incidence of ORC and high-grade ORC. Similar associations were observed with BrCa. Statins were inversely associated with high-grade ovarian cancer and endometrial cancer (incident, high-grade, and advanced-stage). **Conclusions**: Use of statins was inversely associated with ORC, BrCa, and endometrial cancer (high-grade and advanced-stage) and high-grade ovarian cancer in older women. Concurrent use of statins and TTh was inversely associated with ORC and BrCa and their high-grade disease. Future prospective studies are needed to substantiate these findings, especially with a focus to examine time– and dose–response associations and to identify underlying biological mechanisms through which statins and TTh influence incidence of ORC.

## 1. Introduction

Obesity is a complex and preventable chronic condition that has been strongly linked to several cancers, including breast cancer (BrCa), ovarian cancer, endometrial cancer, and colorectal cancer (CRC), which are collectively referred to as obesity-related cancers (ORC) [1,2,3]. These malignancies are projected to account for a significant portion of cancer diagnoses in women; for 2025, the American Cancer Society estimates 316,950 new cases of BrCa, 71,810 of CRC, 69,120 of endometrial cancer, and 20,890 of ovarian cancer [4]. While excess body weight does not account for every case, a substantial body of evidence links these malignancies to obesity and its metabolic consequences [3]. In fact, approximately 9.6% of all new cancer cases among women are attributed to obesity, highlighting its profound and growing impact on public health [3].

A growing body of evidence suggests that the use of statins and testosterone replacement therapy (TTh), independently or jointly, may offer potential benefits in reducing the risk of ORC in women [5,6,7,8]. Statins, primarily prescribed for high cholesterol, show promise in cancer prevention due to their anti-cancer properties [9]. While some research suggests potential anti-cancer effects, their influence on ORC remains unclear [10]. Several studies have demonstrated a reduced risk of BrCa, CRC, and ovarian cancer associated with statin use in women [6,8]. However, some meta-analyses have found no significant association between statin use and the risk of CRC, ovarian, or endometrial cancers [11,12,13].

TTh in women is another area of increasing interest but limited research. TTh offers potential benefits for improved brain and bone health, and cardiovascular protection [14]. However, its clinical use in women lacks clear guidelines [15] and access to FDA-approved formulations is hindered [16], leading many women to rely on off-label men’s TTh products or compounded topical and pellet forms [17]. Consequently, there is limited research examining the independent association between TTh and the risk of ORC in women. One retrospective study found a reduced risk of BrCa associated with TTh use in women, both pre- and post-menopausal [14]. Similarly, Dimitrakakis et al. found that TTh combined with conventional hormone therapy was associated with a reduced risk of BrCa among post-menopausal women [18].

Given the increasing burden of ORC, the widespread use of statins, and the critical knowledge gap regarding the effects of TTh and its interaction with statins, our study examines the independent and joint effects of statins and TTh on the risk of ORC, including BrCa, CRC, ovarian, and endometrial cancers, among women aged 65 and older.

## 2. Results

**Figure 1:**
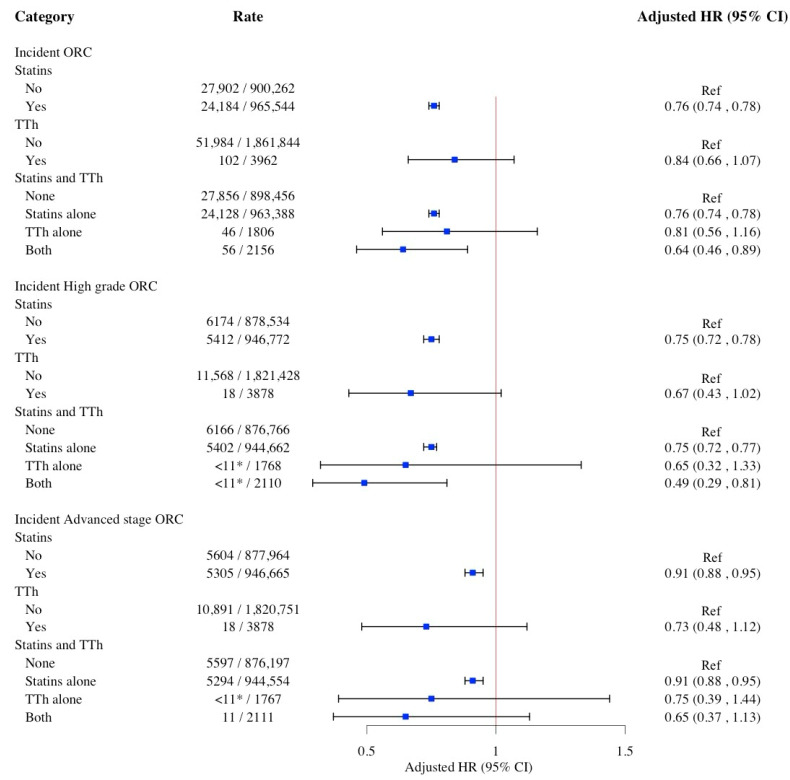
Weighted multivariable Cox proportional hazard models for the association between use of statins and TTh and risk of ORC. Cox models adjusted for age, race/ethnicity, education level, poverty level, umber of primary care physician visits, number of breast cancer screenings, CRC colonoscopy, ovarian cancer screenings, NCI Charlson Comorbidity Index, hyperlipidemia, hypertension, diabetes, use of insulin, muscular wasting and disuse atrophy, malaise and fatigue, osteoporosis, and depressive disorder. Abbreviations: CI, confidence interval; HR, hazard ratio; ORC, obesity-related cancers; TTh, testosterone replacement therapy. * SEER-Medicare data presentation guideline has been followed and all counts less than 11 have been suppressed.

A total of 142,772 women were identified in the overall population. Of these, 52,086 had a primary diagnosis of ORC, which included BrCa (n = 32,707), CRC (n = 1070), ovarian cancer (n = 2601), and endometrial cancer (n = 5708). The remaining 90,686 women were cancer-free between January 2007 and December 2015, as identified in the Surveillance, Epidemiology and End Results (SEER) Medicare database (Table 1 and Figure 1). Approximately 50% of women did not use either statins or TTh, while 49.7% used statins alone. Less than 1% used TTh alone or a combination of both statins and TTh. Compared to women who did not use either medication, those using statins alone, TTh alone, or both were less likely to be White and less likely to have a diagnosis of BrCa. However, they were more likely to be younger and to have comorbidities such as hyperlipidemia, hypertension, muscular wasting, disuse atrophy, malaise, fatigue, osteoporosis, depression, diabetes, cardiovascular disease, a higher Charlson Comorbidity Index (CCI) score, and a greater number of healthcare visits. Furthermore, users of statins and TTh in combination, TTh alone, and statins alone were more likely to report having less than 12 years of education and living below the poverty line. Among these groups, those using TTh alone were the most likely to undergo BrCa screening, CRC screening, and ovarian cancer screening, and were also more likely to have frequent primary care physician (PCP) visits (Table 1).

Figure 1 illustrates the associations between statins and TTh use with the risk of ORC. Compared to women who did not use statins, statins use had an inverse association with incident ORC [Hazard Ratio (HR): 0.76; 95% Confidence Interval (CI): 0.74, 0.78], high-grade ORC (HR: 0.75; 95% CI: 0.72–0.78), and advanced-stage ORC (HR: 0.91; 95% CI: 0.88–0.95). When we compared to women who did not utilize either statins or TTh, the concurrent use of statins and TTh was significantly associated with a reduced risk of ORC (HR: 0.64; 95% CI: 0.46–0.89) and high-grade ORC (HR: 0.49; 95% CI: 0.29–0.81), but not advanced stage of ORC.

**Figure 2:**
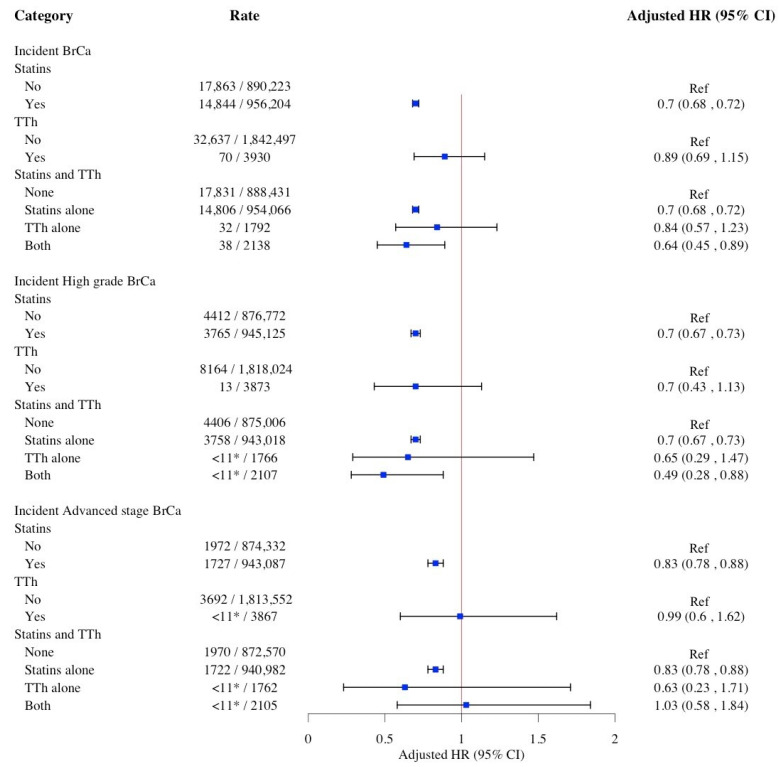
Weighted multivariable Cox proportional hazard models for the association between use of statins and TTh and risk of BrCa. Cox models adjusted for age, race/ethnicity, education level, poverty level, umber of primary care physician visits, number of breast cancer screenings, NCI Charlson Comorbidity Index, hyperlipidemia, hypertension, diabetes, use of insulin, muscular wasting and disuse atrophy, malaise and fatigue, osteoporosis, and depressive disorder. Abbreviations: BrCa, breast cancer; CI, confidence interval; HR, hazard ratio; TTh, testosterone replacement therapy. * SEER-Medicare data presentation guideline has been followed and all counts less than 11 have been suppressed.

Figure 2 presents the associations between use of statins and TTh and risk of BrCa. Statins use was associated with a reduced risk of incident BrCa [HR: 0.70; 95% CI: 0.68, 0.72], high-grade BrCa [HR: 0.70; 95% CI: 0.67, 0.73], and advanced-stage BrCa [HR: 0.83; 95% CI: 0.78, 0.88]. Although the measures of association between TTh and incident BrCa, including advanced-stage and high-grade BrCa, were lower than HR = 0.89 in general, they did not reach statistical significance. In contrast, compared to women who did not use either statins or TTh, the concurrent use of statins and TTh was inversely associated with both incident BrCa [HR: 0.70; 95% CI: 0.68, 0.72] and high-grade BrCa [HR: 0.70; 95% CI: 0.67, 0.73], but not with advanced stage of BrCa.

There was no significant association between statins or TTh use and incident CRC (Table 2), nor were any significant associations observed with the advanced-stage or high-grade CRC. However, statins use, compared to no statins use, was associated with a reduced risk of high-grade ovarian cancer [HR: 0.80; 95% CI: 0.69, 0.93] (Table 3). In the case of endometrial cancer, statins use was associated with a reduced risk of endometrial cancer [HR: 0.67; 95% CI: 0.64, 0.70], including advanced-stage [HR: 0.76; 95% CI: 0.68, 0.84] and high-grade [HR: 0.70; 95% CI: 0.62, 0.78] endometrial cancer (Table 4). Due to the limited sample size, the joint associations of statins and TTh with the stage and grade of CRC, ovarian, and endometrial cancers were not calculated (Table 3 and Table 4).

## 3. Discussion

This population-based cohort study examined the association between statins and TTh use and the risk of ORC, including BrCa, CRC, endometrial cancer, and ovarian cancer, in older women. Overall, we identified independent associations between statins use and ORC, as well as joint associations between use of statins and TTh and ORC. Statins use was inversely associated with overall ORC, BrCa, and endometrial cancer, across incident, high grade, and advanced stages. The concurrent use of statins and TTh was inversely associated with a reduced risk of incident ORC and BrCa, including high-grade ORC. To our knowledge, this is the first study to quantify the joint effects of statins and TTh use on incident ORC, including advanced-stage and high-grade ORC, among women aged 65 years and older.

The observed direction of the association between use of statins and BrCa aligns with findings from previous observational studies [6,19]. However, direct comparisons with prior research are challenging due to our unique methodological approach, which involves evaluating the joint effects of statins and TTh. In contrast, other earlier studies have assessed these factors independently. For example, a study conducted by Eliassen et al. reported no significant impact of statins on BrCa risk [20], despite the well-established role of cholesterol as a precursor in steroidogenesis, and the strong link between sex steroid hormones and risk of BrCa. Similarly, Cauley et al. found no significant association between statins use and BrCa risk, although a potential for risk reduction was observed with hydrophobic statins [21].

Previous studies have investigated the potential association between distinct classes of statins and the risk of different types of ORC [22]. A meta-analysis conducted by Liu et al. revealed that lipophilic statins were associated with a decreased risk of CRC compared to hydrophilic statins [22]. Although we observed a slight reduction in the incidence of CRC, this finding did not reach statistical significance. The relationship between statin use and CRC risk remains highly debated due to significant heterogeneity across studies. This variability stems from differences in study design, including statin type, dosage, duration of use, and the methods employed to assess statin exposure [22]. For instance, aggregating all statins together can mask class-specific effects, particularly regarding cancer risk and tissue penetration, due to differences in the biological mechanisms of lipophilic versus hydrophilic statins. Lipophilic statins (e.g., simvastatin, atorvastatin) have greater cellular and tissue penetration, including into non-hepatic tissues such as the colon, compared to hydrophilic statins (e.g., pravastatin, rosuvastatin), which are more hepatoselective and less likely to accumulate in extrahepatic tissues [23]. This distinction is clinically relevant because lipophilic statins may exert more pronounced pleiotropic effects, including anti-inflammatory and potential antitumor actions, in tissues such as the colon. However, some observational studies have suggested that prolonged use of specific lipophilic statins (simvastatin) may be associated with increased risk of certain cancers, such as lung and bladder cancer, while no significant association was found with CRC [24]. Meta-analyses and large cohort studies have generally found no overall effect of statin type or duration on cancer incidence or mortality, but these analyses may lack the granularity to detect statin-specific or tissue-specific effects [25]. Moreover, differences in study populations, such as varying baseline CRC risk, age, comorbidities, and lifestyle factors, can significantly influence outcomes. The duration and dose of statin use are also crucial, with some studies indicating that longer-term and higher-dose use may be more effective in modulating CRC risk [8,26,27,28].

Irvin et al. observed a lower risk of ovarian cancer associated with statins use, with the observed effects varying based on statins class, duration of use, and cancer histology [7]. Our findings align with those of Irvin et al., as we also identified a decreased risk of high-grade ovarian cancer associated with statins use. It is important to acknowledge that in contrast to the studies by Liu et al., and Irvin et al., our analysis did not differentiate between distinct statins classes; thus, our estimated association between statins use and cancers may be downward biased if the association only exists among a subset of statins classes. Additionally, our study demonstrated a significant reduction in the risk of endometrial cancer associated with statins use, a finding observed across incident, high-grade, and advanced-stage endometrial cancer. This finding aligns with the observations of Lavie et al., who reported a similar decreased risk among women using statins for more than one year prior to diagnosis [29]. However, we did not identify other studies specifically investigating this association. This paucity of research underscores the need for further investigation to support our findings.

Existing research has demonstrated that different cancer cell types exhibit varying degrees of sensitivity to statins therapy, with specific cellular markers potentially predicting treatment response [30]. This observed variability may offer a plausible explanation for the lack of significant findings for certain cancers in our study. Furthermore, mechanistic studies have revealed the multifaceted effects of statins on cancer progression, including inhibition of cell proliferation, the induction of apoptosis, and the disruption of metastatic mechanisms [31]. These findings provide a reasonable biological rationale for the anti-cancer properties of statins.

The absence of FDA-approved testosterone products for women presents a significant limitation in research examining the independent effects of TTh on ORC [14]. Therefore, the findings of this study could be interpreted within the broader context of research investigating the relationship between endogenous testosterone levels and ORC risk. While our study did not find significant associations between TTh and incident BrCa, previous research has yielded mixed results. For example, Donovitz et al. observed a notable reduction in BrCa risk with exogenous testosterone, either administered alone or in combination with estrogen pellets, in both pre- and post-menopausal women [14]. In contrast, Tamimi et al. found that exogenous testosterone was associated with an increased risk of BrCa [32]. This finding aligns with studies examining endogenous testosterone levels in pre- and post-menopausal women, which have also reported an increased risk of BrCa [32,33]. Furthermore, our findings are consistent with studies indicating that the circulating testosterone level was not associated with incident CRC in post-menopausal women [34]. Conversely, a Japanese study involving 185 CRC patients and 361 controls, reported a positive association between endogenous testosterone and CRC risk in post-menopausal women [35].

Although our study found a significant association between TTh and a reduced risk of high-grade endometrial cancer, the reliability of this result is constrained by the relatively small sample size. In particular, the number of TTh users represented less than 1% of the study population, which substantially limits the statistical power and the precision of the effect estimates. This limitation is especially relevant when interpreting associations in high-grade cancer subgroups, where the low exposure prevalence increases the risk of Type II error. In contrast, the European Prospective Investigation into Cancer and Nutrition (EPIC) study reported a positive association between higher levels of circulating testosterone and endometrial cancer risk. However, due to a small sample size, they were unable to draw definitive conclusions regarding pre-menopausal women [36]. These mixed findings underscore the need for larger, adequately powered studies to assess the role of TTh and endogenous testosterone in endometrial cancer risk.

In our study, we identified significant associations between statins use and TTh, as well as their joint effects, with reduced risks of incident and high-grade ORC and BrCa. To the best of our knowledge, no prior research has examined the concurrent use of statins and TTh in relation to ORC in women, making our findings novel and challenging to directly compare with existing literature. Nonetheless, there is biological plausibility for an interaction between cholesterol and testosterone, particularly considering that cholesterol serves as a key precursor in steroidogenesis [37]. The potential synergistic effects of statin therapy and TTh are not well-established, with most of the limited medical literature focusing on cardiometabolic outcomes rather than direct cancer-related effects. Although some studies suggest statins may modestly lower total testosterone levels, these changes are generally not clinically significant and do not typically lead to hypogonadism or adverse effects on sexual function [38]. In a small number of studies, the joint use of statins and TTh has shown potential for additive or synergistic cardiometabolic benefits. For instance, in men with late-onset hypogonadism who were already on statin therapy, the addition of TTh was shown to improve insulin sensitivity and reduce key markers of inflammation and cardiovascular risk, including Low-Density Lipoprotein (LDL) cholesterol, High-sensitivity C-reactive protein (hs-CRP), and fibrinogen [39]. These findings suggest a plausible synergistic mechanism related to metabolic and inflammatory pathways, which are critical in both cardiovascular disease and certain cancers. However, these results are based on limited data and require confirmation through larger, prospective studies to draw definitive conclusions.

Although there is currently no FDA-approved indication for TTh use in women, off-label prescriptions of TTh, typically approved for men, are often prescribed to women experiencing androgen insufficiency [40]. A natural decline in androgen levels occurs as women age, leading to a range of physical and physiological changes that can significantly affect quality of life [41]. In a review, Mokarram et al. discussed a potential link between testosterone metabolism and the mevalonate pathway, which is crucial for cholesterol production [42]. However, it remains unclear whether both epidemiological and biological evidence support an interaction between low testosterone or testosterone deficiency and hypercholesterolemia, and whether the concurrent treatment of these conditions with TTh and statins could offer therapeutic benefits.

The potential mechanisms linking statins and TTh to ORC risk in women are multifactorial. Statins inhibit 3-hydroxy-3-methylglutaryl coenzyme (HMG-CoA) reductase, disrupting the mevalonate pathway, which reduces isoprenoid synthesis necessary for Ras and Rho GTPase function. This leads to decreased cell proliferation, angiogenesis, and increased apoptosis in cancer cells, along with anti-inflammatory and immunomodulatory effects. Statins also lower dehydroepiandrosterone (DHEA) and sex hormone-binding globulin (SHBG), influencing the hormonal environment and potentially reducing cancer risk, particularly in women with polycystic ovary syndrome (PCOS) [43,44,45,46]. In contrast, TTh, although less studied, can increase estrogen exposure through the aromatization of androgens to estrogens in adipose tissue. In women with obesity, this raises local and systemic estrogen levels, potentially increasing the risk of estrogen-dependent cancers like breast and endometrial cancer. The Endocrine Society notes that this conversion can promote endometrial hyperplasia or polyp formation in women on combined estrogen and TTh, with evidence suggesting exogenous androgens may also directly promote cancer development [47,48,49].

The obesity paradox, wherein individuals with obesity may demonstrate better survival outcomes compared to those with normal or underweight status in certain cancer types, has been reported in the literature, including among patients with breast and colorectal cancer [50,51,52]. In the context of cancer, the link between the obesity paradox and risk of mortality remains controversial due to factors such as unintentional weight loss and cancer-associated cachexia, which are further complicated by the timing and rate of weight loss. However, this phenomenon pertains primarily to cancer prognosis and survival following diagnosis. As our study examines cancer incidence rather than post-diagnosis outcomes, the direct applicability of the obesity paradox to our findings is limited. Nonetheless, we recognize this framework as part of the broader, multifaceted relationship between obesity, weight modulation, and cancer biology.

Obesity often coexists with type 2 diabetes (T2DM) and cardiovascular disease (CVD), leading to the combined use of diabetes medications, statins, and TTh. The side effect profile of this combination suggests an additive, rather than synergistic, risk. While statins may modestly increase HbA1c by less than 0.2%, a change that is not clinically significant and is outweighed by their cardiovascular benefits [25,38], TTh has shown no meaningful improvement in glycemic control, as demonstrated in the TRAVERSE trial [53]. There is no evidence to suggest that combining diabetes medications, statins, and testosterone therapy results in unique or synergistic cancer risks beyond the additive effects of each agent. Given the lack of evidence for synergistic adverse effects, clinical decisions should be based on the established, independent indications for each medication. Patients should be monitored for the known adverse effects of each drug, but no unique surveillance strategies are required for the combination. This reinforces that the benefit-risk calculation strongly supports continuing statin therapy for cardiovascular risk reduction [38,54,55].

Our study has strengths, including a large cohort of women diagnosed with ORC and age-matched cancer-free group, enhancing the robustness and generalizability of our findings. The substantial sample size strengthens the reliability of the results. We utilized detailed, clinically relevant data from Medicare files. A key strength of our design was the effective management of immortal time bias [50]. To ensure accurate alignment of the exposure, both the exposed and unexposed groups had to be enrolled in Medicare Parts A and B for a minimum of six months before the exposure date. This criterion was set to confirm that the exposure occurred at least six months prior to any ORC diagnosis. Nonetheless, several limitations must be noted. The potential for residual confounding persists, and the reliance on Medicare data introduces challenges such as possible coding inaccuracies and gaps in clinical information. Although Medicare Part D prescription data became available in 2007 [56], some individuals categorized as non-users may have received statins or TTh prior to that year. Additionally, our analysis did not capture other concurrent medications or treatments, which may have varied between groups and could influence outcomes. Additionally, the absence of detailed information on medication dosage and duration precluded the evaluation of time– and dose–response relationships. We also did not differentiate between lipophilic and hydrophilic statins, which may exhibit distinct biological effects, such as differences in tissue penetration, anti-inflammatory properties, and modulation of cancer-related signaling pathways, potentially masking subclass-specific associations with ORC risk. Although our study focused on four ORC in women (BrCa, CRC, endometrial, and ovarian), we recognize that excess body fatness is causally linked to additional cancer types, as identified by the International Agency for Research on Cancer (IARC) [57]. Statins are commonly prescribed for hypercholesterolemia management and CVD prevention [55], which could positively impact the health of our study population. While the concurrent use of statins and TTh showed potential beneficial effects, the non-user group might include women with unrecorded cholesterol levels or prior treatment histories, potentially introducing confounding. Additionally, the SEER-Medicare database lacks key information such as laboratory test results, occupational history, environmental exposures, and lifestyle factors (e.g., smoking status, alcohol consumption, diet quality, and physical activity), all of which are important determinants of cancer risk. The absence of these variables may lead to residual confounding, as we were unable to directly account for their influence in our analyses. This limitation is particularly relevant in studies of ORC, where lifestyle and behavioral factors often overlap with clinical risk profiles. Another limitation of this study is the lack of reliable BMI data (≥30 kg/m^2^), a standard measure of obesity. Although the SEER-Medicare database includes a body mass index (BMI) variable, its reporting is often incomplete and inconsistently coded, reducing its reliability. Although not explicitly discouraged, SEER-Medicare flags this variable due to concerns about misclassification and biased adjustment [52]. Given these limitations, we chose not to rely on BMI but instead adjusted for well-established clinical indicators of obesity, such as diabetes, hypertension, hyperlipidemia, insulin use, and the Charlson Comorbidity Index (CCI). These factors likely capture a significant portion of obesity-related risk. However, we recognize the potential for residual confounding from unmeasured or inaccurately measured variables. Lastly, since our study focuses on older women with Medicare coverage, the findings may not be generalizable to men, younger women, those with different insurance statuses, or the uninsured. These limitations highlight the importance of interpreting the results cautiously and considering them within the context of these specific conditions.

## 4. Materials and Methods

### 4.1. Data

This study used data from SEER-Medicare database, which links cancer registry information from 19 SEER regions covering about 30% of the U.S. population with Medicare claims data [56]. The 5% non-cancer Medicare sample was used to represent cancer-free individuals. SEER tracks clinical and demographic data on patients 65 and older. These records are linked to Medicare for long-term follow-up. Our study was approved by the Institutional Review Board (IRB) at the University of Texas Medical Branch (IRB approval no: 20-0237). Because we used de-identified data, informed consent was not required.

### 4.2. Study Cohort

**Figure 3:**
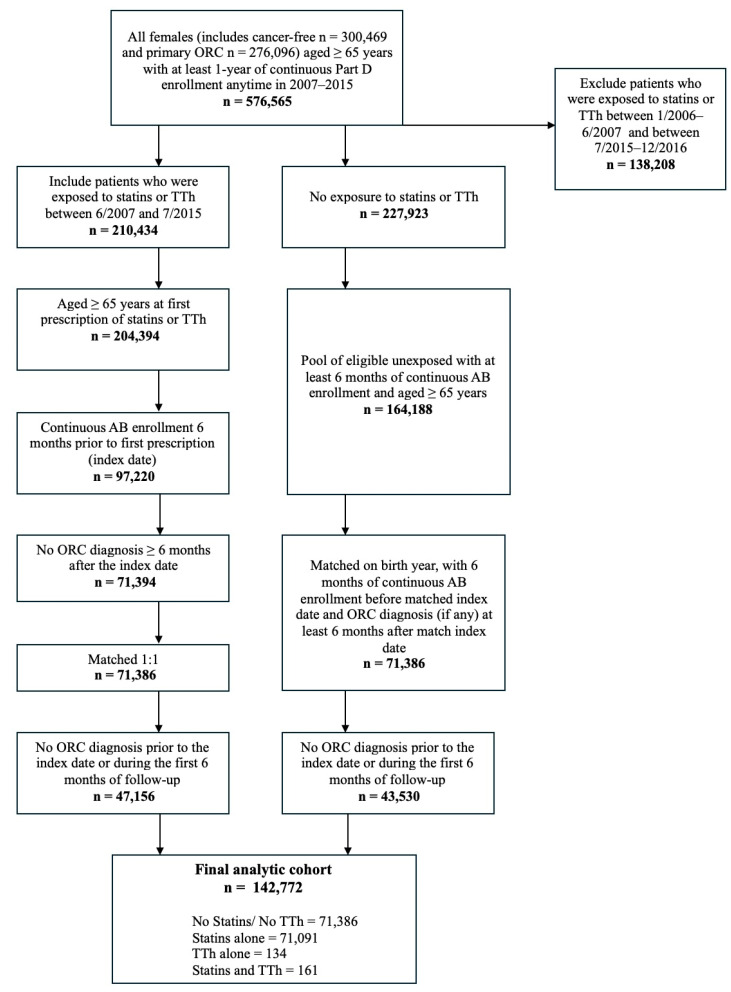
Study inclusion and exclusion criteria.

Figure 3 illustrates the cohort selection process for this retrospective cohort study. The initial cohort included 576,565 women (cancer-free n = 300,469, ORC n = 276,096) aged 65 years or older who had at least one year of continuous enrollment in Medicare Part D between 2007 and 2015. After applying exclusion criteria (Figure 3), the final analytic cohort included 142,772 women. Participants were categorized into two cohorts: an exposed group, comprising individuals who had filled at least one prescription for either statins or TTh between July 2007 and June 2015, and an unexposed group, consisting of individuals with no prescriptions for either medication during that timeframe. To be included in the exposed group, individuals had to be 65 years or older at the time of their first prescription (defined as the index date), with at least six months of continuous enrollment in Medicare Parts A and B prior to this date. Those who did not meet these criteria or whose index date fell within six months of ORC diagnosis were excluded. For the unexposed group, eligible individuals had to be at least 65 years old and have had at least six months of Medicare Part A and B enrollment during the study window. These individuals were then matched 1:1 to exposed participants based on year of birth and were assigned the same index date as their matched exposed counterpart. To ensure baseline comparability, unexposed individuals were required to have a minimum of six months of continuous Medicare Parts A, B, and D coverage prior to the index date, with no ORC diagnosis occurring within six months afterward. When several unexposed individuals were eligible to match a single exposed individual, one was randomly chosen for inclusion.

### 4.3. Primary Exposure: Use of Statins and TTh

The primary exposure was defined as the use of statins and/or TTh prior to the diagnosis of ORC (if any). Prescription information was extracted from Medicare Part D data, with statins and TTh use identified through National Drug Codes (NDC) and Current Procedural Terminology (CPT) codes (available upon request). Exposures were categorized as statins use (Yes/No) and TTh use (Yes/No). Additionally, participants were grouped into four categories: no statins or TTh (reference group), statins only, TTh only, and both statins and TTh. This classification was based on the growing prevalence of statins and TTh use among older women, coupled with emerging evidence suggesting potential synergistic effects between the two treatments [58,59]. The index date was defined as the first prescription date within the study period. For those who used both statins and TTh, we required a minimum of six months between the later of the two prescription dates and the ORC diagnosis to minimize immortal time bias and confounding by indication.

### 4.4. Primary Outcome: Incident ORC

The study outcomes included the initial diagnosis of composite ORC, BrCa, CRC, endometrial cancer, ovarian cancer, and their high-grade and advanced-stage disease. Cancer diagnoses were based on relevant site and histological classifications. Advanced-stage cancers were defined as those classified as stage III or IV according to the American Joint Committee on Cancer (AJCC) staging system, while high-grade cancers were characterized by undifferentiated or poorly differentiated tumor cells. These cancers were selected due to strong evidence linking excess adiposity to their development, a connection well-documented by the IARC [57].

### 4.5. Study Covariates

**Figure 4:**
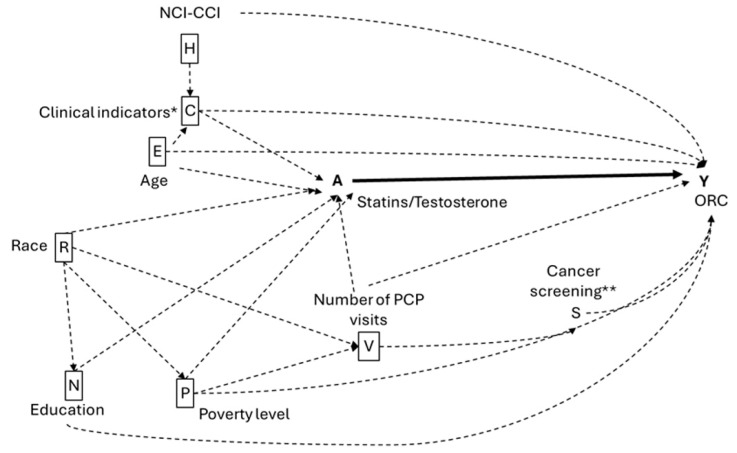
Hypothetical directed acyclic graph (DAG) for the association between statins, TTh and ORC. Solid lines indicate causal pathways; dashed lines indicate non-causal pathways and boxed variables indicate the minimum adjustment set. * Clinical indicators include hyperlipidemia, hypertension, muscular wasting and disuse atrophy, osteoporosis, and depressive disorder. ** Number of BrCa screenings, CRC colonoscopy, ovarian cancer screenings.

**Figure 5:**
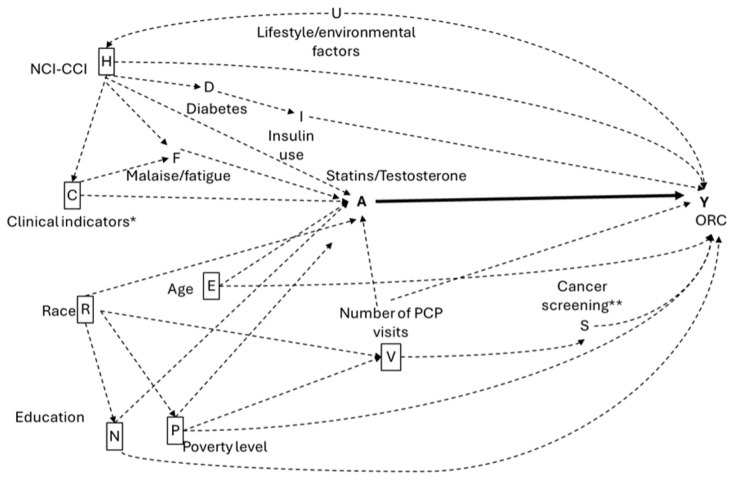
Hypothetical directed acyclic graph (DAG) for the association between statins, TTh and ORC. Solid lines indicate causal pathways; dashed lines indicate non-causal pathways and boxed variables indicate the minimum adjustment set. * Clinical indicators include hyperlipidemia, hypertension, diabetes, use of insulin, muscular wasting and disuse atrophy, malaise and fatigue, osteoporosis, and depressive disorder. ** Number of BrCa screenings, CRC colonoscopy, ovarian cancer screenings.

The study covariates were selected based on a priori knowledge [60], as depicted in the directed acyclic graphs (DAGs) shown in Figure 4 and Figure 5. Race/ethnicity was categorized as White, Black, Hispanic, or Other. The National Cancer Institute (NCI)-CCI was measured from six months prior to the index date, was important for adjusting for baseline comorbidity differences between groups and addressing concerns that comorbidities might influence statins prescriptions (confounding by indication). To avoid ascertainment bias, all covariates were ascertained during the six-month period preceding the index date, which also ensured that the six-month gap between the first medication prescription and ORC diagnosis was consistent. This approach minimized the risk of increased ORC detection in those being worked up for a prescription compared to the reference group of non-prescribed individuals. Clinical indicators identified from Medicare claims using NDC and CPT codes included hyperlipidemia, hypertension, diabetes, insulin use, muscular wasting and disuse atrophy, malaise and fatigue, CVD, osteoporosis, depressive disorder, and anterior pituitary disorders. We measured healthcare utilization through the number of PCP visits, BrCa screenings (having at least one of the following procedures, CPT codes: G0202, G0204, G0206, 76092, 76090, 76091), CRC screening (having at least one of the following procedures, CPT codes: 45378, 45379, 45380, 45381, 45382, 45388, 45384, 45385, 45386, 45389, 45391, 45392, 45390, 45393, 45398, G0105, G0121), and ovarian cancer screening (having at least one of the following procedures, CPT codes: 76830, 76856, 76857), categorizing these variables based on the total number of associated claims from the six months prior to the index date. To account for socioeconomic status (SES), we included US Census tract data that measured the percentage of individuals older than 25 years with less than 12 years of education and the percentage of adults living below the poverty line in each census tract.

### 4.6. Statistical Analysis

Patient characteristics, clinical indicators, SES variables, and medical resource utilization were compared across drug groups (No TTh plus No statins, statins alone, TTh alone, and TTh plus statins) using Chi-square tests for categorical variables and F-tests for continuous variables. The cancer-free group was sampled from the Medicare population, while cancer patients were drawn from the SEER registry. To account for this sampling difference, we applied weights to extrapolate findings to the full population of women aged 65 years and older. This adjustment allowed for accurate estimates of incident ORC, including high-grade and advanced-stage cases, and facilitated the use of weighted multivariable-adjusted Cox proportional hazards models. These models incorporated a priori knowledge to identify potential confounders, as outlined in the covariates section. The multivariable Cox models estimated the risk of ORC among the statins and TTh groups. Time to event (incident ORC) was measured in months from the first prescription date (index date) to either the date of the first ORC diagnosis or the last follow-up date, whichever came first. Patients who were cancer-free at the end of the follow-up period (31 December 2015), those who died before the study concluded, or those lost to follow-up were right-censored. To assess the proportional hazards assumption, we used scaled Schoenfeld residuals. The *p*-values < 0.05 were considered statistically significant. All analyses were conducted using SAS version 9.4 (SAS Institute, Cary, NC, USA). The full analysis code is available in the Supplementary Materials.

## 5. Conclusions

In summary, our study identified a significant inverse association between concurrent use of statins and TTh and the incidence of ORC, including BrCa, among women aged ≥65 years. Statins use alone was independently associated with reduced risk of overall ORC, as well as high-grade and advanced-stage disease. A similar association was observed for BrCa, with statins use linked to lower risk across incident, high-grade, and advanced-stage cases. While these findings provide preliminary evidence that statins and TTh may jointly confer protective effects against ORC and BrCa in older women, the results related to TTh should be interpreted with caution due to the small number of TTh users in the cohort. As with all retrospective analyses, residual confounding and bias cannot be ruled out. Prospective studies are needed to substantiate these associations, explore time– and dose–response relationships, and identify underlying biological mechanisms through which statins and TTh influence the development of ORC.

## Figures and Tables

**Table 1:** Baseline characteristics of study cohort by to the use of statins and TTh in 2007–2015 SEER-Medicare data (n = 142,772).

Characteristics	No. (%)				*p*-Value
	No Statins/No TTh (n = 71,386)	Statins Alone (n = 71,091)	TTh Alone (n = 134)	Statins and TTh (n = 161)	
ORC					
Breast cancer	17,831 (24.98)	14,806 (20.83)	>13 (>9.70) **	>23 (>14.29) **	0.0001 *
Colorectal cancer	5705 (7.99)	5351(7.53)	<11 (<8.21) **	<11 (<6.83) **	0.0019 *
Ovarian	1276 (1.79)	1318 (1.85)	<11 (<8.21) **	<11 (<6.83) **	0.2707
Endometrial	3044 (4.26)	2653 (3.73)	<11 (<8.21) **	<11 (<6.83) **	0.0001 *
ORC Stage ^a^					
Localized	22,259 (31.18)	18,834 (26.49)	39 (29.1)	45 (27.95)	0.0001 *
Advanced	5597 (7.84)	5294 (7.45)	<11 (<8.21) **	11 (6.83)	
Unknown/NA	43,530 (60.98)	46,963 (66.06)	>84 (>62.69) **	105 (65.22)	
ORC Grade ^b^					
Low	21,690 (30.38)	18,726 (26.34)	38 (28.36)	46 (28.57)	0.0001 *
High	6166 (8.64)	5402 (7.6)	<11 (<8.21) **	<11 (<6.83) **	
Unknown/NA	43,530 (60.98)	46,963 (66.06)	>85 (>63.43) **	>104 (>64.60) **	
Age					
65–70	6401 (8.97)	22,977 (32.32)	50 (37.31)	71 (44.1)	0.0001 *
70–75	18,268 (25.59)	17,183 (24.17)	29 (21.64)	42 (26.09)	
75–80	16,453 (23.05)	12,881 (18.12)	24 (17.91)	23 (14.29)	
≥80	30,264 (42.39)	18,050 (25.39)	31 (23.13)	25 (15.53)	
Race/ethnicity					
White	58,929 (82.55)	56,069 (78.87)	>101 (>75.37) **	>122 (>75.78) **	<0.0001 *
Black	5748 (8.05)	7493 (10.54)	<11 (<8.21) **	13 (8.07)	
Hispanic	1708 (2.39)	2296 (3.23)	<11 (<8.21) **	<11 (<6.83) **	
Other	5001 (7.01)	5233 (7.36)	<11 (<8.21) **	15 (9.32)	
Hyperlipidemia	14,889 (20.86)	44,860 (63.1)	41 (30.6)	99 (61.49)	0.0001 *
Hypertension	32,339 (45.3)	53,304 (74.98)	90 (67.16)	128 (79.5)	0.0001 *
Muscular wasting and atrophy	580 (0.81)	799 (1.12)	<11 (<8.21) **	<11 (<6.83) **	0.0001 *
Malaise and fatigue	9799 (13.73)	14,752 (20.75)	50 (37.31)	59 (36.65)	0.0001 *
Osteoporosis	9155 (12.82)	10,635 (14.96)	35 (26.12)	29 (18.01)	0.0001 *
Anterior pituitary disorder	50 (0.07)	55 (0.08)	<11 (<8.21) **	<11 (<6.83) **	0.9238
Depression disorder	3603 (5.05)	5624 (7.91)	12 (8.96)	12 (7.45)	0.0001 *
Diabetes	8607 (12.06)	23,294 (32.77)	29 (21.64)	38 (23.6)	0.0001 *
Cardiovascular disease	28,784 (40.32)	41,621 (58.55)	78 (58.21)	105 (65.22)	0.0001 *
Use of insulin	1038 (1.45)	3998 (5.62)	<11 (<8.21) **	<11 (<6.83) **	0.0001 *
Charlson comorbidity					
None	50,321 (70.49)	36,752 (51.7)	58 (43.28)	83 (51.55)	0.0001 *
One	14081 (19.73)	17,837 (25.09)	39 (29.1)	49 (30.43)	
Two	4500 (6.3)	8758 (12.32)	19 (14.18)	16 (9.94)	
Three or more	2484 (3.48)	7744 (10.89)	18 (13.43)	13 (8.07)	
Number of BrCa screening ^c^, mean (SD)	0.18 (0.43)	0.21 (0.44)	0.25 (0.47)	0.21 (0.42)	<0.0001 *
Number of CRC screening ^d^, mean (SD)	0.04 (0.21)	0.05 (0.23)	0.06 (0.24)	0.05 (0.22)	<0.0001 *
Number of ovarian cancer screening ^e^, mean (SD)	0.02 (0.15)	0.03 (0.17)	0.06 (0.27)	0.06 (0.40)	<0.0001 *
Number of PCP visits, mean (SD)	8.34 (8.99)	11.96 (10.82)	17.25 (12.65)	16.28 (12.09)	<0.0001 *
Percent of adults with <12 years education, mean (SD)	18.37 (11.81)	20.16 (12.44)	18.50 (11.13)	22.36 (13.56)	<0.0001 *
Percent of adults below poverty, mean (SD)	11.26 (8.16)	11.96 (8.68)	12.93 (8.65)	14.87 (9.67)	<0.0001 *

Abbreviation: BrCa, breast cancer; CRC, colorectal cancer; ORC, obesity-related cancer; PCP, primary care provider; SD, standard deviation; TTh, testosterone replacement therapy. ^a^ Advanced-stage ORC indicates AJCC stage III and IV definition, while localized stage ORC indicates stage I and stage II. ^b^ High-grade ORC indicates G3 (poorly differentiated) and G4 (undifferentiated). Low grade ORC indicates G1 (well differentiated) and G2 (moderately differentiated). ^c^ Defined by the following HCPCS/CPT codes: G0202, G0204, G0206, 76092, 76090, 76091. ^d^ Defined by the following HCPCS/CPT codes: G0105, G0121, 45380, 45384, 45385. ^e^ Defined by the following HCPCS/CPT codes: 76830, 76856, 76857. * Statistical significance at the *p* value < 0.05 level. ** SEER-Medicare data presentation guideline has been followed and all counts less than 11 have been suppressed.

**Table 2:** Weighted multivariable Cox proportional hazard models for the association between use of statins and TTh and risk of CRC.

	Incident CRC		High-Grade CRC		Advanced-Stage CRC	
	Rate	HR (95% CI)	Rate	HR (95% CI)	Rate	HR (95% CI)
**Statins**						
No	5710/878,070	Ref	864/873,224	Ref	2142/874,502	Ref
Yes	5360/946,720	0.98 (0.94, 1.02)	813/942,173	1.0 (0.92, 1.09)	2086/943,446	1.03 (0.97, 1.09)
**TTh**						
No	11,056/1,820,916	Ref	676/1,811,536	Ref	4225/18,144,085	Ref
Yes	14/3874	0.67 (0.43, 1.03)	<11/3861 *	0.33 (0.04, 2.37)	<11/3863 *	0.38 (0.12, 1.18)
**Statins and TTh**						
None	5705/876,305	Ref	864/871,464	Ref	2141/872,741	Ref
Statins Alone	5351/944,611	0.98 (0.94, 1.02)	812/940,072	Not Calculated	2084/941,344	1.03 (0.97, 1.09)
TTh Alone	<11/1765 *	0.57 (0.28, 1.14)	<11/1760 *	Not Calculated	<11/1761 *	0.32 (0.04, 2.26)
Both	<11/2109 *	0.72 (0.42, 1.26)	<11/2101 *	Not Calculated	<11/2102 *	0.43 (0.11, 1.75)

Cox models adjusted for age, race/ethnicity, education level, poverty level, umber of primary care physician visits, number of CRC screening, NCI Charlson Comorbidity Index, hyperlipidemia, hypertension, diabetes, use of insulin, muscular wasting and disuse atrophy, malaise and fatigue, osteoporosis, and depressive disorder. Abbreviations: CI, confidence interval; CRC; colorectal cancer; HR, hazard ratio; TTh, testosterone replacement therapy. * SEER-Medicare data presentation guideline has been followed and all counts less than 11 have been suppressed.

**Table 3:** Weighted multivariable Cox proportional hazard models for the association between use of statins and TTh and risk of ovarian cancer.

	Incident Ovarian Cancer		High-Grade Ovarian Cancer		Advanced-Stage Ovarian Cancer	
	Rate	HR (95% CI)	Rate	HR (95% CI)	Rate	HR (95% CI)
**Statins**						
No	1281/873,641	Ref	308/872,668	Ref	855/873,215	Ref
Yes	1320/942,680	0.96 (0.89, 1.04)	295/941,655	0.80 (0.69, 0.93)	891/942,251	0.94 (0.85, 1.03)
**TTh**						
No	2594/1,8124,54	Ref	600/1,810,460	Ref	1740/1,811,600	Ref
Yes	<11/3867 *	0.84 (0.38, 1.87)	<11/3863 *	1.29 (0.31, 5.27)	<11/3866 *	1.0 (0.42, 2.37)
**Statins and TTh**						
None	1276/871,876	Ref	306/870,906	Ref	851/87,1451	Ref
Statins Alone	1318/940,578	Not Calculated	294/939,554	Not Calculated	889/940,149	Not Calculated
TTh Alone	<11/1765 *	Not Calculated	<11/1762 *	Not Calculated	<11/1764 *	Not Calculated
Both	<11/2102 *	Not Calculated	<11/2101 *	Not Calculated	<11/2102 *	Not Calculated

Cox models adjusted for age, race/ethnicity, education level, poverty level, umber of primary care physician visits, number of ovarian cancer screening, NCI Charlson Comorbidity Index, hyperlipidemia, hypertension, diabetes, use of insulin, muscular wasting and disuse atrophy, malaise and fatigue, osteoporosis, and depressive disorder. Abbreviations: CI, confidence interval; HR, hazard ratio; TTh, testosterone replacement therapy. * SEER-Medicare data presentation guideline has been followed and all counts less than 11 have been suppressed.

**Table 4:** Weighted multivariable Cox proportional hazard models for the association between use of statins and TTh and risk of endometrial cancer.

	Incident Endometrial Cancer		High-Grade Endometrial Cancer		Advanced-Stage Endometrial Cancer	
	Rate	HR (95% CI)	Rate	HR (95% CI)	Rate	HR (95% CI)
**Statins**						
No	3048/875,408	Ref	590/872,950	Ref	635/872,995	Ref
Yes	2660/944,020	0.67 (0.64, 0.70)	539/941,899	0.70 (0.62, 0.78)	601/941,961	0.76 (0.68, 0.84)
**TTh**						
No	5697/1,815,557	Ref	1128/1,810,988	Ref	1234/1,811,094	Ref
Yes	11/3871	0.79 (0.47, 1.35)	<11/3861 *	0.42 (0.37, 0.48)	<11/3861 *	0.74 (0.27, 1.98)
**Statins and TTh**						
None	3044/873,644	Ref	590/871,190	Ref	635/871,235	Ref
Statins Alone	2653/941,913	0.67 (0.63, 0.70)	538/939,798	Not Calculated	599/939,859	Not Calculated
TTh Alone	<11/1764 *	0.48 (0.187, 1.22)	<11/1760 *	Not Calculated	<11/1760 *	Not Calculated
Both	<11/2107 *	0.70 (0.37, 1.33)	<11/2101 *	Not Calculated	<11/2102 *	Not Calculated

Cox models adjusted for age, race/ethnicity, education level, poverty level, umber of primary care physician visits, NCI Charlson Comorbidity Index, hyperlipidemia, hypertension, diabetes, use of insulin, muscular wasting and disuse atrophy, malaise and fatigue, osteoporosis, and depressive disorder. Abbreviations: CI, confidence interval; HR, hazard ratio; TTh, testosterone replacement therapy. * SEER-Medicare data presentation guideline has been followed and all counts less than 11 have been suppressed.

## Data Availability

The SEER-Medicare datasets used to conduct this study are available upon approval of a research protocol by the National Cancer Institute. Instructions for obtaining these data are available at https://healthcaredelivery.cancer.gov/seermedicare/obtain (accessed on 10 July 2024). This study used the linked SEER-Medicare database. The interpretation and reporting of these data are the sole responsibility of the authors.

## Supplementary Materials

The following supporting information can be downloaded at: https://www.mdpi.com/article/10.3390/ph18091413/s1, Supplementary Material S1: SAS code for cohort selection. Supplementary Material S2: SAS code for 1:1 cohort matching. Supplementary Material S3: SAS code for statistical analysis.

## Abbreviations

The following abbreviations are used in this manuscript:

AJCC	American joint committee on cancer
BrCa	Breast cancer
CCI	Charlson comorbidity index
CI	Confidence interval
CRC	Colorectal cancer
CVD	Cardiovascular disease
DHEA	Dehydroepiandrosterone
EPIC	European prospective investigation into cancer and nutrition
FDA	Food and drug Aaministration
HMG-CoA	3-hydroxy-3-methylglutaryl coenzyme
HR	Hazard ratio
hs-CRP	High-sensitivity C-reactive protein
IRB	Institutional review board
LDL	Low-density lipoprotein
NDC	National drug code
ORC	Obesity-related cancer
PCOS	Polycystic ovary syndrome
PCP	Primary care physician
SD	Standard deviation
SHBG	Sex hormone-binding globulin
SEER	Surveillance, epidemiology, and end results
SES	Socioeconomic status
TTh	Testosterone replacement therapy
