# Supplementary material for: Obesity-Related Cancers in Relation to Use of Statins and Testosterone Replacement Therapy Among Older Women: SEER-Medicare 2007–2015
Source: Pharmaceuticals (Basel). 2025 Sep 19;18(9):1413. doi: 10.3390/ph18091413 (PMC12472775; doi:10.3390/ph18091413)
Supplement: Supplementary file 1 [file pharmaceuticals-18-01413-s001.zip › Supplementary material S1. SAS code for cohort selection.pdf]

Supplementary Material S1. SAS code for cohort selection

```
/*****************************************************************************
**************************************************/
/* DATE  : 07/17/2021
*/
/* AUTHOR: Biai Digbeu
*/
/* DATA  : Medicare SEER data
*/
/* Title: The effect of testosterone replacement therapy and statins on
cardiovascular diseases among hormone-related cancers
   in women: SEER-Medicare 2007-2016 Cohort selection (CASE)
*/
/*****************************************************************************
**************************************************/

/*Create libraries*/
libname SEER_c 'X:\';
libname SEER_nc 'W:\';
libname data 'Z:\Projects\Digbeu-B\Testosterone plus statins w CVDs among
cancer survivors women\Data';
libname mydata 'Z:\Projects\Digbeu-B\Testosterone plus statins w CVDs among
cancer survivors women\Mydata';
run;

proc format;
 value $ dc '0'='None'
            '1'='Statin alone'
                  '2'='Testosterone alone'
                  '3'='Both drugs';
run;


/* Denominator:
   Cases=Cancers: All females 65+ with cancer diagnosis between 2007-2016 and
continuous A/B/D enrollment 12 months before index date, and no CVD before
index date.
   Control=Non-cancers: All females 65+ at any time between 2007-2016,
matched with cancer cases on birth year checking at each step that the non-
cancers have A/B/D 12 months
                before matched index date and no CVD before index date
*/
%let data =pdesaf16;

proc contents varnum data=seer_c.&data;
run;

proc freq data=seer_c.&data;
tables PROD_SRVC_ID;
run;

proc freq data=seer_c.&data;
where linkflag='7';
tables modx1 yrdx1;
run;
```

```
/* Cases */
* 1.All female HRCs patients 65+ years of age with known HRC diagnosis
reported to SEER in 01/2007 - 09/2016
  Diagnosis date for HRC: If no month data assume January, day of month
assume 01;

data pedsf;
set seer_c.breast seer_c.colorectal seer_c.endometrial seer_c.ovarian;
 new = input(agedx1, 8.); /*convert 'character' to 'numeric' of age*/
   drop agedx1;
   rename new=agedx1;
if agedx1>= 65;
if m_sex='2';
if yrdx1 > 2006;
if src1 ne 6 and src1 ne 7; /*no autopsy or death certificate diagnosis*/
if modx1 = . then modx1 = "01";
dx_date = mdy(modx1,01,yrdx1);
format dx_date mmddyy.;
if siterwho1='26000' then bca=1;/*Breast cancer, n=218,496 women*/
if siterwho1 in ('21041' '21042' '21043' '21044' '21045' '21046' '21047'
'21048' '21049' '21051' '21052' '21060') then cca=1;   /*Colorectal cancer,
n=81,381 women*/
if siterwho1 in ('27040') then oca=1; /*Ovarian cancer, n=20,025 women */
if siterwho1 in ('27020' '27030') then eca=1; /*Endometrial cancer, n=39,601
women, new n=40866, +1265 observations*/
drop siterwho2-siterwho10; /*to remove duply patient_id */
if  bca=1 or cca=1 or oca=1 or eca=1;
run;
/* NOTE: The data set WORK.PEDSF has 360768 observations and 3564 variables.
*/

proc print data=pedsf;
var patient_id siterwho1 bca cca oca eca;
where bca=. and cca=. and oca=. and eca=.;
run;
/*NOTE: No observations were selected from data set WORK.PEDSF.*/

proc freq data=pedsf;
tables bca cca oca eca;
run;



/* Flag for more than 1 cancer count */

proc sql;
select count (distinct patient_id) from pedsf ;
quit;/*354460 unique patient ID*/
*Thus, this means there are patients with more than 1 cancer count in the
dataset. We decided to focus on first cancer diagnosis only;

proc sort data=pedsf;
by patient_id dx_date;
run;
```

```
data pedsf1;
set pedsf;
by patient_id;
if first.patient_id then count_cancer=0;
count_cancer+1;
run;

proc freq data=pedsf1;
tables count_cancer;
run;
/*
count_cancer Frequency Percent
           1    354460   98.25
           2      6247    1.73
           3        61    0.02
Exclude patients with more than 1 cancer (2 and 3)

*/
data pedsf1;
set pedsf1;
if count_cancer=1;
run;
/* NOTE: The data set WORK.PEDSF1 has 354460 observations and 3565 variables.
*/

proc sql;
select count (distinct patient_id) from pedsf1;
quit;
/*n= 354460*/

/* Cancer types */
data pedsf1;
set pedsf1;
if bca=1 then cancer=1;
else if cca=1 then cancer=2;
else if oca=1 then cancer=3;
else cancer=4;
run;

proc freq data=pedsf1;
tables bca cca oca eca;
tables cancer;
run;

/*
cancer                           Frequency           Percent
1=Breast cancer                  215,689              60.85
2=Colorectal cancer               79,805              22.51
3=Ovarian cancer                  19,542               5.51
4=Endometrial cancer              39,424              11.12
*/
proc contents varnum data=pedsf1;
run;
```

```
*2. Flag at least 1 year of continuous part D enrollment anytime between 2007
and 2015;

data pedsf2;
set pedsf1;
      /*array AB(*)  mon193-mon324;
      array HMO(*) gho193-gho324;
      array ENROLL(*) EN193-EN324;*/
      array PLAN(*) PLAN07_01--PLAN16_09; /*plan indicator (yes/no) for each
month from 2007-2016.Sep*/
      array PIND(*) PARTD1-PARTD117; /*initial continuous enrollment, part A
and B and no HMO enrollment for each month from 2007 to 2016.Sep*/

      /* do i=1 to dim(AB);
        if AB(i) in ("3") and HMO(i) in ("0" "4") then ENROLL(i)=1; else
ENROLL(i)=0;
       end; */
       /* TD=((year(dx_date)-2007)*12+month(dx_date)); */ /*turn dx_date to
months from 2007*/

      /* Con_ABDpre12m=0; */

       do i=1 to dim(PLAN);
           if PLAN(i) in ('H' 'R' 'S' 'E') then PIND(i)=1; else PIND(i)=0;
        end;

             /* if TD - 12 <=i<= TD then do; */       /*count months of
enrollment from 12 months before index date*/

        PartDsum=sum(of PARTD1-PARTD117);
              if PartDsum >=12  then IN_D12any=1; else IN_D12any=0;

      drop i;

run; /* N= 354,460 observations and 3684 variables. */

*check counts;
proc freq data=pedsf2;
 table IN_D12any;
run;
/*
                                              Cumulative    Cumulative
IN_D12any    Frequency     Percent     Frequency      Percent
        0        78364       22.11         78364        22.11
        1       276096       77.89        354460       100.00
*/

data mydata.cases;
 set pedsf2;
  if IN_D12any=1;
run;
/* NOTE: The data set MYDATA.CASES has 276096 observations and 3684
variables. */

/* 3. Flag for Cancer stages (stage I, II, III, IV) */
proc contents data=mydata.cases;
run;
```

```
proc print data=mydata.cases (obs=500);
var patient_id dajccstg1-dajccstg10;
run;
/*FYI: Use the first variable of the list (corresponds to cancer stage upon
initial cancer diagnosis */

proc freq data=mydata.cases;
tables dajccstg1;
run;

data mydata.cases;
set mydata.cases;
advanced_cancer_stage=0;
if dajccstg1 in ('50' '51' '52' '53' '54' '55' '56' '57' '58' '59' '60' '61'
'62' '63' '70' '71' '72' '73' '74')
then advanced_cancer_stage=1;
run;
/* NOTE: The data set MYDATA.CASES has 276096 observations and 3685
variables. */

proc freq data=mydata.cases;
tables advanced_cancer_stage;
run;


/*4. Cancer grades (advanced vs. non-advanced) */
proc freq data=mydata.cases;
table grade1-grade10;
run;
/*FYI: Use the first variable of the list (corresponds to cancer grade upon
initial cancer diagnosis */

proc freq data=mydata.cases;
table grade1;
run;

data mydata.cases;
set mydata.cases;
high_tumor_grade=0;
if grade1='3' then high_tumor_grade=1;
run;
/* NOTE: The data set MYDATA.CASES has 276096 observations and 3686
variables. */


proc freq data=mydata.cases;
table high_tumor_grade;
run;


proc print data=mydata.cases (obs=500);
var patient_id grade1-grade10;
run;
```

```
/*Control */
/*1. All female HRCs patients 65+ years of age in the SEER Non-cancer data in
01/2007 - 09/2016 */

data sumdenom;
set seer_nc.sumdenom;
if (2015 - birthyr)>=65;
if m_sex='2'; /*females from sumden file*/
run;
/*399287 controls aged 65+ any time before 2015 and 1628 variables*/

*2. Flag at least 1 year of continuous part D enrollment anytime between 2007
and 2015;

data sumdenom1;
set sumdenom;
      /*array AB(*)  mon193-mon324;
      array HMO(*) gho193-gho324;
      array ENROLL(*) EN193-EN324;*/
      array PLAN(*) PLAN07_01--PLAN16_09; /*plan indicator (yes/no) for each
month from 2007-2016.Sep*/
      array PIND(*) PARTD1-PARTD117; /*initial continuous enrollment, part A
and B and no HMO enrollment for each month from 2007 to 2016.Sep*/

      /* do i=1 to dim(AB);
        if AB(i) in ("3") and HMO(i) in ("0" "4") then ENROLL(i)=1; else
ENROLL(i)=0;
       end; */
       /* TD=((year(dx_date)-2007)*12+month(dx_date)); */ /*turn dx_date to
months from 2007*/

      /* Con_ABDpre12m=0; */

       do i=1 to dim(PLAN);
           if PLAN(i) in ('H' 'R' 'S' 'E') then PIND(i)=1; else PIND(i)=0;
        end;

             /* if TD - 12 <=i<= TD then do; */       /*count months of
enrollment from 12 months before index date*/

        PartDsum=sum(of PARTD1-PARTD117);
              if PartDsum >=12  then IN_D12any=1; else IN_D12any=0;

      drop i;

run;
/* N=399287 observations and 1746 variables.*/

*check counts;
proc freq data=sumdenom1;
 table IN_D12any;
run;
/*
                                                 Cumulative    Cumulative
IN_D12any    Frequency     Percent     Frequency      Percent
        0       98818       24.75         98818        24.75
        1      300469       75.25        399287       100.00
```

```
*/

data mydata.control;
 set sumdenom1;
  if IN_D12any=1;
run;
/* NOTE: The data set MYDATA.CONTROL has 300469 observations and 1746
variables. */

/* Append cases and control data alltogether */

/*proc sql;
create table cohort
as select * from mydata.cases
union all
select * from mydata.control;
quit;*/


/* 3a. Flag the first date of Testosterone Use ever among cases */
* Key note for TRT: NDC codes is used to define oral drug and CPT codes is
used to define injection drug.;

*---------------------------------------------- NDC codes -------------------
---------------------------;
/*List relevant NDC codes*/
proc sql;
create table tmp as
select  ndc,theraClassDesc, productname, theraclasscode, roacode,
masterformcode
from data.mastertheraclass
where upcase(theraClassDesc) like '%TESTOSTERONE%' or upcase(theraClassDesc)
like '%FLUOXYMESTERONE%';
quit; /*449 rows*/


/*Exlude the non-testosterone drug in the dataset*/
data tmp;
set tmp;
if ndc="52584025710" then delete; /* "ESTRADIOL CYPIONATE" */
run;/*448 rows*/

/*Create format with the NDC codes, purpose is to create an extra row with
blank ndc */
data myformatDat;
 retain fmtname 'testNDC' type 'c' start label;
set tmp(keep = ndc rename = (ndc = start)) end = eof;
 output;
 if eof then
 do;
   start = ' ';
   label = 'NO';
   HLO = 'O';
   output;
 end;
run;/*NOTE: The data set WORK.MYFORMATDAT has 449 observations and 5
variables.*/
```

```
proc format cntlin = myformatDat; run; /*cntlin=row count in proc format */
proc sql;
 create table tmp2 as
  select ndc, productName, put(ndc, $testNDC.) as testosterone
   from data.mastertheraclass;
quit;
/* NOTE: Table WORK.TMP2 created, with 344056 rows and 3 columns. */

proc print data=tmp2; where ndc="52584025710"; run; /*
NOTE: There were 1 observations read from the data set WORK.TMP2, Delete */

data tmp2;
set tmp2;
if ndc="52584025710" then delete;
run;
/* NOTE: The data set WORK.TMP2 has 344055 observations and 3 variables. */


/*All testosterone drug claims from linkage 16*/
data test_claims16;
set seer_c.pdesaf:;
where put(prod_srvc_id, $testNDC.) ^= 'NO';
run;/*NOTE: The data set WORK.TEST_CLAIMS16 has 74379 observations and 21
variables.*/

data mydata.test_claims16;
set test_claims16;
run;


proc sql;
select count (distinct prod_srvc_id) from test_claims16;
quit; /*n=70*/

proc sql;
select count (distinct patient_id) from test_claims16 where patient_id IN
(select patient_id from mydata.cases);
quit; /*147*/

/*All testosterone drug claims from non cancer*/
data test_claims_sum;
set seer_nc.pdesaf:;
where put(prod_srvc_id, $testNDC.) ^= 'NO';
run;
/*NOTE:  The data set WORK.TEST_CLAIMS_SUM has 94575 observations and 21
variables.*/

data mydata.test_claims_sum;
set test_claims_sum;
run;

proc sql; select count (distinct prod_srvc_id) from test_claims_sum ; quit;
/*72*/
proc sql; select count (distinct patient_id) from test_claims_sum where
patient_id IN (select patient_id from mydata.control); quit; /*162*/
```

```
/*Find injectable testosterone from CPT codes*/
/*Extract testosterone or statin injections data from cancer patients*/
data mydata.inj16;
set seer_c.nch: (keep=patient_id from_dtm from_dtd from_dty hcpcs_cd)
    seer_c.outsaf:(keep=patient_id from_dtm from_dtd from_dty hcpcs_cd);
if hcpcs_cd IN
('J0900','J1070','J1080','J1090','J310','J3130','J3140','J3150','S0189','G881
6','G9441','G9664','G9797');
run; /*NOTE: The data set MYDATA.INJ16 has 68676 observations and 5
variables.*/

/*Extract testosterone or statin injections data from non-cancer patients*/
data mydata.inj_non;
set seer_nc.nch: (keep=patient_id from_dtm from_dtd from_dty hcpcs_cd)
    seer_nc.outsaf:(keep=patient_id from_dtm from_dtd from_dty hcpcs_cd);
if hcpcs_cd IN
('J0900','J1070','J1080','J1090','J310','J3130','J3140','J3150','S0189','G881
6','G9441','G9664','G9797');
run; /*NOTE: The data set MYDATA.INJ_NON has 63506 observations and 5
variables.*/

proc sql; select count (distinct patient_id) from mydata.inj16
where patient_id IN (select patient_id from mydata.cases) and hcpcs_cd in
('J0900','J1070','J1080','J1090','J310','J3130','J3140','J3150','S0189')
and patient_id NOT IN  (select patient_id from test_claims16); quit; /*300
women with cancer that got injectable testosterone from linkage*/

proc sql; select count (distinct patient_id) from mydata.inj_non
where patient_id IN (select patient_id from mydata.control) and hcpcs_cd in
('J0900','J1070','J1080','J1090','J310','J3130','J3140','J3150','S0189')
and patient_id NOT IN  (select patient_id from test_claims_sum); quit; /*335
women without cancer that got injectable testosterone from linkage*/

/*Determine the first and last date of filled prescription and days supply of
last prescription for testosterone.
First append all files with testosterone claims together for all patients*/
proc sql;
 create table testosterone as
  select patient_id, srvc_mon, srvc_day, srvc_yr, days_suply_num, 'G' as
T_type
   from test_claims16 /* From NDC testosterone dataset for cancer patients */
 UNION ALL
   select patient_id, srvc_mon, srvc_day, srvc_yr, days_suply_num, 'G' as
T_type
   from test_claims_sum /* From NDC testosterone dataset for non-cancer
patients */
 UNION ALL
   select patient_id, from_dtm, from_dtd, from_dty, . as days_suply_num, 'I'
as T_type
   from mydata.inj16 /* From CPT codes/injection testosterone dataset for
cancer patients */
   where hcpcs_cd in
('J0900','J1070','J1080','J1090','J310','J3130','J3140','J3150','S0189')
 UNION ALL
   select patient_id, from_dtm, from_dtd, from_dty, . as days_suply_num, 'I'
as T_type
```

```
   from mydata.inj_non /* From CPT codes/injection testosterone dataset for
non-cancer patients */
   where hcpcs_cd in
('J0900','J1070','J1080','J1090','J310','J3130','J3140','J3150','S0189')
 ;quit;
/*Table WORK.TESTOSTERONE created, with 300862 rows and 6 columns.*/

data testosterone;
set testosterone;
fill_dt = mdy(srvc_mon, srvc_day, srvc_yr);
format fill_dt mmddyy.;
run;
/* NOTE: The data set WORK.TESTOSTERONE has 300862 observations and 7
variables. */

proc sql;
 create table testosterone_pat1 as
  select patient_id, min(fill_dt) as first_fill format mmddyy., max(fill_dt)
as last_fill format mmddyy., sum(days_suply_num) as days_sup
   from testosterone
    group by patient_id
;quit;
/* NOTE: Table WORK.TESTOSTERONE_PAT1 created, with 26857 rows and 4 columns.
*/

** Add variables(number of injection for testosterone);
proc sql;
 create table testosterone_pat2 as
  select patient_id, count(fill_dt) as inj_num
   from testosterone
    where T_type='I'
    group by patient_id
;quit; /* NOTE: Table WORK.TESTOSTERONE_PAT2 created, with 10223 rows and 2
columns. */

proc sort data=testosterone_pat1; by patient_id; run;
proc sort data=testosterone_pat2; by patient_id; run;

data testosterone_pat;
 merge testosterone_pat1 testosterone_pat2;
 by patient_id;
run;/* NOTE: The data set WORK.TESTOSTERONE_PAT has 26857 observations and 5
variables. */

proc sql;
select count (distinct patient_id) from testosterone_pat;
quit;
/*n=26857, no duplicates */

proc sql;
select count (distinct patient_id) from testosterone_pat
where patient_id IN (select patient_id from mydata.cases);
quit; /*447 women with HRC had testosterone prescription. */

proc sql;
select count (distinct patient_id) from testosterone_pat where patient_id IN
(select patient_id from mydata.control);
```

```
quit; /*497 women with non-HRC had testosterone prescription. */

/* Flag patients who ever had testosterone use between 2007 and 2015 in cases
and control dataset */


/* 3b. Flag the Statin users and First date of Statin use */

/*Find statin users and first date of statin use*/
proc sql;
create table tmps2 as
select  ndc,theraClassDesc, productname, theraclasscode
from data.mastertheraclass
where upcase(theraClassDesc) like '%STATIN%' and (theraclasscode) LIKE
('240%')
;quit; /* NOTE: Table WORK.TMPS2 created, with 2993 rows and 4 columns. */

/*Create format with the NDC codes*/
data myformatDat;
 retain fmtname 'statNDC' type 'c' start label;
 set tmps2(keep = ndc rename = (ndc = start)) end = eof;
 output;
 if eof then
 do;
   start = ' ';
   label = 'NO';
   HLO = 'O';
   output;
 end;
run;

proc format cntlin = myformatDat; run; /* NOTE: There were 2994 observations
read from the data set WORK.MYFORMATDAT. */

/*All statin claims from linkage 16 or cancer dataset*/
data stat_claims16;
set seer_c.pdesaf:;
where put(prod_srvc_id, $statNDC.) ^= 'NO';
run;
/* NOTE: The data set WORK.STAT_CLAIMS16 has 12599772 observations and 21
variables. */


proc sql;
select count (distinct prod_srvc_id) from stat_claims16;
quit;/*1081 (total ndc statin count)*/

proc sql;
select count (distinct patient_id) from stat_claims16 where patient_id IN
(select patient_id from mydata.cases);
quit; /*169532  women with cancer who were prescribed with statin. */


/*All statin claims from non cancer*/
data stat_claims_sum;
set seer_nc.pdesaf:;
where put(prod_srvc_id, $statNDC.) ^= 'NO';
```

```
run;
/* NOTE: The data set WORK.STAT_CLAIMS_SUM has 9529869 observations and 21
variables. */

proc sql;
select count (distinct prod_srvc_id) from stat_claims_sum ;
quit; /* 1059 (total ndc statin count) */

proc sql;
select count (distinct patient_id) from stat_claims_sum where patient_id IN
(select patient_id from mydata.control);
quit; /*178741 women with non-cancer who were prescribed with statin. */


/*count those that only got injection*/
proc sql;
select count (distinct patient_id) from mydata.inj16
where patient_id IN (select patient_id from mydata.cases) and hcpcs_cd in
('G8816','G9441','G9664')/*Code G9797 has no patients anyway*/
and patient_id NOT IN  (select patient_id from stat_claims16); /* but also is
not certain to mean statin use */
quit; /* 2 patients with cancer and statin injection.  */


proc sql;
select count (distinct patient_id) from mydata.inj_non
where patient_id IN (select patient_id from mydata.control) and hcpcs_cd in
('G8816','G9441','G9664')
and patient_id NOT IN  (select patient_id from stat_claims_sum);
quit; /* 3 patients without cancer and statin injection. */


/*Append all files with statin claims together for all patients*/
proc sql;
 create table statin as
  select patient_id, srvc_mon, srvc_day, srvc_yr, days_suply_num
   from stat_claims16 /* From ndc statin with cancer */
 UNION ALL
   select patient_id, srvc_mon, srvc_day, srvc_yr, days_suply_num
   from stat_claims_sum /* From ndc statin without cancer */
 UNION ALL
   select patient_id, from_dtm, from_dtd, from_dty, . as days_suply_num
/*injection is always 'one' time, thus days of supply is missing*/
   from mydata.inj16 /* From CPT/injection statin with cancer */
   where hcpcs_cd in ('G8816','G9441','G9664')
 UNION ALL
   select patient_id, from_dtm, from_dtd, from_dty, . as days_suply_num
   from mydata.inj_non  /* From CPT/injection statin without cancer */
   where hcpcs_cd in ('G8816','G9441','G9664');
quit;
/* NOTE: Table WORK.STATIN created, with 22129915 rows and 5 columns. */

data statin;
set statin;
fill_dt = mdy(srvc_mon, srvc_day, srvc_yr);
format fill_dt mmddyy.;
run;
```

```
/* NOTE: The data set WORK.STATIN has 22129915 observations and 6 variables.
*/

proc sql;
 create table statin_pat as
  select patient_id, min(fill_dt) as first_fill format mmddyy., max(fill_dt)
as last_fill format mmddyy., sum(days_suply_num) as days_sup, count(fill_dt)
as inj_num
   from statin
    group by patient_id;
quit;
/* NOTE: Table WORK.STATIN_PAT created, with 846233 rows and 5 columns. */

*add 3/18 to draw the date of Statin;
proc sql;
 create table statin_pat2 as
  select patient_id, min(fill_dt) as S_first_fill format mmddyy.
   from statin
    group by patient_id;
quit; /* NOTE: Table WORK.STATIN_PAT2 created, with 846233 rows and 2
columns. */

data mydata.testosterone_pat; set testosterone_pat; run;

data mydata.statin_pat; set statin_pat; run;

/* 3c. Find date of first use of each drug or first date of concurrent use if
using both */                ;

proc sql;
 create table drugs as
  select *, "T" as drug
   from testosterone_pat
  UNION ALL
  select *, "S" as drug
   from statin_pat
  order by patient_id;
quit;
/* NOTE: Table WORK.DRUGS created, with 873090 rows and 6 columns. */

proc sort data=drugs out=a(keep=patient_id days_sup drug);
by patient_id drug;
run;

proc transpose data=a out=b;
by patient_id;
var days_sup;
run;

data drugs_sup;
set b;
 rename COL1=S_sup;
 rename COL2=T_sup;
 drop _NAME_;
run; /* n=855953 */
```

```
proc sql;
 create table drugs2 as
  select patient_id, max(last_fill) as last_drug_dt format mmddyy.,
min(first_fill) as first_drug_dt format mmddyy.,
         count(distinct drug) as count_drug, max(first_fill) as
second_drug_dt format mmddyy.,
         max(case when drug="T" then 1 else 0 end) as Testosterone,
         max(case when drug="S" then 1 else 0 end) as Statin, min(days_sup)
as supply_days
  from drugs
  group by patient_id;
quit; /* NOTE: Table WORK.DRUGS2 created, with 855953 rows and 8 columns. */
/*Second_drug_dt is different that first_drug_dt only when two drugs taken.
  -> Min supply date is the duration of concurrent drug use if both drugs,
     otherwise it will be the same as max(days_sup) for those with one drug*/

/*Add a column with the first drug used (needed for those with two drugs)*/
proc sql;
 create table drugs3 as
  select a.*, b.drug as First_drug
   from drugs2 as a
    left join (select patient_id, drug, first_fill from drugs group by
patient_id having first_fill = min(first_fill)) as b
     on a.patient_id = b.patient_id
     and a.first_drug_dt = b.first_fill;
quit; /* NOTE: Table WORK.DRUGS3 created, with 856321 rows and 9 columns. */

proc sql;
 create table junk2 as
  select *
  from drugs
  group by patient_id having count(patient_id) > 1 and
max(first_fill)=min(first_fill);
quit; /*There are 736 patients with S and T first fill at the same date.
         That is why there are more rows in drugs3. For those we need to put
first_drug=B/both*/

proc sql;
 create table drugs4 as
  select patient_id, min(first_drug_dt) as first_drug_dt format mmddyy.,
max(second_drug_dt) as second_drug_dt format mmddyy.,
         max(count_drug) as count_drug, max(Testosterone) as Testosterone,
max(Statin) as Statin, First_drug,
         /*max(case when (count_drug=2) then "B" else First_drug end) as
First_drug,*/
         max(supply_days) as supply_days, max(last_drug_dt) as last_drug_dt
format mmddyy.
   from drugs3
   group by patient_id;
quit; /* NOTE: Table WORK.DRUGS4 created, with  856321 rows and 9 columns. */

/*proc freq data=drugs4;
 tables count_drug first_drug count_drug*first_drug;
run;*//*OK*/

proc sort data=drugs_sup;
by patient_id;
```

```
run;

proc sort data=drugs4;
by patient_id;
run;

/*Create index date based on first date of drug use if 1 drug and based on
first date of first drug used if 2 drugs */
data mydata.drugs_all; /*All drugs before any restrictions*/
 merge drugs_sup drugs4;
 by patient_id;

if testosterone=1 and statin=1 then both_drugs=1; else both_drugs=0;

if first_drug='T' then do;
testosterone_first_dt=first_drug_dt;
testosterone_last_dt=last_drug_dt;
index_dt=testosterone_first_dt;
end;

if first_drug='S' then do;
statin_first_dt=first_drug_dt;
statin_last_dt=last_drug_dt;
index_dt=statin_first_dt;
end;

if both_drugs=1 then do;

if first_drug='T' then do;
testosterone_first_dt=first_drug_dt;
testosterone_last_dt=last_drug_dt;
index_dt=testosterone_first_dt;
statin_first_dt=second_drug_dt;


if first_drug='S' then do;
statin_first_dt=first_drug_dt;
statin_last_dt=last_drug_dt;
index_dt=statin_first_dt;
testosterone_first_dt=second_drug_dt;
end;
 end;
  end;

format testosterone_first_dt mmddyy10.;
format testosterone_last_dt mmddyy10.;
format statin_first_dt mmddyy10.;
format statin_last_dt mmddyy10.;
format index_dt mmddyy10.;

run; /* n=856321 */

proc sql;
select count (distinct patient_id) from mydata.drugs_all;
quit;
/*n=855953, has duplicates remove them */
```

```
proc sort data=mydata.drugs_all nodupkey out=mydata.drugs_all1;
by patient_id descending both_drugs index_dt;
run;
/* NOTE: The data set MYDATA.DRUGS_ALL1 has 855953 observations and 17
variables. */


/* Merge the drug dataset with cases and control datasets to flag for drug
exposed patients in each dataset */
proc sql;
select count (distinct patient_id) from mydata.cases;
quit;
/* n=276096, no duplicates*/

proc sql;
create table mydata.cases1
as select B.*, A.index_dt, A.Testosterone, A.T_sup, A.Statin, A.S_sup,
A.both_drugs, A.supply_days, A.first_drug,
               A.testosterone_first_dt, testosterone_last_dt,
statin_first_dt, statin_last_dt,
               case when '01JUL2007'd <= index_dt <= '30JUN2015'd then 1 else
0
               end
               as exposed
          from mydata.drugs_all1 as A right join mydata.cases as B
on B.patient_id=A.patient_id;
quit;
/* NOTE: Table MYDATA.CASES1 created, with 276096 rows and 3699 columns. */

proc freq data=mydata.cases1;
tables exposed testosterone statin both_drugs exposed*(exposed testosterone
statin both_drugs);
run;

/*
exposed    Frequency      Percent       Cumulative Frequency
Cumulative Percent
      0       175666        63.62               175666
63.62
      1       100430        36.38               276096
100.00
*/

proc sql;
select count (distinct patient_id) from mydata.control;
quit;
/* n=300469, no duplicates*/

proc sql;
create table mydata.control1
as select B.*, A.index_dt, A.Testosterone, A.T_sup, A.Statin, A.S_sup,
A.both_drugs, A.supply_days, A.first_drug,
               A.testosterone_first_dt, testosterone_last_dt,
statin_first_dt, statin_last_dt,
               case when '01JUL2007'd <= index_dt <= '30JUN2015'd then 1 else
0
               end
```

```
                as exposed
          from mydata.drugs_all1 as A right join mydata.control as B
on B.patient_id=A.patient_id;
quit;
/* NOTE: Table MYDATA.CONTROL1 created, with 300469 rows and 1759 columns. */

proc freq data=mydata.control1;
tables exposed testosterone statin both_drugs exposed*(exposed testosterone
statin both_drugs);
run;



/*
exposed    Frequency      Percent       Cumulative Frequency
Cumulative Percent
      0       190465        63.39              190465
63.39
      1       110004        36.61              300469
100.00
*/

/* Create analytical dataset by appending cases and control datasets and by
selecting only the relevant variables */
*Check for duplicate patient id in both cases and control dataset;

proc sql;
create table check_
as select patient_id from mydata.cases
where patient_id in (select patient_id from mydata.control);
quit;
/* NOTE: Table WORK.CHECK_ created, with 0 rows and 1 columns.,
no duplicate patient id in both cases and control datasets */
proc contents varnum data=mydata.cases1;
run;
proc contents varnum data=mydata.control1;
run;

data mydata.pre_cohort;
set mydata.cases1(keep=patient_id birthm birthyr VRFYDTH vsrtdx: odthclass:
med_dodm med_dodd med_dody m_sex race linkflag zip:
                       state: cnty: mon: gho: allflag: plan: partDsum dual:
                       agedx1 dx_date bca cca oca eca count_cancer cancer
advanced_cancer_stage high_tumor_grade
                       index_dt Testosterone T_sup statin S_sup both_drugs
supply_days First_drug testosterone_first_dt testosterone_last_dt
statin_first_dt statin_last_dt
                       exposed)
mydata.control1(keep=patient_id birthm birthyr VRFYDTH med_dodm med_dodd
med_dody m_sex race linkflag zip:
                     state: cnty: mon: gho: allflag: plan: partDsum dual:
                     index_dt Testosterone T_sup statin S_sup both_drugs
supply_days First_drug testosterone_first_dt testosterone_last_dt
statin_first_dt statin_last_dt
                     exposed);
run;
/*
```

```
NOTE: There were 276096 observations read from the data set MYDATA.CASES1.
NOTE: There were 300469 observations read from the data set MYDATA.CONTROL1.
NOTE: The data set MYDATA.PRE_COHORT has 576565 observations and 1444
variables. */

data mydata.pre_cohort;
set mydata.pre_cohort;
if dx_date=. then cancer_diagnosed=0; else cancer_diagnosed=1;
index_dt_new=mdy(month(index_dt),01,year(index_dt));
run;
/* NOTE: The data set MYDATA.PRE_COHORT has 576565 observations and 1446
variables.*/

proc sort data=mydata.pre_cohort;
by patient_id exposed index_dt_new;
run;

proc sql;
select count (distinct patient_id) from mydata.pre_cohort;
quit;
/* n=576565, no duplicates*/

proc freq data=mydata.pre_cohort;
table testosterone statin exposed*(testosterone statin) exposed;
run;

/*
exposed    Frequency      Percent       Cumulative Frequency
Cumulative Percent
      0       366131        63.50              366131
63.50
      1       210434        36.50              576565
100.00
*/

/* Separate by exposed vs unexposed patients for matching by birthyear */
data mydata.exposed mydata.unexposed;
set mydata.pre_cohort;
if exposed=1 then output mydata.exposed;
if exposed=0 then output mydata.unexposed;
run;
/*
NOTE: There were 576565 observations read from the data set
MYDATA.PRE_COHORT.
NOTE: The data set MYDATA.EXPOSED has 210434 observations and 1446 variables.
NOTE: The data set MYDATA.UNEXPOSED has 366131 observations and 1446
variables.
*/

proc freq data=mydata.exposed;
table index_dt index_dt_new cancer_diagnosed testosterone statin
both_drugs;/* OK */
run;

**In the unexposed dataset, exclude patients who were considered as unexposed
because they were exposed to the drug
  between 01/2006 to 06/2007 and between 07/2015 to 12/2016;
```

```
proc contents data=mydata.unexposed varnum; run;

proc freq data=mydata.unexposed;
table index_dt index_dt_new cancer_diagnosed;
run;

data mydata.unexposed;
set mydata.unexposed;
if index_dt ne . then delete;
run;
/* NOTE: The data set MYDATA.UNEXPOSED has 227923 observations and 1446
variables. */

proc freq data=mydata.unexposed;
table index_dt index_dt_new cancer_diagnosed;/* OK */
run;



/*
Inclusion/Exclusion criteria for exposed patients:
1- Age >=65 at prescription (index date)
2- Continuous AB enrollment 6 months prior to first prescription (index date)
3- No HRC diagnosis or HRC diagnosis >=6 months after index date;
*/

*1- Age >=65 at prescription (index date);
data exposed;
set mydata.exposed;
format index_dt_new mmddyy10.;
year_index_dt_new=year(index_dt_new);
age_exposed=year_index_dt_new-birthyr;
if age_exposed >= 65;
run;
/*
NOTE: There were 210434 observations read from the data set MYDATA.EXPOSED.
NOTE: The data set WORK.EXPOSED has 204394 observations and 1448 variables
*/

proc freq data=exposed;
table age_exposed cancer_diagnosed testosterone statin both_drugs
index_dt_new;
run;
/*OK*/

*2- Continuous AB enrollment 6 months prior to first prescription (index
date);
data exposed1;
set exposed;

TD=((year(index_dt_new)-2007)*12+month(index_dt_new)); /*turn dx_date to
months from 2007*/
Con_ABpre6m=0;

array AB(*)  mon193-mon324;
      array HMO(*) gho193-gho324;
```

```
      array ENROLL(*) EN193-EN324;

       do i=1 to dim(AB);
        if AB(i) in ("3") and HMO(i) in ("0" "4") then ENROLL(i)=1; else
ENROLL(i)=0;

       if TD - 6 <=i<= TD then do;        /*count months of enrollment from 6
months before index date*/
              if ENROLL(i)=1 then Con_ABpre6m+1;
                                             end;
        end;

      if Con_ABpre6m >= 6 then IN_ABpre6m=1;else IN_ABpre6m=0;
drop i;
run;
/* NOTE: The data set WORK.EXPOSED1 has 204394 observations and 1583
variables. */


proc freq data=exposed1;
table IN_ABpre6m TD;
run;

data exposed2;
set exposed1;
if IN_ABpre6m=1;
run;
/*
NOTE: There were 204394 observations read from the data set WORK.EXPOSED1.
NOTE: The data set WORK.EXPOSED2 has 97220 observations and 1583 variables.
*/

*3- No HRC diagnosis or HRC diagnosis >=6 months after index date;

data exposed3;
set exposed2;
if cancer_diagnosed=1;
run;
/* NOTE: The data set WORK.EXPOSED3 has 50056 observations and 1583
variables. */

data check2;
set exposed3;
if index_dt_new <= dx_date then true=1; else true=0;
run;

proc freq data=check2;
table true;
run;

/*
true    Frequency     Percent     Cumulative Frequency       Cumulative
Percent
   0        21730       43.41             21730                        43.41
   1        28326       56.59             50056                       100.00
*/
```

```
/* FYI: Some index date occured after cancer diagnosis, so those patients
will be excluded anyways */

data qual nonqual;
set exposed3;
gap_index_diagnosis=dx_date-index_dt_new;
if index_dt_new <= dx_date and gap_index_diagnosis >=183 then output qual;
else output nonqual;
run;
/*
NOTE: There were 50056 observations read from the data set WORK.EXPOSED3.
NOTE: The data set WORK.QUAL has 24230 observations and 1584 variables.
NOTE: The data set WORK.NONQUAL has 25826 observations and 1584 variables.
*/

proc sql;
create table exposed4
as select * from exposed2
where patient_id not in (select patient_id from nonqual);
quit;
/* NOTE: Table WORK.EXPOSED4 created, with 71394 rows and 1583 columns. */

proc freq data=exposed4;
table testosterone statin both_drugs exposed*(testosterone statin
both_drugs);
run;

proc freq data=exposed2;
table cancer_diagnosed;
run;

proc freq data=exposed4;
table cancer_diagnosed;
run;

data mydata.exposed1;
set exposed4;
format index_dt_new mmddyy10.;
run;
/* NOTE: The data set MYDATA.EXPOSED1 has 71394 observations and 1583
variables. */

/*
Inclusion/Exclusion criteria for unexposed patients:
1- Pool of eligible unexposed with at least 6 months of AB enrollment and age
>=65
*/

*Pool of eligible unexposed with at least 6 months of continuous AB
enrollment;
data unexposed1;
set mydata.unexposed;

    array AB(*)  mon193-mon324;
      array HMO(*) gho193-gho324;
      array ENROLL(*) EN193-EN324;
```

```
      do i=1 to dim(AB);
        if AB(i) in ("3") and HMO(i) in ("0" "4") then ENROLL(i)=1; else
ENROLL(i)=0;
       end;

        Con_AB6m=sum(of EN193-EN324);
              if Con_AB6m >=6  then IN_AB6any=1; else IN_AB6any=0;

drop i;
run;
/* NOTE: The data set WORK.UNEXPOSED1 has 227923 observations and 1580
variables. */

proc freq data=unexposed1;
table IN_AB6any;
run;

data mydata.unexposed1;
set unexposed1;
if IN_AB6any=1;
format index_dt_new mmddyy10.;
run;
/* NOTE: The data set MYDATA.UNEXPOSED1 has 164188 observations and 1580
variables. */
```
