# Supplementary material for: Obesity-Related Cancers in Relation to Use of Statins and Testosterone Replacement Therapy Among Older Women: SEER-Medicare 2007–2015
Source: Pharmaceuticals (Basel). 2025 Sep 19;18(9):1413. doi: 10.3390/ph18091413 (PMC12472775; doi:10.3390/ph18091413)
Supplement: Supplementary file 1 [file pharmaceuticals-18-01413-s001.zip › Supplementary material S2. Cohort matching.pdf]

Supplementary Material S2. SAS code for 1:1 cohort matching

```
/*****************************************************************************
**************************************************/
/* DATE  : 07/17/2021
*/
/* AUTHOR: Biai Digbeu
*/
/* DATA  : Medicare SEER data
*/
/* Title: The effect of testosterone replacement therapy and statins on
cardiovascular diseases among hormone-related cancers
   in women: SEER-Medicare 2007-2016 Cohort selection (CASE)
*/
/*****************************************************************************
**************************************************/

/*Create libraries*/
libname SEER_c 'X:\';
libname SEER_nc 'W:\';
libname data 'Z:\Projects\Digbeu-B\Testosterone plus statins w CVDs among
cancer survivors women\Data';
libname mydata 'Z:\Projects\Digbeu-B\Testosterone plus statins w CVDs among
cancer survivors women\Mydata';
run;

proc format;
 value $ dc '0'='None'
            '1'='Statin alone'
                  '2'='Testosterone alone'
                  '3'='Both drugs';
run;


/* Denominator:
   Cases=Cancers: All females 65+ with cancer diagnosis between 2007-2016 and
continuous A/B/D enrollment 12 months before index date, and no CVD before
index date.
   Control=Non-cancers: All females 65+ at any time between 2007-2016,
matched with cancer cases on birth year checking at each step that the non-
cancers have A/B/D 12 months
                before matched index date and no CVD before index date
*/

proc freq data=mydata.unexposed1; table startd Ddt partDdt dod;
run;

proc freq data=mydata.unexposed1; table dx_date;
run;


proc sort data=mydata.exposed1 out =exposed;
      by birthyr;
run;
/* NOTE: The data set WORK.EXPOSED has 71394 observations and 1588 variables.
*/
```

```
data exposed;
      format patient_id match_id index_dt_new ;
      set exposed;
      match_id + 1;
run;
/* NOTE: The data set WORK.EXPOSED has 71394 observations and 1589 variables.
*/

data ids; ***Exposed match id;
      format patient_id match_id index_dt_new birthyr low high partDdt;
      set exposed(keep = patient_id match_id index_dt_new birthyr partDdt);
      high = intck("month", "01JUL2006"d - 1, index_dt_new);
      low = high - 6;
      rename patient_id = exposed_id;
run;
/* NOTE: The data set WORK.IDS has 71394 observations and 7 variables. */


data pool_id; ***Unexposed match id;
      format unexposed_id birthyr dod1 dx_date1 partDdt1;
      if _n_ = 1 then do;
            dcl hash byear(dataset:"ids");
            byear.definekey("birthyr");
            byear.definedone();
      end;
      set mydata.unexposed1(
            keep=patient_id birthyr dod dx_date partDdt mon187--gho324
            rename=(patient_id = unexposed_id partDdt = partDdt1 dod = dod1
dx_date = dx_date1));
      if byear.find() = 0;
run;
/*
NOTE: There were 71394 observations read from the data set WORK.IDS.
NOTE: There were 164188 observations read from the data set
MYDATA.UNEXPOSED1.
NOTE: The data set WORK.POOL_ID has 164104 observations and 467 variables.
 */

data pool1;
      set pool_id;
      call streaminit(1);
      u = rand("Uniform");
run;
/* NOTE: The data set WORK.POOL1 has 164104 observations and 468 variables.
*/


proc sort data = pool1 out = pool1(drop = u);
      by birthyr u;
run;
/* NOTE: The data set WORK.POOL1 has 164104 observations and 467 variables.
*/
```

```
*1:1 match;

%let matches = 1;

data matched;
      keep exposed_id -- partDdt1 gho324;
      if _n_ = 0 then set ids pool1;
      if _n_ = 1 then do;
            dcl hash idyr(dataset:"pool_id", multidata:"yes", hashexp:20);
            idyr.definekey("birthyr");
            idyr.definedata("unexposed_id");
            idyr.definedone();

            dcl hash insurance(dataset:"pool1", hashexp:20);
            insurance.definekey("unexposed_id");
            insurance.definedata(all:"yes");
            insurance.definedone();
      end;
      set ids;
      array AB  mon187-mon324;
      array HMO gho187-gho324;
      rc = idyr.find();
      matches = 0;
      do while (matches < &matches and rc = 0);
            if insurance.find() or (partDdt1 - index_dt_new) > 1 or
            min("31DEC2015"d, dod1, dx_date1) - index_dt_new < 183
            then rc = idyr.find_next();
            else do;
                partAB = 1;
                  do i = low to high while (partAB);
                        if AB[i] not in ("3") or HMO[i] not in ("0" "4") then
do;
                              partAB = 0;
                              leave;
                        end;
                  end;
                  if partAB = 0 then rc = idyr.find_next();
                  else do;
                        matches + 1;
                        rc1 = insurance.remove();
                        output matched;
                  end;
            end;
      end;
run;
/*
NOTE: There were 164104 observations read from the data set WORK.POOL_ID.
NOTE: There were 164104 observations read from the data set WORK.POOL1.
NOTE: There were 71394 observations read from the data set WORK.IDS.
NOTE: The data set WORK.MATCHED has 71386 observations and 12 variables.
*/
```

```
data mydata.matched1;
set matched;
run;
/* NOTE: The data set MYDATA.MATCHED1 has 71386 observations and 12
variables. */



proc sql;
select count(distinct exposed_id) from mydata.matched1;
quit;
/*n=71386*/

proc sql;
select count(distinct unexposed_id) from mydata.matched1;
quit;
/*n=71386*/

proc contents data=mydata.matched1; run;

proc sql;
create table check_matched
as select *, count(match_id) as match_count from matched
group by match_id;
quit;

proc freq data=check_matched;
table match_id match_count;
run;


/*
data unexposed;
      format patient_id match_id index_dt_new;
      if _n_ = 0 then set matched(keep = unexposed_id index_dt_new match_id);
      if _n_ = 1 then do;
            dcl hash ids(dataset:"matched");
            ids.definekey("unexposed_id");
            ids.definedata("index_dt_new", "match_id");
            ids.definedone();
      end;
      set mydata.unexposed1(drop = mon187--gho324);
      if ids.find(key:patient_id) = 0 then output;
      drop unexposed_id;
run;*/
/* NOTE: The data set WORK.UNEXPOSED has 71388 observations and 1124
variables */

/*
data matched_a;
      set exposed unexposed;
run;
*/
/*
NOTE: There were 71394 observations read from the data set WORK.EXPOSED.
NOTE: There were 71388 observations read from the data set WORK.UNEXPOSED.
```

```
NOTE: The data set WORK.MATCHED_A has 142782 observations and 1591 variables.
*/

/*
proc sort data=matched;
by match_id;
run;
*/


/* Build the analytical dataset based on the matched dataset(1:1) */


**Exposed;
proc sql;
create table mydata.exposed2_1 as select * from mydata.exposed1
where patient_id in (select exposed_id from mydata.matched1);
quit;
/* NOTE: Table MYDATA.EXPOSED2_1 created, with 71386 rows and 1588 columns.
*/

/* Select match_id from the matched dataset for the exposed patients */

proc sort data=mydata.matched1 out=matched1_a nodupkey;
by exposed_id;
run;
/* NOTE: The data set WORK.MATCHED1_A has 71386 observations and 12
variables. */

proc sql;
create table mydata.exposed3_1
as select B.*, A.match_id from matched1_a as A right join mydata.exposed2_1
as B
on B.patient_id=A.exposed_id;
quit;
/* NOTE: Table MYDATA.EXPOSED3_1 created, with 71386 rows and 1589 columns.
*/

**Unexposed;
proc sql;
create table mydata.unexposed2_1 as select * from mydata.unexposed1
where patient_id in (select unexposed_id from mydata.matched1);
quit;
/* NOTE: Table MYDATA.UNEXPOSED2_1 created, with 71386 rows and 1585 columns.
*/

proc freq data=mydata.unexposed2_1;
table index_dt index_dt_new;
run;
/* All index_dt and index_dt_new are blank. */ /* OK */

data mydata.unexposed2_1;
set mydata.unexposed2_1;
drop index_dt index_dt_new;
run;
/* NOTE: The data set MYDATA.UNEXPOSED2_1 has 71386 observations and 1583
variables. */
```

```
proc sort data=mydata.matched1 out=matched1_b nodupkey;
by unexposed_id;
run;
/* NOTE: The data set WORK.MATCHED1_B has 71386 observations and 12
variables. */

proc sql;
create table mydata.unexposed3_1
as select B.*, A.match_id, A.index_dt_new from matched1_b as A right join
mydata.unexposed2_1 as B
on B.patient_id=A.unexposed_id;
quit;
/* NOTE: Table MYDATA.UNEXPOSED3_1 created, with 71386 rows and 1585 columns.
*/




/* Select match_id from the matched dataset for the unexposed patients */


proc contents varnum data=mydata.exposed3_1;
run;

proc contents varnum data=mydata.unexposed3_1;
run;

data cohort_1;
set mydata.exposed3_1(keep=patient_id linkflag birthm birthyr med_dodm
med_dodd med_dody dod vrfydth vsrtdx: odthclass: m_sex race agedx1 dx_date
bca cca oca eca cancer
                          advanced_cancer_stage
                          high_tumor_grade index_dt index_dt_new
year_index_dt_new Testosterone T_sup Statin S_sup both_drugs supply_days
First_drug testosterone_first_dt testosterone_last_dt
                          statin_first_dt statin_last_dt exposed
cancer_diagnosed age_exposed partDdt match_id)

    mydata.unexposed3_1(keep=patient_id linkflag birthm birthyr med_dodm
med_dodd med_dody dod vrfydth vsrtdx: odthclass:
                            m_sex race agedx1 dx_date bca cca oca eca cancer
advanced_cancer_stage
                            high_tumor_grade index_dt_new Testosterone T_sup
Statin S_sup both_drugs supply_days First_drug testosterone_first_dt
testosterone_last_dt
                           statin_first_dt statin_last_dt exposed
cancer_diagnosed partDdt match_id);
run;

/*
NOTE: There were 71386 observations read from the data set MYDATA.EXPOSED3_1.
NOTE: There were 71386 observations read from the data set
MYDATA.UNEXPOSED3_1.
NOTE: The data set WORK.COHORT_1 has 142772 observations and 59 variables.
*/
```

```
proc freq data=cohort_1;
table exposed;
run;
/*
exposed    Frequency   Percent    Cumulative Frequency     Cumulative Percent
      0        71386     50.00             71386                   50.00
      1        71386     50.00            142772                  100.00
*/

proc sort data=cohort_1;
by match_id descending exposed;
run;
/* NOTE: The data set WORK.COHORT_1 has 142772 observations and 59 variables.
*/

data mydata.cohort_1;
set cohort_1;
run;
/* NOTE: The data set MYDATA.COHORT_1 has 142772 observations and 59
variables. */


data cohort_1;
set mydata.cohort_1;
run;

proc freq data=cohort_1;
where exposed=1;
table cancer_diagnosed;
run;

/*
cancer_diagnosed   Frequency    Percent   Cumulative Frequency   Cumulative
Percent
               0       47156      66.06          47156
66.06
               1       24230      33.94          71386
100.00
*/


proc freq data=cohort_1;
where exposed=0;
table cancer_diagnosed;
run;

/*
cancer_diagnosed   Frequency    Percent   Cumulative Frequency   Cumulative
Percent
               0       43530      60.98          43530
60.98
               1       27856      39.02          71386
100.00
*/
```

```
/* Double check that gap between exposure and cancer diagnosis is at least 6
months */
data cohort_1;
set cohort_1;
gap=dx_date-index_dt_new;
run;
/* NOTE: The data set WORK.COHORT_1 has 142772 observations and 60 variables.
*/

proc means data=cohort_1;
var gap;
run;
/*N=52086, OK, FYI:consits of all cancer diagnosed in the dataset */

proc means data=cohort_1;
where exposed=1;
var gap;
run;
/*N=24230, OK, FYI:consists of cancer diagnosed only among the exposed*/

proc means data=cohort_1;
where exposed=0;
var gap;
run;
/*N=27856, OK, FYI:consists of cancer diagnosed only among the unexposed*/

proc freq data=cohort_1;
where exposed=1;
table testosterone statin both_drugs; /* OK */
run;

proc freq data=cohort_1;
where exposed=0;
table testosterone statin both_drugs; /* Blank. */
run;

data mydata.cohort_1;
set cohort_1;
run;
/* NOTE: The data set MYDATA.COHORT_1 has 142772 observations and 60
variables. */

/* Extract all claims of interest in the 6 months before index date */

/*proc sql;
 create table verif as
  select a.patient_id, a.index_dt_new, b.admsndtm, b.admsndtd, b.admsndty,
         b.DGNSCD1, b.DGNSCD2, b.DGNSCD3, b.DGNSCD4, b.DGNSCD5,
             b.DGNSCD6, b.DGNSCD7, b.DGNSCD8, b.DGNSCD9, b.DGNSCD10,
         b.DGNSCD11, b.DGNSCD12, b.DGNSCD13, b.DGNSCD14, b.DGNSCD15,
             b.DGNSCD16, b.DGNSCD17, b.DGNSCD18, b.DGNSCD19, b.DGNSCD20,
         b.DGNSCD21, b.DGNSCD22, b.DGNSCD23, b.DGNSCD24, b.DGNSCD25
   from mydata.cohort_1 as a right join seer_c.medpar10 as b
    on a.patient_id = b.patient_id
   where a.index_dt_new - 183 <= mdy(input(b.admsndtm, 2.), 01,
input(b.admsndty, 4.)) <= a.index_dt_new;
quit;*/
```

```
*Inpatient claims;

**Cancer;

%macro inpatient(data);
%do YR=6 %TO 9;

proc sql;
create table wo&YR as
  select a.patient_id, a.index_dt_new, b.admsndtm, b.admsndtd, b.admsndty,
         b.DGNSCD1, b.DGNSCD2, b.DGNSCD3, b.DGNSCD4, b.DGNSCD5,
             b.DGNSCD6, b.DGNSCD7, b.DGNSCD8, b.DGNSCD9, b.DGNSCD10,
         b.DGNSCD11, b.DGNSCD12, b.DGNSCD13, b.DGNSCD14, b.DGNSCD15,
             b.DGNSCD16, b.DGNSCD17, b.DGNSCD18, b.DGNSCD19, b.DGNSCD20,
         b.DGNSCD21, b.DGNSCD22, b.DGNSCD23, b.DGNSCD24, b.DGNSCD25
   from mydata.cohort_1 as a right join seer_c.&data.0&YR as b
    on a.patient_id = b.patient_id
   where a.index_dt_new - 183 <= mdy(input(b.admsndtm, 2.), 01,
input(b.admsndty, 4.)) <= a.index_dt_new;
quit;

%end;

%mend;

%inpatient(medpar)


%macro inpatient1(data);
%do YR=10 %TO 16;

proc sql;
create table wo&YR as
  select a.patient_id, a.index_dt_new, b.admsndtm, b.admsndtd, b.admsndty,
         b.DGNSCD1, b.DGNSCD2, b.DGNSCD3, b.DGNSCD4, b.DGNSCD5,
             b.DGNSCD6, b.DGNSCD7, b.DGNSCD8, b.DGNSCD9, b.DGNSCD10,
         b.DGNSCD11, b.DGNSCD12, b.DGNSCD13, b.DGNSCD14, b.DGNSCD15,
             b.DGNSCD16, b.DGNSCD17, b.DGNSCD18, b.DGNSCD19, b.DGNSCD20,
         b.DGNSCD21, b.DGNSCD22, b.DGNSCD23, b.DGNSCD24, b.DGNSCD25
   from mydata.cohort_1 as a right join seer_c.&data&YR as b
    on a.patient_id = b.patient_id
   where a.index_dt_new - 183 <= mdy(input(b.admsndtm, 2.), 01,
input(b.admsndty, 4.)) <= a.index_dt_new;
quit;

%end;

%mend;

%inpatient1(medpar)


data medpar_c_1;
set wo:;
run;
```

```
/* NOTE: The data set WORK.MEDPAR_C_1 has 8630 observations and 30 variables.
*/

**Non-Cancer;
%macro inpatient2(data);
%do YR=6 %TO 9;

proc sql;
create table wo&YR as
  select a.patient_id, a.index_dt_new, b.admsndtm, b.admsndtd, b.admsndty,
         b.DGNSCD1, b.DGNSCD2, b.DGNSCD3, b.DGNSCD4, b.DGNSCD5,
             b.DGNSCD6, b.DGNSCD7, b.DGNSCD8, b.DGNSCD9, b.DGNSCD10,
         b.DGNSCD11, b.DGNSCD12, b.DGNSCD13, b.DGNSCD14, b.DGNSCD15,
             b.DGNSCD16, b.DGNSCD17, b.DGNSCD18, b.DGNSCD19, b.DGNSCD20,
         b.DGNSCD21, b.DGNSCD22, b.DGNSCD23, b.DGNSCD24, b.DGNSCD25
   from mydata.cohort_1 as a right join seer_nc.&data.0&YR as b
    on a.patient_id = b.patient_id
   where a.index_dt_new - 183 <= mdy(input(b.admsndtm, 2.), 01,
input(b.admsndty, 4.)) <= a.index_dt_new;
quit;

%end;

%mend;

%inpatient2(medpar)


%macro inpatient3(data);
%do YR=10 %TO 16;

proc sql;
create table wo&YR as
  select a.patient_id, a.index_dt_new, b.admsndtm, b.admsndtd, b.admsndty,
         b.DGNSCD1, b.DGNSCD2, b.DGNSCD3, b.DGNSCD4, b.DGNSCD5,
             b.DGNSCD6, b.DGNSCD7, b.DGNSCD8, b.DGNSCD9, b.DGNSCD10,
         b.DGNSCD11, b.DGNSCD12, b.DGNSCD13, b.DGNSCD14, b.DGNSCD15,
             b.DGNSCD16, b.DGNSCD17, b.DGNSCD18, b.DGNSCD19, b.DGNSCD20,
         b.DGNSCD21, b.DGNSCD22, b.DGNSCD23, b.DGNSCD24, b.DGNSCD25
   from mydata.cohort_1 as a right join seer_nc.&data&YR as b
    on a.patient_id = b.patient_id
   where a.index_dt_new - 183 <= mdy(input(b.admsndtm, 2.), 01,
input(b.admsndty, 4.)) <= a.index_dt_new;
quit;

%end;

%mend;

%inpatient3(medpar)


data medpar_nc_1;
set wo:;
run;
/* NOTE: The data set WORK.MEDPAR_NC_1 has 22820 observations and 30
variables. */
```

```
data medpar_1;
set medpar_c_1 medpar_nc_1;
run;
/*
NOTE: There were 8630 observations read from the data set WORK.MEDPAR_C_1.
NOTE: There were 22820 observations read from the data set WORK.MEDPAR_NC_1.
NOTE: The data set WORK.MEDPAR_1 has 31450 observations and 30 variables.
 */



*Outpatient claims;

 **Cancer;

%macro outpat(data);
%do YR=6 %TO 9;

proc sql;
 create table wo&YR as
  select a.patient_id, a.index_dt_new, b.from_dtm, b.from_dtd, b.from_dty,
         b.thru_dtm, b.thru_dtd, b.thru_dty,
         b.DGNS_CD1 as DGNSCD1, b.DGNS_CD2 as DGNSCD2, b.DGNS_CD3 as DGNSCD3,
b.DGNS_CD4 as DGNSCD4, b.DGNS_CD5 as DGNSCD5,
             b.DGNS_CD6 as DGNSCD6, b.DGNS_CD7 as DGNSCD7, b.DGNS_CD8 as
DGNSCD8, b.DGNS_CD9 as DGNSCD9, b.DGNS_CD10 as DGNSCD10,
         b.DGNS_CD11 as DGNSCD11, b.DGNS_CD12 as DGNSCD12, b.DGNS_CD13 as
DGNSCD13, b.DGNS_CD14 as DGNSCD14, b.DGNS_CD15 as DGNSCD15,
             b.DGNS_CD16 as DGNSCD16, b.DGNS_CD17 as DGNSCD17, b.DGNS_CD18 as
DGNSCD18, b.DGNS_CD19 as DGNSCD19, b.DGNS_CD20 as DGNSCD20,
         b.DGNS_CD21 as DGNSCD21, b.DGNS_CD22 as DGNSCD22, b.DGNS_CD23 as
DGNSCD23, b.DGNS_CD24 as DGNSCD24, b.DGNS_CD25 as DGNSCD25,
         b.hcpcs_cd
   from mydata.cohort_1 as a right join seer_c.outsaf0&YR as b
   on a.patient_id = b.patient_id
   where a.index_dt_new - 183 <= mdy(input(b.thru_dtm, 2.), 01,
input(b.thru_dty, 4.)) <= a.index_dt_new;
quit;

%end;

%mend;

%outpat(outsaf)



%macro outpatient(data);
%do YR=10 %TO 16;

proc sql;
 create table work&YR as
  select a.patient_id, a.index_dt_new, b.from_dtm, b.from_dtd, b.from_dty,
         b.thru_dtm, b.thru_dtd, b.thru_dty,
```

```
         b.DGNS_CD1 as DGNSCD1, b.DGNS_CD2 as DGNSCD2, b.DGNS_CD3 as DGNSCD3,
b.DGNS_CD4 as DGNSCD4, b.DGNS_CD5 as DGNSCD5,
             b.DGNS_CD6 as DGNSCD6, b.DGNS_CD7 as DGNSCD7, b.DGNS_CD8 as
DGNSCD8, b.DGNS_CD9 as DGNSCD9, b.DGNS_CD10 as DGNSCD10,
         b.DGNS_CD11 as DGNSCD11, b.DGNS_CD12 as DGNSCD12, b.DGNS_CD13 as
DGNSCD13, b.DGNS_CD14 as DGNSCD14, b.DGNS_CD15 as DGNSCD15,
             b.DGNS_CD16 as DGNSCD16, b.DGNS_CD17 as DGNSCD17, b.DGNS_CD18 as
DGNSCD18, b.DGNS_CD19 as DGNSCD19, b.DGNS_CD20 as DGNSCD20,
         b.DGNS_CD21 as DGNSCD21, b.DGNS_CD22 as DGNSCD22, b.DGNS_CD23 as
DGNSCD23, b.DGNS_CD24 as DGNSCD24, b.DGNS_CD25 as DGNSCD25,
         b.hcpcs_cd
   from mydata.cohort_1 as a inner join seer_c.outsaf&YR as b
   on a.patient_id = b.patient_id
   where a.index_dt_new - 183 <= mdy(input(b.thru_dtm, 2.), 01,
input(b.thru_dty, 4.)) <= a.index_dt_new;
  quit;

%end;

%mend;

%outpatient(outsaf)

data outpat_c_1;
set wo:;
run;
/* NOTE: The data set WORK.OUTPAT_C_1 has 573456 observations and 34
variables. */

**Non-cancer;
%macro outpat1(data);
%do YR=6 %TO 9;

proc sql;
 create table wo&YR as
  select a.patient_id, a.index_dt_new, b.from_dtm, b.from_dtd, b.from_dty,
         b.thru_dtm, b.thru_dtd, b.thru_dty,
         b.DGNS_CD1 as DGNSCD1, b.DGNS_CD2 as DGNSCD2, b.DGNS_CD3 as DGNSCD3,
b.DGNS_CD4 as DGNSCD4, b.DGNS_CD5 as DGNSCD5,
             b.DGNS_CD6 as DGNSCD6, b.DGNS_CD7 as DGNSCD7, b.DGNS_CD8 as
DGNSCD8, b.DGNS_CD9 as DGNSCD9, b.DGNS_CD10 as DGNSCD10,
         b.DGNS_CD11 as DGNSCD11, b.DGNS_CD12 as DGNSCD12, b.DGNS_CD13 as
DGNSCD13, b.DGNS_CD14 as DGNSCD14, b.DGNS_CD15 as DGNSCD15,
             b.DGNS_CD16 as DGNSCD16, b.DGNS_CD17 as DGNSCD17, b.DGNS_CD18 as
DGNSCD18, b.DGNS_CD19 as DGNSCD19, b.DGNS_CD20 as DGNSCD20,
         b.DGNS_CD21 as DGNSCD21, b.DGNS_CD22 as DGNSCD22, b.DGNS_CD23 as
DGNSCD23, b.DGNS_CD24 as DGNSCD24, b.DGNS_CD25 as DGNSCD25,
         b.hcpcs_cd
   from mydata.cohort_1 as a inner join seer_nc.outsaf0&YR as b
   on a.patient_id = b.patient_id
   where a.index_dt_new - 183 <= mdy(input(b.thru_dtm, 2.), 01,
input(b.thru_dty, 4.)) <= a.index_dt_new;
quit;

%end;

%mend;
```

```
%outpat1(outsaf)



%macro outpatient1(data);
%do YR=10 %TO 16;

proc sql;
 create table work&YR as
  select a.patient_id, a.index_dt_new, b.from_dtm, b.from_dtd, b.from_dty,
         b.thru_dtm, b.thru_dtd, b.thru_dty,
         b.DGNS_CD1 as DGNSCD1, b.DGNS_CD2 as DGNSCD2, b.DGNS_CD3 as DGNSCD3,
b.DGNS_CD4 as DGNSCD4, b.DGNS_CD5 as DGNSCD5,
             b.DGNS_CD6 as DGNSCD6, b.DGNS_CD7 as DGNSCD7, b.DGNS_CD8 as
DGNSCD8, b.DGNS_CD9 as DGNSCD9, b.DGNS_CD10 as DGNSCD10,
         b.DGNS_CD11 as DGNSCD11, b.DGNS_CD12 as DGNSCD12, b.DGNS_CD13 as
DGNSCD13, b.DGNS_CD14 as DGNSCD14, b.DGNS_CD15 as DGNSCD15,
             b.DGNS_CD16 as DGNSCD16, b.DGNS_CD17 as DGNSCD17, b.DGNS_CD18 as
DGNSCD18, b.DGNS_CD19 as DGNSCD19, b.DGNS_CD20 as DGNSCD20,
         b.DGNS_CD21 as DGNSCD21, b.DGNS_CD22 as DGNSCD22, b.DGNS_CD23 as
DGNSCD23, b.DGNS_CD24 as DGNSCD24, b.DGNS_CD25 as DGNSCD25,
         b.hcpcs_cd
   from mydata.cohort_1 as a inner join seer_nc.outsaf&YR as b
   on a.patient_id = b.patient_id
   where a.index_dt_new - 183 <= mdy(input(b.thru_dtm, 2.), 01,
input(b.thru_dty, 4.)) <= a.index_dt_new;
  quit;

%end;

%mend;

%outpatient1(outsaf)

data outpat_nc_1;
set wo:;
run;
/* NOTE: The data set WORK.OUTPAT_NC_1 has 1193505 observations and 34
variables. */

data outpat_1;
set outpat_c_1 outpat_nc_1;
run;
/*
NOTE: There were 573456 observations read from the data set WORK.OUTPAT_C_1.
NOTE: There were 1193505 observations read from the data set
WORK.OUTPAT_NC_1.
NOTE: The data set WORK.OUTPAT_1 has 1766961 observations and 34 variables.
*/

*Carrier claims;

 **Cancer;

%macro carrier(data);
%do YR=6 %TO 9;
```

```
proc sql;
 create table wo&YR as
  select a.patient_id, a.index_dt_new, b.from_dtm, b.from_dtd, b.from_dty,
         b.thru_dtm, b.thru_dtd, b.thru_dty,
         b.DGNS_CD1 as DGNSCD1, b.DGNS_CD2 as DGNSCD2, b.DGNS_CD3 as DGNSCD3,
b.DGNS_CD4 as DGNSCD4, b.DGNS_CD5 as DGNSCD5,
             b.DGNS_CD6 as DGNSCD6, b.DGNS_CD7 as DGNSCD7, b.DGNS_CD8 as
DGNSCD8, b.DGNS_CD9 as DGNSCD9, b.DGNS_CD10 as DGNSCD10,
         b.DGNS_CD11 as DGNSCD11, b.DGNS_CD12 as DGNSCD12,
         b.hcpcs_cd
   from mydata.cohort_1 as a inner join seer_c.&data.0&YR as b
   on a.patient_id = b.patient_id
   where a.index_dt_new - 183 <= mdy(input(b.thru_dtm, 2.), 01,
input(b.thru_dty, 4.)) <= a.index_dt_new;
quit;

%end;

%mend;

%carrier(nch)



%macro carrier1(data);
%do YR=10 %TO 16;

proc sql;
 create table work&YR as
  select a.patient_id, a.index_dt_new, b.from_dtm, b.from_dtd, b.from_dty,
         b.thru_dtm, b.thru_dtd, b.thru_dty,
         b.DGNS_CD1 as DGNSCD1, b.DGNS_CD2 as DGNSCD2, b.DGNS_CD3 as DGNSCD3,
b.DGNS_CD4 as DGNSCD4, b.DGNS_CD5 as DGNSCD5,
             b.DGNS_CD6 as DGNSCD6, b.DGNS_CD7 as DGNSCD7, b.DGNS_CD8 as
DGNSCD8, b.DGNS_CD9 as DGNSCD9, b.DGNS_CD10 as DGNSCD10,
         b.DGNS_CD11 as DGNSCD11, b.DGNS_CD12 as DGNSCD12,
         b.hcpcs_cd
   from mydata.cohort_1 as a inner join seer_c.&data&YR as b
   on a.patient_id = b.patient_id
   where a.index_dt_new - 183 <= mdy(input(b.thru_dtm, 2.), 01,
input(b.thru_dty, 4.)) <= a.index_dt_new;
  quit;

%end;

%mend;

%carrier1(nch)

data nch_c_1;
set wo:;
run;
/* NOTE: The data set WORK.NCH_C_1 has 1369812 observations and 21 variables.
*/

**Non-cancer;
```

```
%macro carrier2(data);
%do YR=6 %TO 9;

proc sql;
 create table wo&YR as
  select a.patient_id, a.index_dt_new, b.from_dtm, b.from_dtd, b.from_dty,
         b.thru_dtm, b.thru_dtd, b.thru_dty,
         b.DGNS_CD1 as DGNSCD1, b.DGNS_CD2 as DGNSCD2, b.DGNS_CD3 as DGNSCD3,
b.DGNS_CD4 as DGNSCD4, b.DGNS_CD5 as DGNSCD5,
             b.DGNS_CD6 as DGNSCD6, b.DGNS_CD7 as DGNSCD7, b.DGNS_CD8 as
DGNSCD8, b.DGNS_CD9 as DGNSCD9, b.DGNS_CD10 as DGNSCD10,
         b.DGNS_CD11 as DGNSCD11, b.DGNS_CD12 as DGNSCD12,
         b.hcpcs_cd
   from mydata.cohort_1 as a inner join seer_nc.&data.0&YR as b
   on a.patient_id = b.patient_id
   where a.index_dt_new - 183 <= mdy(input(b.thru_dtm, 2.), 01,
input(b.thru_dty, 4.)) <= a.index_dt_new;
quit;

%end;

%mend;

%carrier2(nch)



%macro carrier3(data);
%do YR=10 %TO 16;

proc sql;
 create table work&YR as
  select a.patient_id, a.index_dt_new, b.from_dtd, b.from_dty,
         b.thru_dtm, b.thru_dtd, b.thru_dty,
         b.DGNS_CD1 as DGNSCD1, b.DGNS_CD2 as DGNSCD2, b.DGNS_CD3 as DGNSCD3,
b.DGNS_CD4 as DGNSCD4, b.DGNS_CD5 as DGNSCD5,
             b.DGNS_CD6 as DGNSCD6, b.DGNS_CD7 as DGNSCD7, b.DGNS_CD8 as
DGNSCD8, b.DGNS_CD9 as DGNSCD9, b.DGNS_CD10 as DGNSCD10,
         b.DGNS_CD11 as DGNSCD11, b.DGNS_CD12 as DGNSCD12,
         b.hcpcs_cd
   from mydata.cohort_1 as a inner join seer_nc.&data&YR as b
   on a.patient_id = b.patient_id
   where a.index_dt_new - 183 <= mdy(input(b.thru_dtm, 2.), 01,
input(b.thru_dty, 4.)) <= a.index_dt_new;
  quit;

%end;

%mend;

%carrier3(nch)

data carrier_nc_1;
set wo:;
run;
```

```
/* NOTE: The data set WORK.CARRIER_NC_1 has 2668405 observations and 21
variables. */

data carrier_1;
set Nch_c_1 carrier_nc_1;
run;
/*
NOTE: There were 1369812 observations read from the data set WORK.NCH_C_1.
NOTE: There were 2668405 observations read from the data set
WORK.CARRIER_NC_1.
NOTE: The data set WORK.CARRIER_1 has 4038217 observations and 21 variables.
*/

data claims_1;

set medpar_1(keep=patient_id index_dt_new admsndtm admsndtd admsndty dgnscd1-
dgnscd25)
    outpat_1
    carrier_1;

if admsndtm ne . then claim_dt=mdy(admsndtm,admsndtd,admsndty);

if thru_dtm ne . then claim_dt=mdy(thru_dtm, thru_dtd, thru_dty);

format claim_dt mmddyy10.;

run;
/* NOTE: The data set WORK.CLAIMS_1 has 5836628 observations and 38
variables. */

data mydata.claims_1; set claims_1; run;

data cohort_claims_1;

set claims_1;

hyperlipidemia=0; hypogonadism = 0; hypertension=0; wasting =0; malaise=0;
osteoporosis =0; depression= 0;
pituitary_dysf=0; diabetes = 0; cardiovascular=0;
breast_test=0; colorectal_test=0; ovarian_test=0; PSA_test=0;

array diag(*) dgnscd1-dgnscd25;

do i=1 to dim(diag);

if diag(i) in ('2722' '2724') then hyperlipidemia = 1;

if diag(i) in ('2572') then hypogonadism = 1;

if diag(i) in ('4011' '4019' '40210' '40290' '40410' '40490' '4051' '4059')
then hypertension=1;

if diag(i) in ('7292') then wasting=1;

if diag(i) in ('78071' '78072' '78079') then malaise=1;

if diag(i) in ('73300' '73301' '73302' '73303' '73309') then osteoporosis=1;
```

```
if diag(i) in ('311') then depression=1;

if diag(i) in ('1943' '2273' '2534') then pituitary_dysf=1;

if diag(i) in ('25000' '25002' '25010' '25012' '25020' '25022' '25030'
'25032' '25040' '25042' '25050' '25052' '25060' '25062' '25070' '25072'
'25080'
'25082' '25090' '25092')
then diabetes = 1;

if  substr(diag(i),1,3) IN ('410','411','412','413','414') or
    substr(diag(i),1,4) IN ('0066') or
    substr(diag(i),1,5) IN ('V4581','V4582','0066') or
    substr(diag(i),1,2) IN ('36') or
    substr(diag(i),1,3) IN ('430','431','432','433','434','435','381')  or
    substr(diag(i),1,4) IN ('0061','0062','0062','0063','0064','0065','3974')
or
    substr(diag(i),1,3) IN ('425','428') or
    substr(diag(i),1,4) IN ('V421','3751') or
    substr(diag(i),1,5) IN ('44389') or
    substr(diag(i),1,4) IN ('4439') or
    substr(diag(i),1,3) IN ('410') or
    substr(diag(i),1,3) IN('410','411','412','413','414') then
cardiovascular= 1;


end;


**Breast test;
if hcpcs_cd in ('G0202' 'G0204' 'G0206' '76092' '76090' '76091') then
breast_test=1;
**Colorectal test;
if hcpcs_cd in ('45378' '45379' '45380' '45381' '45382' '45388' '45384'
'45385' '45386' '45389' '45391' '45392' '45390' '45393' '45398' 'G0105'
'G0121') then colorectal_test=1;
**Ovarian test;
if hcpcs_cd in ('76830' '76856' '76857') then ovarian_test=1;
**Prostate cancer test;
if hcpcs_cd in ('84153' 'G0103') then PSA_test=1;

/*if hyperlipidemia=1 or hypogonadism=1 or  hypertension=1 or wasting=1 or
malaise=1 or osteoporosis=1 or depression=1 or pituitary_dysf=1 or diabetes=1
or cardiovascular=1;*/
drop i;

run;
/* NOTE: The data set WORK.COHORT_CLAIMS_1 has 5836628 observations and 52
variables. */

proc freq data=cohort_claims_1;
table
hyperlipidemia
hypogonadism
hypertension
wasting
```

```
malaise
osteoporosis
depression
pituitary_dysf
diabetes
cardiovascular
breast_test
colorectal_test
ovarian_test
PSA_test;
run;


/* Create 2 datasets for claims and another one tests */

**Claims data;
data cohort_claims2_1;
set cohort_claims_1;
where hyperlipidemia=1 or hypogonadism=1 or  hypertension=1 or wasting=1 or
malaise=1 or osteoporosis=1 or depression=1 or pituitary_dysf=1 or diabetes=1
or cardiovascular=1;
run;
/* NOTE: The data set WORK.COHORT_CLAIMS2_1 has 2406202 observations and 52
variables. */

proc sort data=cohort_claims2_1;
by patient_id claim_dt;
run;

proc sql;
select count (distinct patient_id) from cohort_claims2_1; quit;
/* n=116451  */

proc sql;
 create table cohort_claims_pat_1 as
  select patient_id, max(diabetes) as diabetes, max (hypogonadism) as
hypogonadism, max(hypertension) as hypertension,
         max(wasting) as wasting, max(malaise) as malaise, max(osteoporosis)
as osteoporosis,
         max(pituitary_dysf) as pituitary_dysf, max(depression) as
depression, max( hyperlipidemia) as  hyperlipidemia,
             max(cardiovascular) as cardiovascular
   from cohort_claims2_1
   group by patient_id
;quit;
/* NOTE: Table WORK.COHORT_CLAIMS_PAT_1 created, with 116451 rows and 11
columns. */

*Outpatient visits;
proc sql;
 create table cohort_visits_1 as
  select patient_id, count(distinct claim_dt) as visits_no
   from claims_1
   group by patient_id
;quit;
/* NOTE: Table WORK.COHORT_VISITS_1 created, with 130919 rows and 2 columns.
*/
```

```
**Tests data;

data breast_test_1;
set cohort_claims_1;
where breast_test=1;
run;
/* NOTE: The data set WORK.BREAST_TEST_1 has 44510 observations and 52
variables. */

proc sql;
select count (distinct patient_id) from breast_test_1; quit;
/* n=25828 */

proc sql;
 create table breast_test1_1 as
  select patient_id, count (distinct claim_dt) as breast_test_no
   from breast_test_1
   group by patient_id
;quit;
/* NOTE: Table WORK.BREAST_TEST1_1 created, with 25828 rows and 2 columns. */

data col_test_1;
set cohort_claims_1;
where colorectal_test=1;
run;
/* NOTE: The data set WORK.COL_TEST_1 has 14253 observations and 52
variables. */

proc sql;
select count (distinct patient_id) from col_test_1; quit;
/* n=6684 */

proc sql;
 create table col_test1_1 as
  select patient_id, count (distinct claim_dt) as col_test_no
   from col_test_1
   group by patient_id
;quit;
/* NOTE: Table WORK.COL_TEST1_1 created, with 6684 rows and 2 columns. */

data ov_test_1;
set cohort_claims_1;
where ovarian_test=1;
run;
/* NOTE: The data set WORK.OV_TEST_1 has 5998 observations and 52 variables.
*/

proc sql;
select count (distinct patient_id) from ov_test_1; quit;
/* n=2935 */

proc sql;
 create table ov_test1_1 as
  select patient_id, count (distinct claim_dt) as ov_test_no
   from ov_test_1
```

```
   group by patient_id
;quit;
/* NOTE: Table WORK.OV_TEST1_1 created, with 2935 rows and 2 columns. */


data psa_test_1;
set cohort_claims_1;
where psa_test=1;
run;
/* NOTE: The data set WORK.PSA_TEST_1 has 23 observations and 52 variables.
*/

proc sql;
select count (distinct patient_id) from psa_test_1; quit;
/* n=21  */

proc sql;
 create table psa_test1_1 as
  select patient_id, count (distinct claim_dt) as psa_test_no
   from psa_test_1
   group by patient_id
;quit;
/* NOTE: Table WORK.PSA_TEST1_1 created, with 21 rows and 2 columns. */




/*merge with cohort*/

proc sql;
 create table mydata.cohort1_1 as
  select A.*, B.*, C.visits_no, D.breast_test_no, E.col_test_no,
F.ov_test_no, G.psa_test_no
   from mydata.cohort_1 as A
    left join cohort_claims_pat_1 as B
     on A.patient_id = B.patient_id
    left join cohort_visits_1 as C
     on A.patient_id = C.patient_id
      left join breast_test1_1 as D
       on A.patient_id = D.patient_id
    left join col_test1_1 as E
       on A.patient_id = E.patient_id
      left join ov_test1_1 as F
       on A.patient_id = F.patient_id
    left join psa_test1_1 as G
     on A.patient_id = G.patient_id
;quit;
/* NOTE: Table MYDATA.COHORT1_1 created, with 142772 rows and 75 columns. */

data mydata.cohort1_1;
set mydata.cohort1_1;

array variable(*) bca cca oca eca cancer Testosterone T_sup Statin S_sup
both_drugs supply_days diabetes hypogonadism hypertension wasting malaise
osteoporosis pituitary_dysf
             depression hyperlipidemia cardiovascular visits_no
breast_test_no col_test_no ov_test_no psa_test_no;
```

```
do i=1 to dim(variable);
if variable(i)=. then variable(i)=0;
end;
drop i;
run;
/* NOTE: The data set MYDATA.COHORT1_1 has 142772 observations and 75
variables. */


proc freq data=mydata.cohort1_1;
table bca cca oca eca cancer Testosterone Statin both_drugs diabetes
hypogonadism hypertension wasting malaise
      osteoporosis pituitary_dysf
      depression hyperlipidemia cardiovascular visits_no breast_test_no
col_test_no ov_test_no psa_test_no;
run;

proc freq data=mydata.cohort1_1;
where exposed=1;
table Testosterone Statin both_drugs; /* OK */
run;

proc freq data=mydata.cohort1_1;
where exposed=0;
table Testosterone Statin both_drugs; /* Blank-all are equal to zero, OK */
run;



/* Add insulin use variable within 6 months of index date */

/*List relevant NDC codes*/
proc sql;
create table tmp as
select ndc,theraClassDesc, productname, theraclasscode, roacode,
masterformcode
from data.mastertheraclass
where upcase(theraClassDesc) like '%INSULIN%'
;quit; /*295 rows*/

/*Create format with the NDC codes*/
data myformatDat;
 retain fmtname 'insNDC' type 'c' start label;
set tmp(keep = ndc rename = (ndc = start)) end = eof;
 output;
 if eof then
 do;
   start = ' ';
   label = 'NO';
   HLO = 'O';
   output;
 end;
run;
/* NOTE: The data set WORK.MYFORMATDAT has 296 observations and 5 variables.
*/
```

```
proc format cntlin = myformatDat; run; /*n=296*/

/*Insulin from cancer and non cancer pdesf files */

data ins_claims16;
set seer_c.pdesaf:;
where put(prod_srvc_id, $insNDC.) ^= 'NO';
run;
/* NOTE: The data set WORK.INS_CLAIMS16 has 2343205 observations and 21
variables. */


proc sql;
select count (distinct prod_srvc_id) from ins_claims16;
quit; /*99 (total ndc insulin count)*/

proc sql;
select count (distinct patient_id) from ins_claims16 where patient_id IN
(select patient_id from mydata.cohort1_1);
quit; /*8398  women with cancer in cohort who were prescribed with insulin
ndc. */

data ins_claims_sum;
set seer_nc.pdesaf:;
where put(prod_srvc_id, $insNDC.) ^= 'NO';
run;
/* NOTE: The data set WORK.INS_CLAIMS_SUM has 2070199 observations and 21
variables. */

proc sql;
select count (distinct prod_srvc_id) from ins_claims_sum ;
quit; /* 98 (total ndc statin count) */

proc sql;
select count (distinct patient_id) from ins_claims_sum where patient_id IN
(select patient_id from mydata.cohort1_1);
quit; /*5568  women with no cancer in cohort who were prescribed with insulin
ndc. */


/*Insulin injection from cancer and non-cancer claims */

data mydata.ins_inj16;
set seer_c.nch: (keep=patient_id from_dtm from_dtd from_dty hcpcs_cd)
    seer_c.outsaf:(keep=patient_id from_dtm from_dtd from_dty hcpcs_cd);
where hcpcs_cd IN ('J1815');
run; /* NOTE: The data set MYDATA.INS_INJ16 has 43650 observations and 5
variables. */

/*Extract testosterone or statin injections data from non-cancer patients*/
data mydata.ins_inj_non;
set seer_nc.nch: (keep=patient_id from_dtm from_dtd from_dty hcpcs_cd)
    seer_nc.outsaf:(keep=patient_id from_dtm from_dtd from_dty hcpcs_cd);
where hcpcs_cd IN ('J1815');
run; /* NOTE: The data set MYDATA.INS_INJ_NON has 33144 observations and 5
variables. */
```

```
/*All insulin claims*/
proc sql;
 create table insulin as
  select patient_id, srvc_mon, srvc_day, srvc_yr, days_suply_num
   from ins_claims16 /* From ndc insulin with cancer */
 UNION ALL
   select patient_id, srvc_mon, srvc_day, srvc_yr, days_suply_num
   from ins_claims_sum /* From ndc insulin without cancer */
 UNION ALL
   select patient_id, from_dtm as srvc_mon, from_dtd as srvc_day, from_dty as
srvc_yr, . as days_suply_num /*injection is always 'one' time, thus days of
supply is missing*/
   from mydata.ins_inj16 /* From CPT/injection insulin with cancer */
 UNION ALL
   select patient_id, from_dtm as srvc_mon, from_dtd as srvc_day, from_dty as
srvc_yr, . as days_suply_num
   from mydata.ins_inj_non;  /* From CPT/injection statin without cancer */
quit;
/* NOTE: Table WORK.INSULIN created, with 4490198 rows and 5 columns. */

data mydata.insulin;
set insulin;
run;

data insulin;
set mydata.insulin;
run;
/* NOTE: The data set WORK.INSULIN has 4490198 observations and 5 variables.
*/


proc freq data=insulin;
table days_suply_num;
run;
/* OK */



data insulin;
set insulin;
fill_dt = mdy(srvc_mon, srvc_day, srvc_yr);
ins_fill_dt=mdy(month(fill_dt),'01', year(fill_dt));
format fill_dt mmddyy10.;
format ins_fill_dt mmddyy10.;
run;
/* NOTE: The data set WORK.INSULIN has 4490198 observations and 7 variables.
*/

/* Select insulin services from 6 months before index date */
proc sort data=insulin;
by patient_id ins_fill_dt;
run;

proc sql;
create table insulin1
```

```
as select B.*, A.index_dt_new from mydata.cohort1_1 as A right join insulin
as B
on B.patient_id=A.patient_id
where index_dt_new-183 <= ins_fill_dt <= index_dt_new;
quit;
/* NOTE: Table WORK.INSULIN1 created, with 19735 rows and 8 columns. */

proc sort data=insulin1 nodupkey out=insulin2;
by patient_id;
run;
/* NOTE: The data set WORK.INSULIN2 has 5044 observations and 8 variables. */

data insulin2;
set insulin2;
insulin=1;
run;
/* NOTE: The data set WORK.INSULIN2 has 5044 observations and 9 variables. */

data mydata.cohort2_1;
merge mydata.cohort1_1 insulin2(keep=patient_id insulin);
by patient_id;
if insulin=. then insulin=0;
run;
/* NOTE: The data set MYDATA.COHORT2_1 has 142772 observations and 76
variables. */

proc freq data=mydata.cohort2_1;
table insulin diabetes insulin*diabetes;
run;/*5044 with insulin use last 6 months*/

/* Extract Charlson's comorbidity index */


%*********************************************************************;
%*  Date: February 2006                                              *;
%*                                                                   *;
%*  This code is presented on the MCHP web site with permission from *;
%*  Hude Quan.  The code was used in a study titled, "Coding algorithms *;
%*  for defining comorbidities in ICD-9-CM and ICD-10 administrative *;
%*  data." in Med Care, 43(11):1130-9.                               *;
%*                                                                   *;
%*  NOTE:  This code has not been validated by MCHP.                 *;
%*********************************************************************;
libname mydata 'Z:\Projects\Digbeu-B\Testosterone plus statins w CVDs among
cancer survivors women\Mydata';
%macro ICD9_E_CH(library=, dataset=, OUTPUT=);
data &library..&OUTPUT;
      set &library..&dataset (keep=patient_id DGNSCD1-DGNSCD25);

      /*DC: Disease Codes*/
      %LET DC1=%STR('410','412');
      %LET
DC2=%STR('39891','40201','40211','40291','40401','40403','40411','40413','404
91','40493','4254','4255','4257','4258','4259','428');
      %LET
DC3=%STR('0930','4373','440','441','4431','4432','4438','4439','4471','5571',
'5579','V434');
```

```
	%LET
DC4=%STR('36234','430','431','432','433','434','435','436','437','438');
	%LET DC5=%STR('290','2941','3312');
	%LET
DC6=%STR('4168','4169','490','491','492','493','494','495','496','500','501',
'502','503','504','505','5064','5081','5088');
	%LET
DC7=%STR('4465','7100','7101','7102','7103','7104','7140','7141','7142','7148
','725');
	%LET DC8=%STR('531','532','533','534');
	%LET
DC9=%STR('07022','07023','07032','07033','07044','07054','0706','0709','570',
'571','5733','5734','5738','5739','V427');
	%LET DC10=%STR('2500','2501','2502','2503','2508','2509');
	%LET DC11=%STR('2504','2505','2506','2507');
	%LET
DC12=%STR('3341','342','343','3440','3441','3442','3443','3444','3445','3446'
,'3449');
	%LET
DC13=%STR('40301','40311','40391','40402','40403','40412','40413','40492','40
493','582','5830','5831','5832','5834','5836','5837','585','586','5880','V420
','V451','V56');
	%LET
DC14=%STR('140','141','142','143','144','145','146','147','148','149','150','
151','152','153','154','155','156','157','158','159','160','161','162','163',
'164','165','170','171','172','174','175','176','179','180','181','182','183'
,'184','185','186','187','188','189','190','191','192','193','194','195','200
','201','202','203','204','205','206','207','208','2386');
	%LET DC15=%STR('4560','4561','4562','5722','5723','5724','5728');
	%LET DC16=%STR('196','197','198','199');
	%LET DC17=%STR('042','043','044');
	/**Myocardial Infarction**/
	%LET DIS1=MI;
	%LET LBL1=%STR(Myocardial Infarction);

	/**Congestive Heart Failure**/
	%LET DIS2=CHF;
	%LET LBL2=%STR(Congestive Heart Failure);

	/**Periphral Vascular Disease**/
	%LET DIS3=PVD;
	%LET LBL3=%STR(Periphral Vascular Disease);

	/**Cerebrovascular Disease**/
	%LET DIS4=CEVD;
	%LET LBL4=%STR(Cerebrovascular Disease);

	/**Dementia**/
	%LET DIS5=DEM;
	%LET LBL5=%STR(Dementia);

	/*Chronic Pulmonary Disease*/
	%LET DIS6=COPD;
	%LET LBL6=%STR(Chronic Pulmonary Disease);

	/**Connective Tissue Disease-Rheumatic Disease**/
	%LET DIS7=Rheum;
```

```
       %LET LBL7=%STR(Connective Tissue Disease-Rheumatic Disease);

       /**Peptic Ulcer Disease**/
       %LET DIS8=PUD;
       %LET LBL8=%STR(Peptic Ulcer Disease);

       /**Mild Liver Disease **/
       %LET DIS9=MILDLD;
       %LET LBL9=%STR(Mild Liver Disease);

       /**Diabetes without complications**/
       %LET DIS10=DIAB_NC;
       %LET LBL10=%STR(Diabetes without complications);

       /**Diabetes with complications**/
       %LET DIS11=DIAB_C;
       %LET LBL11=%STR(Diabetes with complications);

       /**Paraplegia and Hemiplegia**/
       %LET DIS12=PARA;
       %LET LBL12=%STR(Paraplegia and Hemiplegia);

       /**Renal Disease**/
       %LET DIS13=RD;
       %LET LBL13=%STR(Renal Disease);

       /**Cancer**/
       %LET DIS14=CANCER;
       %LET LBL14=%STR(Cancer);

       /**Moderate or Severe Liver Disease**/
       %LET DIS15=MSLD;
       %LET LBL15=%STR(Moderate or Severe Liver Disease);

       /**Metastatic Carcinoma **/
       %LET DIS16=METS;
       %LET LBL16=%STR(Metastatic Carcinoma);

       /**AIDS/HIV**/
       %LET DIS17=HIV;
       %LET LBL17=%STR(AIDS/HIV);

       %do DI=1 %to 17;/*ICD9-E Charlson: 17 groups*/
             A&DI=0;
             %do DX=1 %to 25;  /*DX_1 - DX_16*/
                   B&DX=0;
                   %do SN=3 %to 5;
                         if substr(DGNSCD&DX,1,&SN) in (&&DC&DI) then
     C&SN=1;else C&SN=0;
                         B&DX=B&DX +C&SN;
                         drop C&SN;
                   %end;
                   A&DI=A&DI+B&DX;
                   DROP B&DX;
             %end;
             if A&DI>0 then    ICD9_E_CH_&&DIS&DI=1;else ICD9_E_CH_&&DIS&DI=0;
             label ICD9_E_CH_&&DIS&DI = &&LBL&DI;
```

```
            DROP A&DI;
      %end;

run;
%mend ICD9_E_CH;
%ICD9_E_CH(library=mydata, dataset=claims_1, OUTPUT=comorb_1);

/* NOTE: The data set MYDATA.COMORB_1 has 5836628 observations and 43
variables. */

proc contents varnum data=mydata.comorb_1;
run;

proc sql;

create table mydata.charlson_person_1 as

select patient_id,

            max(  ICD9_E_CH_MI  ) as  MI      ,

            max(  ICD9_E_CH_CHF    ) as CHF ,

            max(  ICD9_E_CH_PVD      ) as  PVD   ,

            max(  ICD9_E_CH_CEVD      ) as  CEVD   ,

            max(  ICD9_E_CH_DEM     ) as  DEM  ,

            max(  ICD9_E_CH_COPD     ) as  COPD  ,

            max(  ICD9_E_CH_Rheum ) as  RHEUM    ,

            max(  ICD9_E_CH_PUD     ) as  PUD   ,

            max(  ICD9_E_CH_MILDLD   ) as  MILDLD      ,

            max(  ICD9_E_CH_DIAB_NC  ) as  DIAB_NC      ,

            max(  ICD9_E_CH_DIAB_C     ) as  DIAB_C  ,

            max(  ICD9_E_CH_PARA    ) as  PARA ,

            max(  ICD9_E_CH_RD      ) as  RD   ,

            max(  ICD9_E_CH_CANCER   ) as  CANCER      ,

            max(  ICD9_E_CH_MSLD  ) as  MSLD      ,

            max(  ICD9_E_CH_METS  ) as  METS      ,

            max(  ICD9_E_CH_HIV ) as  HIV

      from mydata.comorb_1

      group by patient_id;
```

```
quit;

/* NOTE: Table MYDATA.CHARLSON_PERSON_1 created, with 130919 rows and 18
columns. */



data mydata.charlson_person_1;
set mydata.charlson_person_1;
if DIAB_NC=1 or DIAB_C=1 then DIAB=1; else DIAB=0;
if MILDLD=1 OR MSLD=1 then LD=1; else LD=0;
**Create Charlson comorbidity index category;
cci=sum(of  MI CHF PVD CEVD DEM COPD RHEUM PUD LD PARA RD HIV); **Exclude
DIAB, METS and CANCER;
run;
/* NOTE: The data set MYDATA.CHARLSON_PERSON_1 has 130919 observations and 21
variables. */

proc freq data=mydata.charlson_person_1;
table MI CHF PVD CEVD DEM COPD RHEUM PUD LD DIAB PARA RD CANCER METS HIV cci;
run;



* Merge with cohort;
proc sql;
create table mydata.cohort3_1
as select B.*, A.cci from mydata.charlson_person_1 as A right join
mydata.cohort2_1 as B
on B.patient_ID=A.patient_id;
quit;
/* NOTE: Table MYDATA.COHORT3_1 created, with 142772 rows and 77 columns. */

data mydata.cohort3_1;
set mydata.cohort3_1;
if cci=. then cci=0;
if cci=0 then cci_cat=0;
else if cci=1 then cci_cat=1;
else if cci=2 then cci_cat=2;
else cci_cat=3;
run;
/* NOTE: The data set MYDATA.COHORT3_1 has 142772 observations and 78
variables. */




proc freq data=mydata.cohort3_1;
table cci_cat;
run;


/*Add variables from census tract: Poverty level and years of education
Percent of adults below poverty line and without high school or above grade.
Have to import these variables frem previous saved datasets. Keep the
zip_code at indexdt year
for each patient. Then merge with zip census data*/
```

```
proc contents data=mydata.cases1;
run;
proc contents varnum data=data.zipcode;
run;
proc contents varnum data=data.tract;
run;

/* Extract zip code for parients in your cohort */

data zip_a(keep=patient_id zip5: state:);
set mydata.cases1 mydata.control1;
run;
/* NOTE: The data set WORK.ZIP_A has 576565 observations and 65 variables. */

data zip_b;
merge zip_a(in=a) mydata.cohort3_1(in=b);
by patient_id;
if a and b;
run;
/* NOTE: The data set WORK.ZIP_B has 142772 observations and 142 variables.
*/


data zip_c;
set zip_b;
array temp[*] zip5_2007- zip5_2015;
array temp1[*] state2007-state2015;
zip = temp[year(index_dt_new) - 2006];
state = temp1[year(index_dt_new) - 2006];
drop zip5_1991- zip5_2017 state1991-state2017 statecd1-statecd10;
run;
/* NOTE: The data set WORK.ZIP_C has 142772 observations and 80 variables. */


*Merge with SEER zipcode dataset;
proc sql;
 create table mydata.cohort_final_1_A as
  select a.*, b.zpnon as education, b.pov_tot as poverty
   from zip_c as a
    left join data.zipcode as b
     on a.zip = b.zip5
     and a.state = b.state
     and b.filetype = '02' /*from latest census: 2000*/
;quit;
/* NOTE: Table MYDATA.COHORT_FINAL_1_A created, with 142772 rows and 82
columns. */


/* Create death variable:
   FYI: For cancer diagnosed patients, mortality is based on cancer-related
mortality and for non-cancer patients, mortality is based on Medicare death
date */
proc contents varnum data=mydata.cohort_final_1_A;
run;
```

```
proc freq data=mydata.cohort_final_1_A;
tables year_index_dt_new*med_dody dx_date;
run;


proc freq data=mydata.cohort_final_1_A;
tables med_dody vsrtdx1 med_dody*vsrtdx1;
run;

data mydata.cohort_final_1_A;
set mydata.cohort_final_1_A;

death=0;

if cancer_diagnosed=1 then do;
if vsrtdx1=1 and 2007 <= med_dody <= 2016 then death=1;
end;

if cancer_diagnosed=0 then do;
if 2007 <= med_dody <= 2016 then death=1;
end;

run;
/* NOTE: The data set MYDATA.COHORT_FINAL_1_A has 142772 observations and 83
variables. */

proc freq data=mydata.cohort_final_1_A;
tables death;
run;
/*
death   Frequency    Percent    Cumulative Frequency     Cumulative Percent
    0      119092      83.41            119092                     83.41
    1       23680      16.59            142772                    100.00
*/

proc freq data=mydata.cohort_final_1_A;
where cancer_diagnosed=1;
tables death;
run;
/*
death   Frequency    Percent    Cumulative Frequency     Cumulative Percent
    0      44612       85.65            44612                      85.65
    1      7474        14.35            52086                     100.00
*/

proc freq data=mydata.cohort_final_1_A;
where cancer_diagnosed=0;
tables death;
run;
/*
death   Frequency    Percent    Cumulative Frequency     Cumulative Percent
    0       74480      82.13            74480                      82.13
    1       16206      17.87            90686                     100.00
*/

proc freq data=mydata.cohort_final_1_A;
where exposed=1;
```

```
tables death;
run;


proc freq data=mydata.cohort_final_1_A;
where exposed=0;
tables death;
run;



/* Create drug category variable */
data mydata.cohort_final_1_A;
set mydata.cohort_final_1_A;

drug_cat=0; *NONE/UNEXPOSED;

if exposed=1 and both_drugs=0 then do;
if statin=1 then drug_cat=1; *STATIN ONLY;
if testosterone=1 then drug_cat=2; *TTH ONLY;
end;

if exposed=1 and both_drugs=1 then drug_cat=3; *BOTH DRUGS;

run;
/* NOTE: The data set MYDATA.COHORT_FINAL_1_A has 142772 observations and 84
variables. */



proc freq data=mydata.cohort_final_1_A;
tables testosterone statin both_drugs;
table both_drugs*(testosterone statin);
table drug_cat;
run;
/* OK */

proc freq data=mydata.cohort_final_1_A;
tables drug_cat*exposed;
run;

proc freq data=mydata.cohort_final_1_A;
where drug_cat > 0;
tables statin testosterone both_drugs;
run;
/* OK */


/* Create age and race category, and redefine cancer characteristics*/
proc freq data=mydata.cohort_final_1_A;
table agedx1 age_exposed advanced_cancer_stage high_tumor_grade;
run;

data mydata.cohort_final_1_A;
set mydata.cohort_final_1_A;
if exposed =0 then do;
age_exposed=2015-birthyr;
```

```
end;
if 65 <= age_exposed <= 69 then age_cat='1';
else if 70 <= age_exposed <= 74 then age_cat='2';
else if 75 <= age_exposed <= 79 then age_cat='3';
else age_cat='4';

if race='1' then white='1'; else white='0';
if race='2' then black='1'; else black='0';
if race='5' then hisp='1'; else hisp='0';
if race in ('0' '3' '4' '6' '') then other='1'; else other='0';

if advanced_cancer_stage=. then advanced_cancer_stage=2;
if high_tumor_grade=. then high_tumor_grade=2;
run;
/* NOTE: The data set MYDATA.COHORT_FINAL_1_A has 142772 observations and 89
variables. */


data mydata.cohort_final_1_A;
set mydata.cohort_final_1_A;

if race='1' then race_cat='0';
if race='2' then race_cat='1';
if race='5' then race_cat='2';
if race in ('0' '3' '4' '6' '') then race_cat='3';

run;

/* NOTE: The data set MYDATA.COHORT_FINAL_1_A has 142772 observations and 90
variables. */



proc freq data=mydata.cohort_final_1_A;
table age_exposed age_cat race white black hisp other race_cat
advanced_cancer_stage high_tumor_grade;
run;
/* OK */





proc freq data=mydata.cohort_final_1_A;
table death*drug_cat/chisq;
where cancer_diagnosed='1';
run;
/* OK */
```

```
**Exclude prevalent users of stain and TTH in the year prior index date, this
will imply to select drug exposure date
  from Janary 2008 to June 2015 (Exclude from drug exposure from July 2007 to
December 2007);


proc contents data=mydata.cohort_final_1_A; run;

proc freq data=mydata.cohort_final_1_A; table index_dt_new; run;

proc freq data=mydata.cohort_final_1_A; table dx_date; run;

proc sort data=mydata.cohort_final_1_A;
by match_id;
run;
/* NOTE: The data set MYDATA.COHORT_FINAL_1_A has 142772 observations and 90
variables. */


proc print data=mydata.cohort_final_1_A ;
where statin=1 and "01JUL2007"D <= index_dt_new <= "12DEC2007"D;
var patient_id index_dt_new dx_date statin testosterone exposed match_id;
run;
/* NOTE: There were 7351 observations read from the data set
MYDATA.COHORT_FINAL_1_A. */


proc print data=mydata.cohort_final_1_A;
where testosterone=1 and "01JUL2007"D <= index_dt_new <= "12DEC2007"D;
var patient_id index_dt_new dx_date statin testosterone exposed match_id;
run;
/* NOTE: There were 47 observations read from the data set
MYDATA.COHORT_FINAL_1_A. */

proc print data=mydata.cohort_final_1_A;
```

```
where statin=1 and testosterone=1 and "01JUL2007"D <= index_dt_new <=
"12DEC2007"D;
var patient_id index_dt_new dx_date statin testosterone exposed match_id;
run;
/* NOTE: There were 31 observations read from the data set
MYDATA.COHORT_FINAL_1_A. */


data exclude_sta;
set mydata.cohort_final_1_A;
if "01JUL2007"D <= index_dt_new <= "12DEC2007"D and statin=1;
proc sort; by patient_id;
run;
/* NOTE: The data set WORK.EXCLUDE_STA has 7351 observations and 90
variables. */

data exclude_tes;
set mydata.cohort_final_1_A;
if "01JUL2007"D <= index_dt_new <= "12DEC2007"D and testosterone=1;
proc sort; by patient_id;
run;
/* NOTE: The data set WORK.EXCLUDE_TES has 47 observations and 90 variables.
*/


data exclude;
merge exclude_sta exclude_tes;
by patient_id;
run;
/*
NOTE: There were 7351 observations read from the data set WORK.EXCLUDE_STA.
NOTE: There were 47 observations read from the data set WORK.EXCLUDE_TES.
NOTE: The data set WORK.EXCLUDE has 7367 observations and 90 variables.
 */

proc sql;
create table mydata.cohort_final_1_A_B
as select * from mydata.cohort_final_1_A
where match_id not in (select match_id from exclude);
quit;
/* NOTE: Table MYDATA.COHORT_FINAL_1_A_B created, with 128038 rows and 90
columns. */
```
