# Supplementary material for: Obesity-Related Cancers in Relation to Use of Statins and Testosterone Replacement Therapy Among Older Women: SEER-Medicare 2007–2015
Source: Pharmaceuticals (Basel). 2025 Sep 19;18(9):1413. doi: 10.3390/ph18091413 (PMC12472775; doi:10.3390/ph18091413)
Supplement: Supplementary file 1 [file pharmaceuticals-18-01413-s001.zip › Supplementary material S3. SAS code for statistical analysis.pdf]

Supplementary Material S3. SAS code for statistical analysis

```
/*****************************************************************************
************************************************/
/* DATE  : 07/17/2021
*/
/* AUTHOR: Biai Digbeu
*/
/* DATA  : Medicare SEER data
*/
/* Title: The effect of testosterone replacement therapy and statins on
cardiovascular diseases among hormone-related cancers
   in women: SEER-Medicare 2007-2016 Cohort selection (CASE)
*/
/*****************************************************************************
************************************************/


/*Create libraries*/
libname SEER_c 'X:\';
libname SEER_nc 'W:\';
libname data 'Z:\Projects\Digbeu-B\Testosterone plus statins w CVDs among
cancer survivors women\Data';
libname mydata 'Z:\Projects\Digbeu-B\Testosterone plus statins w CVDs among
cancer survivors women\Mydata';
run;

proc format;
 value $ dc '0'='None'
            '1'='Statin alone'
                  '2'='Testosterone alone'
                  '3'='Both drugs';
run;


/* Summary statistics */
proc contents varnum data=mydata.cohort_final_1;
run;

/* Demographics summary using a macro */

*Convert numeric to categorical variables;
%macro char(var);
data mydata.cohort_final_1_A;
set mydata.cohort_final_1_A;
&var._new=put(&var, 1.);
drop &var;
rename &var._new=&var;
run;
%mend;

%char(bca)
%char(cca)
%char(oca)
%char(eca)
%char(advanced_cancer_stage)
```

```
%char(high_tumor_grade)
%char(diabetes)
%char(hypogonadism)
%char(hypertension)
%char(wasting)
%char(malaise)
%char(osteoporosis)
%char(pituitary_dysf)
%char(depression)
%char(hyperlipidemia)
%char(cardiovascular)
%char(insulin)
%char(cci_cat)
%char(drug_cat)
%char(statin)
%char(testosterone)
%char(death)
%char(cancer_diagnosed)
%char(exposed)

proc format;
 value $ dc '0'='None'
            '1'='Statin alone'
                  '2'='Testosterone alone'
                  '3'='Both drugs';
run;

data mydata.cohort_final_1_A;
set mydata.cohort_final_1_A;
format drug_cat $dc.;
run;
/* NOTE: The data set MYDATA.COHORT_FINAL_1_A has 142772 observations and 90
variables. */



proc contents varnum data=mydata.cohort_final_1_A;
run;

/* Summary for all patients */
libname mydata 'Z:\Projects\Digbeu-B\Testosterone plus statins w CVDs among
cancer survivors women\Mydata';
run;

 %macro categ(data, class, pred);

 proc freq data = &data;
  tables &pred * &class/chisq sparse outpct out=outfreq;
  output out = stats chisq;
 run;

 proc sort data = outfreq;
  by &pred;
 run;

 proc means data = outfreq noprint;
```

```
  where &class ne '' ;
  by &pred;
  var count;
  output out = moutfreq (keep= &pred total rename=(&pred=variable)) sum
=total;
 run;

 data routfreq1 (rename=(&pred=variable));
  set outfreq;
  length varname $20.;
  if &class = 0 and (&pred ne . or &pred ne .)      /*this will create two
columns for variable=1 and =0. If more categories need to add here*/
    then do;
     rcount = put(count,8.);
     rcount = trim(left(rcount));
     pctnum = (rcount)||" "||"("||trim(left(round(pct_col, 0.01)))||")";
    end;
  else if &class = 1 and (&pred ne . or &pred ne .)
    then do;
     rcount1 = put(count,8.);
     rcount1 = trim(left(rcount1));
     pctnum1 = (rcount1)||" "||"("||trim(left(round(pct_col, 0.01)))||")";
     end;
 else if &class = 2 and (&pred ne . or &pred ne .)
    then do;
     rcount2 = put(count,8.);
     rcount2 = trim(left(rcount2));
     pctnum2 = (rcount2)||" "||"("||trim(left(round(pct_col, 0.01)))||")";
     end;
 else if &class = 3 and (&pred ne . or &pred ne .)
    then do;
     rcount3 = put(count,8.);
     rcount3 = trim(left(rcount3));
     pctnum3 = (rcount3)||" "||"("||trim(left(round(pct_col, 0.01)))||")";
     end;
  else delete;
   index=1;
   varname=vlabel(&pred);
  keep &pred pctnum pctnum1 pctnum2 pctnum3 index varname;
 run;

 data routfreq;
 update routfreq1 (obs=0) routfreq1;
  by variable varname;
 run;

 data rstats;
 set stats;
  length p_value $8.;
  if P_PCHI <=0.0001 then p_value = 0.0001;
  else if P_PCHI <= 0.05 then p_value = put(P_PCHI,8.4);
  else p_value = put(P_PCHI,8.4);
 keep p_value index;
 index=1;
 run;

 data _null_;
```

```
 set &data;
 call symput("fmt",vformat(&pred) );
 run;

 proc sort data = moutfreq;by variable; run;
 proc sort data = routfreq; by variable;run;

 data temp;
 merge moutfreq routfreq;
 by variable;
 run;

 data tab;
 merge temp rstats;
 by index;
 length formats $30.;
 formats=put(variable,&fmt);
 if not first.index then do;
 varname = " ";
 p_value = " ";
 end;
 drop variable;
 run;
%mend;

%let varList = %str(bca cca oca eca advanced_cancer_stage high_tumor_grade
age_cat white black hisp other hyperlipidemia
                    hypogonadism hypertension  wasting
                    malaise osteoporosis pituitary_dysf depression  diabetes
cardiovascular insulin cci_cat);


%macro repeat(data, class, predList);
    %let i = 1;
    %let predictor = %scan(&predList, &i);

    %do %while ("&predictor" ^= "");
        %categ(&data, &class, &predictor);

        proc datasets lib = work memtype = data nodetails nolist nowarn;
        append base = final data = tab; quit;

        %let i = %eval(&i + 1);
        %let predictor = %scan(&predList, &i);
    %end;
%mend;

%repeat(mydata.cohort_final_1_A, drug_cat, &varList);

data categ_report;
 set final;
 label varname = "Characteristics"
 total = "Total"
 pctnum = "No drug* N (%)"
 pctnum1 = "Statin * N (%)"
 pctnum2 = "TTH* N (%)"
 pctnum3 = "Both * N (%)"
```

```
 p_value = "p-value"
 formats = "Category";
 run;

ods listing close;
ods rtf file = "Z:\Projects\Digbeu-B\Testosterone plus statins w CVDs among
cancer survivors women\SAS output\summary_1.rtf";
proc report data = categ_report nowd split = "*";
 column index varname formats total pctnum pctnum1 pctnum2 pctnum3 p_value;
 define index /group noprint;
 define varname / order = data style(column) = [just=left] width = 40;
 define formats / order = data style(column) = [just=left];
 define total / order = data style(column) = [just=center];
 define pctnum / order = data style(column) = [just=center];
 define pctnum1 / order = data style(column) = [just=center];
 define pctnum2 / order = data style(column) = [just=center];
 define pctnum3 / order = data style(column) = [just=center];
 define p_value / order = data style(column) = [just=center];
run;
ods rtf close;
ods listing;

proc means data=mydata.cohort_final_1_A;
class drug_cat;
var  breast_test_no col_test_no ov_test_no psa_test_no visits_no education
poverty;
run;

data mydata.cohort_final_1_A;
set mydata.cohort_final_1_A;
drug_cat_new=drug_cat*1; /* Numeric version of the drug_cat variable */
run;


proc glm data=mydata.cohort_final_1_A;
model drug_cat_new= breast_test_no;
run;

proc glm data=mydata.cohort_final_1_A;
model drug_cat_new= col_test_no;
run;

proc glm data=mydata.cohort_final_1_A;
model drug_cat_new= ov_test_no;
run;


proc glm data=mydata.cohort_final_1_A;
model drug_cat_new= psa_test_no ;
run;

proc glm data=mydata.cohort_final_1_A;
model drug_cat_new= visits_no;
run;

proc glm data=mydata.cohort_final_1_A;
model drug_cat_new= education;
```

```
run;

proc glm data=mydata.cohort_final_1_A;
model drug_cat_new=poverty;
run;

proc freq data=mydata.cohort_final_1_A;
table race_cat*drug_cat/chisq fisher;
run;

proc freq data=mydata.cohort_final_1_A;
table death*drug_cat/chisq fisher;
run;


/* Summary among HRC patients */
libname mydata 'Z:\Projects\Digbeu-B\Testosterone plus statins w CVDs among
cancer survivors women\Mydata';
run;

 %macro categ(data, class, pred);

 proc freq data = &data;
 where cancer_diagnosed='1';
  tables &pred * &class/chisq sparse outpct out=outfreq;
  output out = stats chisq;
 run;

 proc sort data = outfreq;
  by &pred;
 run;

 proc means data = outfreq noprint;
  where &class ne '' ;
  by &pred;
  var count;
  output out = moutfreq (keep= &pred total rename=(&pred=variable)) sum
=total;
 run;

 data routfreq1 (rename=(&pred=variable));
  set outfreq;
  length varname $20.;
  if &class = 0 and (&pred ne . or &pred ne .)       /*this will create two
columns for variable=1 and =0. If more categories need to add here*/
    then do;
     rcount = put(count,8.);
     rcount = trim(left(rcount));
     pctnum = (rcount)||" "||"("||trim(left(round(pct_col, 0.01)))||")";
    end;
  else if &class = 1 and (&pred ne . or &pred ne .)
    then do;
     rcount1 = put(count,8.);
     rcount1 = trim(left(rcount1));
     pctnum1 = (rcount1)||" "||"("||trim(left(round(pct_col, 0.01)))||")";
     end;
 else if &class = 2 and (&pred ne . or &pred ne .)
```

```
    then do;
     rcount2 = put(count,8.);
     rcount2 = trim(left(rcount2));
     pctnum2 = (rcount2)||" "||"("||trim(left(round(pct_col, 0.01)))||")";
     end;
 else if &class = 3 and (&pred ne . or &pred ne .)
    then do;
     rcount3 = put(count,8.);
     rcount3 = trim(left(rcount3));
     pctnum3 = (rcount3)||" "||"("||trim(left(round(pct_col, 0.01)))||")";
     end;
  else delete;
   index=1;
   varname=vlabel(&pred);
  keep &pred pctnum pctnum1 pctnum2 pctnum3 index varname;
 run;

 data routfreq;
 update routfreq1 (obs=0) routfreq1;
  by variable varname;
 run;

 data rstats;
 set stats;
  length p_value $8.;
  if P_PCHI <=0.0001 then p_value = 0.0001;
  else if P_PCHI <= 0.05 then p_value = put(P_PCHI,8.4);
  else p_value = put(P_PCHI,8.4);
 keep p_value index;
 index=1;
 run;

 data _null_;
 set &data;
 call symput("fmt",vformat(&pred) );
 run;

 proc sort data = moutfreq;by variable; run;
 proc sort data = routfreq; by variable;run;

 data temp;
 merge moutfreq routfreq;
 by variable;
 run;

 data tab;
 merge temp rstats;
 by index;
 length formats $30.;
 formats=put(variable,&fmt);
 if not first.index then do;
 varname = " ";
 p_value = " ";
 end;
 drop variable;
 run;
%mend;
```

```
%let varList = %str(bca cca oca eca advanced_cancer_stage high_tumor_grade
age_cat white black hisp other hyperlipidemia
                    hypogonadism hypertension  wasting
                    malaise osteoporosis pituitary_dysf depression  diabetes
cardiovascular insulin cci_cat);


%macro repeat(data, class, predList);
    %let i = 1;
    %let predictor = %scan(&predList, &i);

    %do %while ("&predictor" ^= "");
        %categ(&data, &class, &predictor);

        proc datasets lib = work memtype = data nodetails nolist nowarn;
        append base = final data = tab; quit;

        %let i = %eval(&i + 1);
        %let predictor = %scan(&predList, &i);
    %end;
%mend;

%repeat(mydata.cohort_final_1_A, drug_cat, &varList);

data categ_report;
 set final;
 label varname = "Characteristics"
 total = "Total"
 pctnum = "No drug* N (%)"
 pctnum1 = "Statin * N (%)"
 pctnum2 = "TTH* N (%)"
 pctnum3 = "Both * N (%)"
 p_value = "p-value"
 formats = "Category";
 run;

ods listing close;
ods rtf file = "Z:\Projects\Digbeu-B\Testosterone plus statins w CVDs among
cancer survivors women\SAS output\summary1_1.rtf";
proc report data = categ_report nowd split = "*";
 column index varname formats total pctnum pctnum1 pctnum2 pctnum3 p_value;
 define index /group noprint;
 define varname / order = data style(column) = [just=left] width = 40;
 define formats / order = data style(column) = [just=left];
 define total / order = data style(column) = [just=center];
 define pctnum / order = data style(column) = [just=center];
 define pctnum1 / order = data style(column) = [just=center];
 define pctnum2 / order = data style(column) = [just=center];
 define pctnum3 / order = data style(column) = [just=center];
 define p_value / order = data style(column) = [just=center];
run;
ods rtf close;
ods listing;


proc means data=mydata.cohort_final_1_A;
```

```
class drug_cat;
where cancer_diagnosed='1';
var  breast_test_no col_test_no ov_test_no psa_test_no visits_no education
poverty;
run;


proc glm data=mydata.cohort_final_1_A;
where cancer_diagnosed='1';
model drug_cat_new= breast_test_no;
run;

proc glm data=mydata.cohort_final_1_A;
where cancer_diagnosed='1';
model drug_cat_new= col_test_no;
run;

proc glm data=mydata.cohort_final_1_A;
where cancer_diagnosed='1';
model drug_cat_new= ov_test_no;
run;


proc glm data=mydata.cohort_final_1_A;
where cancer_diagnosed='1';
model drug_cat_new= psa_test_no ;
run;

proc glm data=mydata.cohort_final_1_A;
where cancer_diagnosed='1';
model drug_cat_new= visits_no;
run;

proc glm data=mydata.cohort_final_1_A;
where cancer_diagnosed='1';
model drug_cat_new= education;
run;

proc glm data=mydata.cohort_final_1_A;
where cancer_diagnosed='1';
model drug_cat_new=poverty;
run;

proc freq data=mydata.cohort_final_1_A;
where cancer_diagnosed='1';
table race_cat*drug_cat/ chisq fisher;
run;

proc freq data=mydata.cohort_final_1_A;
where cancer_diagnosed='1';
table death*drug_cat/ chisq fisher;
run;



/* Summary among cancer-free patients */
```

```
libname mydata 'Z:\Projects\Digbeu-B\Testosterone plus statins w CVDs among
cancer survivors women\Mydata';
run;


 %macro categ(data, class, pred);

 proc freq data = &data;
 where cancer_diagnosed='0';
  tables &pred * &class/chisq sparse outpct out=outfreq;
  output out = stats chisq;
 run;

 proc sort data = outfreq;
  by &pred;
 run;

 proc means data = outfreq noprint;
  where &class ne '' ;
  by &pred;
  var count;
  output out = moutfreq (keep= &pred total rename=(&pred=variable)) sum
=total;
 run;

 data routfreq1 (rename=(&pred=variable));
  set outfreq;
  length varname $20.;
  if &class = 0 and (&pred ne . or &pred ne .)       /*this will create two
columns for variable=1 and =0. If more categories need to add here*/
    then do;
     rcount = put(count,8.);
     rcount = trim(left(rcount));
     pctnum = (rcount)||" "||"("||trim(left(round(pct_col, 0.01)))||")";
    end;
  else if &class = 1 and (&pred ne . or &pred ne .)
    then do;
     rcount1 = put(count,8.);
     rcount1 = trim(left(rcount1));
     pctnum1 = (rcount1)||" "||"("||trim(left(round(pct_col, 0.01)))||")";
     end;
 else if &class = 2 and (&pred ne . or &pred ne .)
    then do;
     rcount2 = put(count,8.);
     rcount2 = trim(left(rcount2));
     pctnum2 = (rcount2)||" "||"("||trim(left(round(pct_col, 0.01)))||")";
     end;
 else if &class = 3 and (&pred ne . or &pred ne .)
    then do;
     rcount3 = put(count,8.);
     rcount3 = trim(left(rcount3));
     pctnum3 = (rcount3)||" "||"("||trim(left(round(pct_col, 0.01)))||")";
     end;
  else delete;
   index=1;
   varname=vlabel(&pred);
  keep &pred pctnum pctnum1 pctnum2 pctnum3 index varname;
```

```
 run;

 data routfreq;
 update routfreq1 (obs=0) routfreq1;
  by variable varname;
 run;

 data rstats;
 set stats;
  length p_value $8.;
  if P_PCHI <=0.0001 then p_value = 0.0001;
  else if P_PCHI <= 0.05 then p_value = put(P_PCHI,8.4);
  else p_value = put(P_PCHI,8.4);
 keep p_value index;
 index=1;
 run;

 data _null_;
 set &data;
 call symput("fmt",vformat(&pred) );
 run;

 proc sort data = moutfreq;by variable; run;
 proc sort data = routfreq; by variable;run;

 data temp;
 merge moutfreq routfreq;
 by variable;
 run;

 data tab;
 merge temp rstats;
 by index;
 length formats $30.;
 formats=put(variable,&fmt);
 if not first.index then do;
 varname = " ";
 p_value = " ";
 end;
 drop variable;
 run;
%mend;

%let varList = %str(bca cca oca eca advanced_cancer_stage high_tumor_grade
age_cat white black hisp other hyperlipidemia
                    hypogonadism hypertension  wasting
                    malaise osteoporosis pituitary_dysf depression  diabetes
cardiovascular insulin cci_cat);


%macro repeat(data, class, predList);
    %let i = 1;
    %let predictor = %scan(&predList, &i);

    %do %while ("&predictor" ^= "");
        %categ(&data, &class, &predictor);
```

```
        proc datasets lib = work memtype = data nodetails nolist nowarn;
        append base = final data = tab; quit;

        %let i = %eval(&i + 1);
        %let predictor = %scan(&predList, &i);
    %end;
%mend;

%repeat(mydata.cohort_final_1_A, drug_cat, &varList);

data categ_report;
 set final;
 label varname = "Characteristics"
 total = "Total"
 pctnum = "No drug* N (%)"
 pctnum1 = "Statin * N (%)"
 pctnum2 = "TTH* N (%)"
 pctnum3 = "Both * N (%)"
 p_value = "p-value"
 formats = "Category";
 run;

ods listing close;
ods rtf file = "Z:\Projects\Digbeu-B\Testosterone plus statins w CVDs among
cancer survivors women\SAS output\summary2_1.rtf";
proc report data = categ_report nowd split = "*";
 column index varname formats total pctnum pctnum1 pctnum2 pctnum3 p_value;
 define index /group noprint;
 define varname / order = data style(column) = [just=left] width = 40;
 define formats / order = data style(column) = [just=left];
 define total / order = data style(column) = [just=center];
 define pctnum / order = data style(column) = [just=center];
 define pctnum1 / order = data style(column) = [just=center];
 define pctnum2 / order = data style(column) = [just=center];
 define pctnum3 / order = data style(column) = [just=center];
 define p_value / order = data style(column) = [just=center];
run;
ods rtf close;
ods listing;



proc means data=mydata.cohort_final_1_A;
class drug_cat;
where cancer_diagnosed='0';
var  breast_test_no col_test_no ov_test_no psa_test_no visits_no education
poverty;
run;


proc glm data=mydata.cohort_final_1_A;
where cancer_diagnosed='0';
model drug_cat_new= breast_test_no;
run;

proc glm data=mydata.cohort_final_1_A;
where cancer_diagnosed='0';
```

```
model drug_cat_new= col_test_no;
run;

proc glm data=mydata.cohort_final_1_A;
where cancer_diagnosed='0';
model drug_cat_new= ov_test_no;
run;


proc glm data=mydata.cohort_final_1_A;
where cancer_diagnosed='0';
model drug_cat_new= psa_test_no ;
run;

proc glm data=mydata.cohort_final_1_A;
where cancer_diagnosed='0';
model drug_cat_new= visits_no;
run;

proc glm data=mydata.cohort_final_1_A;
where cancer_diagnosed='0';
model drug_cat_new= education;
run;

proc glm data=mydata.cohort_final_1_A;
where cancer_diagnosed='0';
model drug_cat_new=poverty;
run;

proc freq data=mydata.cohort_final_1_A;
where cancer_diagnosed='0';
table race_cat*drug_cat/ chisq fisher;
run;

proc freq data=mydata.cohort_final_1_A;
where cancer_diagnosed='0';
table death*drug_cat/ chisq fisher;
run;

/* Data analysis */

proc contents varnum data=mydata.cohort_final_1_A;
run;

/* Create weight */

data mydata.cohort_final_1_A;
set mydata.cohort_final_1_A;
if cancer_diagnosed='1' then weight=1; else weight=20;
run;
/* NOTE: The data set MYDATA.COHORT_FINAL_1_A has 142772 observations and 92
variables. */


proc freq data=mydata.cohort_final_1_A;
where exposed='1';
table statin testosterone both_drugs;
```

```
run;


proc freq data=mydata.cohort_final_1_A;
table statin testosterone both_drugs;
run;

proc freq data=mydata.cohort_final_1_A;
table testosterone*death/chisq fisher;
where bca='1';
run;

proc freq data=mydata.cohort_final_1_A;
table testosterone*death/chisq fisher;
where cca='1';
run;

proc freq data=mydata.cohort_final_1_A;
table testosterone*death/chisq fisher;
where oca='1';
run;

proc freq data=mydata.cohort_final_1_A;
table testosterone*death/chisq fisher;
where eca='1';
run;

/* Reframe education and poverty variables */
data mydata.cohort_final_1_A;
set mydata.cohort_final_1_A;
education_new=education;
if education_new=. then education_new=0;
poverty_new=poverty;
if poverty_new=. then poverty_new=0;
run;
/* NOTE: The data set MYDATA.COHORT_FINAL_1_A has 142772 observations and 94
variables. */


/* Analysis */

proc contents varnum data=mydata.cohort_final_1_A; run;

proc freq data=mydata.cohort_final_1_A; table cancer; run;

/* Cox regression analysis */

proc means data=mydata.cohort_final_1_A;;
var age_exposed visits_no education education_new poverty poverty_new
breast_test_no col_test_no ov_test_no;
run;

proc freq data=mydata.cohort_final_1_A;;
table education poverty;
run;
```

```
/* 1-outcome= HRCs versus non-cancer cases (control) */

ods rtf file='Z:\Projects\Digbeu-B\Testosterone plus statins w CVDs among
cancer survivors women\SAS output\Analysis_output_Statin_TTH.rtf';

title 'All cancers (breast, colorectal, ovarian, endometrial) -
outcome=Incident HRC';

proc freq data=mydata.cohort_final_1_A; table cancer_diagnosed; run;

proc freq data=mydata.cohort_final_1_A; table (statin testosterone
drug_cat)*cancer_diagnosed; weight weight; run;

/* Create time variable */
data mydata.cohort_final_1_A;

set mydata.cohort_final_1_A;

if cancer_diagnosed='1' then timeA=dx_date-index_dt_new; else
timeA="01DEC2016"D-index_dt_new;

cancer_diagnosed_new=cancer_diagnosed*1; *Numeric version of the variable;

run;

proc freq data=mydata.cohort_final_1_A; table timeA; run; /* Min tim = 6
months */


proc surveyphreg data=mydata.cohort_final_1_A;

class white(ref='0') black(ref='0') hisp(ref='0')

      diabetes(ref='0') hypertension(ref='0') wasting(ref='0')
malaise(ref='0') osteoporosis(ref='0') pituitary_dysf(ref='0')

      depression(ref='0') hyperlipidemia(ref='0') cardiovascular(ref='0')
insulin(ref='0') cci_cat(ref='0')  statin(ref='0')/param=ref;

strata match_id;

model timeA*cancer_diagnosed_new(0)=age_exposed white black hisp diabetes
hypertension wasting malaise osteoporosis pituitary_dysf

            depression hyperlipidemia cardiovascular insulin cci_cat
visits_no education poverty breast_test_no col_test_no ov_test_no

            statin/risklimits;

weight weight;

run;


proc surveyphreg data=mydata.cohort_final_1_A;

class white(ref='0') black(ref='0') hisp(ref='0')
```

```
      diabetes(ref='0') hypertension(ref='0') wasting(ref='0')
malaise(ref='0') osteoporosis(ref='0') pituitary_dysf(ref='0')

      depression(ref='0') hyperlipidemia(ref='0') cardiovascular(ref='0')
insulin(ref='0') cci_cat(ref='0')  testosterone(ref='0')/param=ref;

strata match_id;

model timeA*cancer_diagnosed_new(0)=age_exposed white black hisp diabetes
hypertension wasting malaise osteoporosis pituitary_dysf

            depression hyperlipidemia cardiovascular insulin cci_cat
visits_no education poverty breast_test_no col_test_no ov_test_no

            testosterone/risklimits;

weight weight;

run;


proc surveyphreg data=mydata.cohort_final_1_A;

class white(ref='0') black(ref='0') hisp(ref='0')

      diabetes(ref='0') hypertension(ref='0') wasting(ref='0')
malaise(ref='0') osteoporosis(ref='0') pituitary_dysf(ref='0')

      depression(ref='0') hyperlipidemia(ref='0') cardiovascular(ref='0')
insulin(ref='0') cci_cat(ref='0') drug_cat(ref='None')/param=ref;

strata match_id;

model timeA*cancer_diagnosed_new(0)=age_exposed white black hisp diabetes
hypertension wasting malaise osteoporosis pituitary_dysf

            depression hyperlipidemia cardiovascular insulin cci_cat
visits_no education poverty breast_test_no col_test_no ov_test_no

            drug_cat/risklimits;

weight weight;

run;

*Survival curve to verify if statin users are taking much more longer time to
get diagnosed with cancers compared to
non-statin users;

ods graphics on;

title 'Kaplan-Meier Survival Curve for time until death by post-diagnosis
radiation with number at risk';

proc lifetest data=mydata.cohort_final_1_A/* plots=s(test
atrisk(atrisktickonly maxlen=13 outside)=0 365 730 1095 1460 1825)*/;
```

```
time timeA*cancer_diagnosed_new(0);

strata STA/order=internal;

ods output survivalplot=sp;

run;

ods graphics off;

**Among breast cancer only;

title 'Among breast cancer only - outcome=incident HRC';

proc freq data=mydata.cohort_final_1_A; table cancer*cancer_diagnosed; run;

data mydata.cohort_final_1_A;

set mydata.cohort_final_1_A;

if cancer='1' and cancer_diagnosed='1' then breast_cancer_diag=1;  **Breast
cancer only;

else if cancer_diagnosed='0' then breast_cancer_diag=0; **No cancer at all;

else breast_cancer_diag=.;

run;

proc freq data=mydata.cohort_final_1_A; table breast_cancer_diag
statin*breast_cancer_diag; run;

proc freq data=mydata.cohort_final_1_A; table (statin testosterone
drug_cat)*breast_cancer_diag; weight weight; run;

proc surveyphreg data=mydata.cohort_final_1_A;

class white(ref='0') black(ref='0') hisp(ref='0')

      diabetes(ref='0') hypertension(ref='0') wasting(ref='0')
malaise(ref='0') osteoporosis(ref='0') pituitary_dysf(ref='0')

      depression(ref='0') hyperlipidemia(ref='0') cardiovascular(ref='0')
insulin(ref='0') cci_cat(ref='0')  statin(ref='0')/param=ref;

strata match_id;

model timeA*breast_cancer_diag(0)=age_exposed white black hisp diabetes
hypertension wasting malaise osteoporosis pituitary_dysf

            depression hyperlipidemia cardiovascular insulin cci_cat
visits_no education poverty breast_test_no /*col_test_no ov_test_no*/

            statin/risklimits;

weight weight;
```

```
run;

proc surveyphreg data=mydata.cohort_final_1_A;

class white(ref='0') black(ref='0') hisp(ref='0')

      diabetes(ref='0') hypertension(ref='0') wasting(ref='0')
malaise(ref='0') osteoporosis(ref='0') pituitary_dysf(ref='0')

      depression(ref='0') hyperlipidemia(ref='0') cardiovascular(ref='0')
insulin(ref='0') cci_cat(ref='0')  testosterone(ref='0')/param=ref;

strata match_id;

model timeA*breast_cancer_diag(0)=age_exposed white black hisp diabetes
hypertension wasting malaise osteoporosis pituitary_dysf

            depression hyperlipidemia cardiovascular insulin cci_cat
visits_no education poverty breast_test_no /*col_test_no ov_test_no*/

            testosterone/risklimits;

weight weight;

run;

proc surveyphreg data=mydata.cohort_final_1_A;

class white(ref='0') black(ref='0') hisp(ref='0')

      diabetes(ref='0') hypertension(ref='0') wasting(ref='0')
malaise(ref='0') osteoporosis(ref='0') pituitary_dysf(ref='0')

      depression(ref='0') hyperlipidemia(ref='0') cardiovascular(ref='0')
insulin(ref='0') cci_cat(ref='0')  drug_cat(ref='None')/param=ref;

strata match_id;

model timeA*breast_cancer_diag(0)=age_exposed white black hisp diabetes
hypertension wasting malaise osteoporosis pituitary_dysf

            depression hyperlipidemia cardiovascular insulin cci_cat
visits_no education poverty breast_test_no /*col_test_no ov_test_no*/

            drug_cat/risklimits;

weight weight;

run;


**Among colorectal cancer only;

title 'Among colorectal cancer only - outcome=incident HRC';

proc freq data=mydata.cohort_final_1_A; table cancer*cancer_diagnosed; run;
```

```
data mydata.cohort_final_1_A;

set mydata.cohort_final_1_A;

if cancer='2' and cancer_diagnosed='1' then colorectal_cancer_diag=1;
**Colorectal cancer only;

else if cancer_diagnosed='0' then colorectal_cancer_diag=0; **No cancer at
all;

else colorectal_cancer_diag=.;

run;

proc freq data=mydata.cohort_final_1_A; table colorectal_cancer_diag; run;


proc freq data=mydata.cohort_final_1_A; table (statin testosterone
drug_cat)*colorectal_cancer_diag; weight weight; run;

proc surveyphreg data=mydata.cohort_final_1_A;

class white(ref='0') black(ref='0') hisp(ref='0')

      diabetes(ref='0') hypertension(ref='0') wasting(ref='0')
malaise(ref='0') osteoporosis(ref='0') pituitary_dysf(ref='0')

      depression(ref='0') hyperlipidemia(ref='0') cardiovascular(ref='0')
insulin(ref='0') cci_cat(ref='0')  statin(ref='0')/param=ref;

strata match_id;

model timeA*colorectal_cancer_diag(0)=age_exposed white black hisp diabetes
hypertension wasting malaise osteoporosis pituitary_dysf

            depression hyperlipidemia cardiovascular insulin cci_cat
visits_no education poverty /*breast_test_no*/ col_test_no /*ov_test_no*/

            statin/risklimits;

weight weight;

run;


proc surveyphreg data=mydata.cohort_final_1_A;

class white(ref='0') black(ref='0') hisp(ref='0')

      diabetes(ref='0') hypertension(ref='0') wasting(ref='0')
malaise(ref='0') osteoporosis(ref='0') pituitary_dysf(ref='0')

      depression(ref='0') hyperlipidemia(ref='0') cardiovascular(ref='0')
insulin(ref='0') cci_cat(ref='0')  testosterone(ref='0')/param=ref;

strata match_id;
```

```
model timeA*colorectal_cancer_diag(0)=age_exposed white black hisp diabetes
hypertension wasting malaise osteoporosis pituitary_dysf

            depression hyperlipidemia cardiovascular insulin cci_cat
visits_no education poverty /*breast_test_no*/ col_test_no /*ov_test_no*/

            testosterone/risklimits;

weight weight;

run;


proc surveyphreg data=mydata.cohort_final_1_A;

class white(ref='0') black(ref='0') hisp(ref='0')

      diabetes(ref='0') hypertension(ref='0') wasting(ref='0')
malaise(ref='0') osteoporosis(ref='0') pituitary_dysf(ref='0')

      depression(ref='0') hyperlipidemia(ref='0') cardiovascular(ref='0')
insulin(ref='0') cci_cat(ref='0')  drug_cat(ref='None')/param=ref;

strata match_id;

model timeA*colorectal_cancer_diag(0)=age_exposed white black hisp diabetes
hypertension wasting malaise osteoporosis pituitary_dysf

            depression hyperlipidemia cardiovascular insulin cci_cat
visits_no education poverty /*breast_test_no*/ col_test_no /*ov_test_no*/

            drug_cat/risklimits;

weight weight;

run;



**Among ovarian cancer only;

title 'Among ovarian cancer only  - outcome=incident HRC';

proc freq data=mydata.cohort_final_1_A; table cancer*cancer_diagnosed; run;

data mydata.cohort_final_1_A;

set mydata.cohort_final_1_A;

if cancer='3' and cancer_diagnosed='1' then ovarian_cancer_diag=1;  **Ovarian
cancer only;

else if cancer_diagnosed='0' then ovarian_cancer_diag=0; **No cancer at all;

else ovarian_cancer_diag=.;
```

```
run;

proc freq data=mydata.cohort_final_1_A; table ovarian_cancer_diag; run;


proc freq data=mydata.cohort_final_1_A; table (statin testosterone
drug_cat)*ovarian_cancer_diag; weight weight; run;

proc surveyphreg data=mydata.cohort_final_1_A;

class white(ref='0') black(ref='0') hisp(ref='0')

      diabetes(ref='0') hypertension(ref='0') wasting(ref='0')
malaise(ref='0') osteoporosis(ref='0') pituitary_dysf(ref='0')

      depression(ref='0') hyperlipidemia(ref='0') cardiovascular(ref='0')
insulin(ref='0') cci_cat(ref='0')  statin(ref='0')/param=ref;

strata match_id;

model timeA*ovarian_cancer_diag(0)=age_exposed white black hisp diabetes
hypertension wasting malaise osteoporosis pituitary_dysf

            depression hyperlipidemia cardiovascular insulin cci_cat
visits_no education poverty /*breast_test_no col_test_no*/ ov_test_no

            statin/risklimits;

weight weight;

run;


proc surveyphreg data=mydata.cohort_final_1_A;

class white(ref='0') black(ref='0') hisp(ref='0')

      diabetes(ref='0') hypertension(ref='0') wasting(ref='0')
malaise(ref='0') osteoporosis(ref='0') pituitary_dysf(ref='0')

      depression(ref='0') hyperlipidemia(ref='0') cardiovascular(ref='0')
insulin(ref='0') cci_cat(ref='0')  testosterone(ref='0')/param=ref;

strata match_id;

model timeA*ovarian_cancer_diag(0)=age_exposed white black hisp diabetes
hypertension wasting malaise osteoporosis pituitary_dysf

            depression hyperlipidemia cardiovascular insulin cci_cat
visits_no education poverty /*breast_test_no col_test_no*/ ov_test_no

            testosterone/risklimits;

weight weight;

run;
```

```
proc surveyphreg data=mydata.cohort_final_1_A;

class white(ref='0') black(ref='0') hisp(ref='0')

      diabetes(ref='0') hypertension(ref='0') wasting(ref='0')
malaise(ref='0') osteoporosis(ref='0') pituitary_dysf(ref='0')

      depression(ref='0') hyperlipidemia(ref='0') cardiovascular(ref='0')
insulin(ref='0') cci_cat(ref='0')  drug_cat(ref='None')/param=ref;

strata match_id;

model timeA*ovarian_cancer_diag(0)=age_exposed white black hisp diabetes
hypertension wasting malaise osteoporosis pituitary_dysf

            depression hyperlipidemia cardiovascular insulin cci_cat
visits_no education poverty /*breast_test_no col_test_no*/ ov_test_no

            drug_cat/risklimits;

weight weight;

run;



**Among endometrial cancer only;

title 'Among endometrial cancer only  - outcome=incident HRC';

proc freq data=mydata.cohort_final_1_A; table cancer*cancer_diagnosed; run;

data mydata.cohort_final_1_A;

set mydata.cohort_final_1_A;

if cancer='4' and cancer_diagnosed='1' then endo_cancer_diag=1;
**Endometrial cancer only;

else if cancer_diagnosed='0' then endo_cancer_diag=0; **No cancer at all;

else endo_cancer_diag=.;

run;

proc freq data=mydata.cohort_final_1_A; table endo_cancer_diag; run;


proc freq data=mydata.cohort_final_1_A; table (statin testosterone
drug_cat)*endo_cancer_diag; weight weight; run;

proc surveyphreg data=mydata.cohort_final_1_A;

class white(ref='0') black(ref='0') hisp(ref='0')
```

```
      diabetes(ref='0') hypertension(ref='0') wasting(ref='0')
malaise(ref='0') osteoporosis(ref='0') pituitary_dysf(ref='0')

      depression(ref='0') hyperlipidemia(ref='0') cardiovascular(ref='0')
insulin(ref='0') cci_cat(ref='0')  statin(ref='0')/param=ref;

strata match_id;

model timeA*endo_cancer_diag(0)=age_exposed white black hisp diabetes
hypertension wasting malaise osteoporosis pituitary_dysf

            depression hyperlipidemia cardiovascular insulin cci_cat
visits_no education poverty /*breast_test_no col_test_no ov_test_no*/

            statin/risklimits;

weight weight;

run;


proc surveyphreg data=mydata.cohort_final_1_A;

class white(ref='0') black(ref='0') hisp(ref='0')

      diabetes(ref='0') hypertension(ref='0') wasting(ref='0')
malaise(ref='0') osteoporosis(ref='0') pituitary_dysf(ref='0')

      depression(ref='0') hyperlipidemia(ref='0') cardiovascular(ref='0')
insulin(ref='0') cci_cat(ref='0')  testosterone(ref='0')/param=ref;

strata match_id;

model timeA*endo_cancer_diag(0)=age_exposed white black hisp diabetes
hypertension wasting malaise osteoporosis pituitary_dysf

            depression hyperlipidemia cardiovascular insulin cci_cat
visits_no education poverty /*breast_test_no col_test_no ov_test_no*/

            testosterone/risklimits;

weight weight;

run;

proc surveyphreg data=mydata.cohort_final_1_A;

class white(ref='0') black(ref='0') hisp(ref='0')

      diabetes(ref='0') hypertension(ref='0') wasting(ref='0')
malaise(ref='0') osteoporosis(ref='0') pituitary_dysf(ref='0')

      depression(ref='0') hyperlipidemia(ref='0') cardiovascular(ref='0')
insulin(ref='0') cci_cat(ref='0')  drug_cat(ref='None')/param=ref;

strata match_id;
```

```
model timeA*endo_cancer_diag(0)=age_exposed white black hisp diabetes
hypertension wasting malaise osteoporosis pituitary_dysf

            depression hyperlipidemia cardiovascular insulin cci_cat
visits_no education poverty /*breast_test_no col_test_no ov_test_no*/

            drug_cat/risklimits;

weight weight;

run;





/* 2-Outcome=high-grade HRCs versus non-cancer cases */

title 'All cancers (breast, colorectal, ovarian, endometrial) - outcome=High
grade cancer';

proc freq data=mydata.cohort_final_1_A; table
cancer_diagnosed*high_tumor_grade; run;


data mydata.cohort_final_1_A;
set mydata.cohort_final_1_A;
if cancer_diagnosed='1' and high_tumor_grade='1' then high='1'; *High tumor
grade among cancer patients only, exclude cancer  patients with non high
tumor grade;
if cancer_diagnosed='0' then high='0'; *Non-cancer patients, no existing high
tumor grade;
run;
/* NOTE: The data set MYDATA.COHORT_FINAL_1_A has 142772 observations and 101
variables. */


proc freq data=mydata.cohort_final_1_A; table high high*sta; run; /* OK */

proc freq data=mydata.cohort_final_1_A; table (statin testosterone
drug_cat)*high; weight weight; run;

/* Create time variable */
data mydata.cohort_final_1_A;
set mydata.cohort_final_1_A;
if high='1' then timeB=dx_date-index_dt_new; else timeB="31DEC2016"D-
index_dt_new;
high_new=high*1; *Numeric version of the variable;
run;

proc freq data=mydata.cohort_final_1_A; table timeB; run; /* OK */

proc surveyphreg data=mydata.cohort_final_1_A;

class white(ref='0') black(ref='0') hisp(ref='0')
```

```
      diabetes(ref='0') hypertension(ref='0') wasting(ref='0')
malaise(ref='0') osteoporosis(ref='0') pituitary_dysf(ref='0')

      depression(ref='0') hyperlipidemia(ref='0') cardiovascular(ref='0')
insulin(ref='0') cci_cat(ref='0')  statin(ref='0')/param=ref;

strata match_id;

model timeB*high_new(0)=age_exposed white black hisp diabetes hypertension
wasting malaise osteoporosis pituitary_dysf

            depression hyperlipidemia cardiovascular insulin cci_cat
visits_no education poverty breast_test_no col_test_no ov_test_no

            statin/risklimits;

weight weight;

run;


proc surveyphreg data=mydata.cohort_final_1_A;;

class white(ref='0') black(ref='0') hisp(ref='0')

      diabetes(ref='0') hypertension(ref='0') wasting(ref='0')
malaise(ref='0') osteoporosis(ref='0') pituitary_dysf(ref='0')

      depression(ref='0') hyperlipidemia(ref='0') cardiovascular(ref='0')
insulin(ref='0') cci_cat(ref='0')  testosterone(ref='0')/param=ref;

strata match_id;

model timeB*high_new(0)=age_exposed white black hisp diabetes hypertension
wasting malaise osteoporosis pituitary_dysf

            depression hyperlipidemia cardiovascular insulin cci_cat
visits_no education poverty breast_test_no col_test_no ov_test_no

            testosterone/risklimits;

weight weight;

run;



proc surveyphreg data=mydata.cohort_final_1_A;

class white(ref='0') black(ref='0') hisp(ref='0')

      diabetes(ref='0') hypertension(ref='0') wasting(ref='0')
malaise(ref='0') osteoporosis(ref='0') pituitary_dysf(ref='0')

      depression(ref='0') hyperlipidemia(ref='0') cardiovascular(ref='0')
insulin(ref='0') cci_cat(ref='0')  drug_cat(ref='None')/param=ref;
```

```
strata match_id;

model timeB*high_new(0)=age_exposed white black hisp diabetes hypertension
wasting malaise osteoporosis pituitary_dysf

            depression hyperlipidemia cardiovascular insulin cci_cat
visits_no education poverty breast_test_no col_test_no ov_test_no

            drug_cat/risklimits;

weight weight;

run;

**Among breast cancer only;

title 'Among breast cancer only - outcome=High grade cancer';

proc freq data=mydata.cohort_final_1_A; table
breast_cancer_diag*high_tumor_grade; run;

data mydata.cohort_final_1_A;
set mydata.cohort_final_1_A;
if breast_cancer_diag=1 and high_tumor_grade='1' then bc_high=1;
else if breast_cancer_diag=0 then bc_high=0;
else bc_high=.;
run;

proc freq data=mydata.cohort_final_1_A; table bc_high; run; /* OK */

proc freq data=mydata.cohort_final_1_A; table (statin testosterone
drug_cat)*bc_high; weight weight; run;

proc surveyphreg data=mydata.cohort_final_1_A;

class white(ref='0') black(ref='0') hisp(ref='0')

      diabetes(ref='0') hypertension(ref='0') wasting(ref='0')
malaise(ref='0') osteoporosis(ref='0') pituitary_dysf(ref='0')

      depression(ref='0') hyperlipidemia(ref='0') cardiovascular(ref='0')
insulin(ref='0') cci_cat(ref='0')  statin(ref='0')/param=ref;

strata match_id;

model timeB*bc_high(0)=age_exposed white black hisp diabetes hypertension
wasting malaise osteoporosis pituitary_dysf

            depression hyperlipidemia cardiovascular insulin cci_cat
visits_no education poverty breast_test_no /*col_test_no ov_test_no*/

            statin/risklimits;

weight weight;

run;
```

```
proc surveyphreg data=mydata.cohort_final_1_A;

class white(ref='0') black(ref='0') hisp(ref='0')

      diabetes(ref='0') hypertension(ref='0') wasting(ref='0')
malaise(ref='0') osteoporosis(ref='0') pituitary_dysf(ref='0')

      depression(ref='0') hyperlipidemia(ref='0') cardiovascular(ref='0')
insulin(ref='0') cci_cat(ref='0')  testosterone(ref='0')/param=ref;

strata match_id;

model timeB*bc_high(0)=age_exposed white black hisp diabetes hypertension
wasting malaise osteoporosis pituitary_dysf

            depression hyperlipidemia cardiovascular insulin cci_cat
visits_no education poverty breast_test_no /*col_test_no ov_test_no*/

            testosterone/risklimits;

weight weight;

run;



proc surveyphreg data=mydata.cohort_final_1_A;

class white(ref='0') black(ref='0') hisp(ref='0')

      diabetes(ref='0') hypertension(ref='0') wasting(ref='0')
malaise(ref='0') osteoporosis(ref='0') pituitary_dysf(ref='0')

      depression(ref='0') hyperlipidemia(ref='0') cardiovascular(ref='0')
insulin(ref='0') cci_cat(ref='0')  drug_cat(ref='None')/param=ref;

strata match_id;

model timeB*bc_high(0)=age_exposed white black hisp diabetes hypertension
wasting malaise osteoporosis pituitary_dysf

            depression hyperlipidemia cardiovascular insulin cci_cat
visits_no education poverty breast_test_no /*col_test_no ov_test_no*/

            drug_cat/risklimits;

weight weight;

run;

**Among colorectal cancer only;

title 'Among colorectal cancer only  - outcome=High grade cancer';

proc freq data=mydata.cohort_final_1_A; table
colorectal_cancer_diag*high_tumor_grade; run;
```

```
data mydata.cohort_final_1_A;
set mydata.cohort_final_1_A;
if colorectal_cancer_diag=1 and high_tumor_grade='1' then cc_high=1;
else if colorectal_cancer_diag=0 then cc_high=0;
else cc_high=.;
run;

proc freq data=mydata.cohort_final_1_A; table cc_high; run; /* OK */

proc freq data=mydata.cohort_final_1_A; table (statin testosterone
drug_cat)*cc_high; weight weight; run;

proc surveyphreg data=mydata.cohort_final_1_A;

class  /*high(ref='0')*/ white(ref='0') black(ref='0') hisp(ref='0')

      diabetes(ref='0') hypertension(ref='0') wasting(ref='0')
malaise(ref='0') osteoporosis(ref='0') pituitary_dysf(ref='0')

      depression(ref='0') hyperlipidemia(ref='0') cardiovascular(ref='0')
insulin(ref='0') cci_cat(ref='0')  statin(ref='0')/param=ref;

strata match_id;

model timeB*cc_high(0)=age_exposed white black hisp diabetes hypertension
wasting malaise osteoporosis pituitary_dysf

            depression hyperlipidemia cardiovascular insulin cci_cat
visits_no education poverty /*breast_test_no*/ col_test_no /*ov_test_no*/

            statin/risklimits;

weight weight;

run;


proc surveyphreg data=mydata.cohort_final_1_A;

class  /*high(ref='0')*/ white(ref='0') black(ref='0') hisp(ref='0')

      diabetes(ref='0') hypertension(ref='0') wasting(ref='0')
malaise(ref='0') osteoporosis(ref='0') pituitary_dysf(ref='0')

      depression(ref='0') hyperlipidemia(ref='0') cardiovascular(ref='0')
insulin(ref='0') cci_cat(ref='0')  testosterone(ref='0')/param=ref;

strata match_id;

model timeB*cc_high(0)=age_exposed white black hisp diabetes hypertension
wasting malaise osteoporosis pituitary_dysf

            depression hyperlipidemia cardiovascular insulin cci_cat
visits_no education poverty /*breast_test_no*/ col_test_no /*ov_test_no*/

            testosterone/risklimits;
```

```
weight weight;

run;


proc surveyphreg data=mydata.cohort_final_1_A;

class  /*high(ref='0')*/ white(ref='0') black(ref='0') hisp(ref='0')

      diabetes(ref='0') hypertension(ref='0') wasting(ref='0')
malaise(ref='0') osteoporosis(ref='0') pituitary_dysf(ref='0')

      depression(ref='0') hyperlipidemia(ref='0') cardiovascular(ref='0')
insulin(ref='0') cci_cat(ref='0')  drug_cat(ref='None')/param=ref;

strata match_id;

model timeB*cc_high(0)=age_exposed white black hisp diabetes hypertension
wasting malaise osteoporosis pituitary_dysf

            depression hyperlipidemia cardiovascular insulin cci_cat
visits_no education poverty /*breast_test_no*/ col_test_no /*ov_test_no*/

            drug_cat/risklimits;

weight weight;

run;

**Among ovarian cancer only;

title 'Among ovarian cancer only  - outcome=High grade cancer';

proc freq data=mydata.cohort_final_1_A; table
ovarian_cancer_diag*high_tumor_grade; run;

data mydata.cohort_final_1_A;
set mydata.cohort_final_1_A;
if ovarian_cancer_diag=1 and high_tumor_grade='1' then oc_high=1;
else if ovarian_cancer_diag=0 then oc_high=0;
else oc_high=.;
run;

proc freq data=mydata.cohort_final_1_A; table oc_high; run; /* OK */

proc freq data=mydata.cohort_final_1_A; table (statin testosterone
drug_cat)*oc_high; weight weight; run;

proc surveyphreg data=mydata.cohort_final_1_A;

class  white(ref='0') black(ref='0') hisp(ref='0')

      diabetes(ref='0') hypertension(ref='0') wasting(ref='0')
malaise(ref='0') osteoporosis(ref='0') pituitary_dysf(ref='0')
```

```
      depression(ref='0') hyperlipidemia(ref='0') cardiovascular(ref='0')
insulin(ref='0') cci_cat(ref='0')  statin(ref='0')/param=ref;

strata match_id;

model timeB*oc_high(0)=age_exposed white black hisp diabetes hypertension
wasting malaise osteoporosis pituitary_dysf

            depression hyperlipidemia cardiovascular insulin cci_cat
visits_no education poverty /*breast_test_no col_test_no*/ ov_test_no

            statin/risklimits;

weight weight;

run;


proc surveyphreg data=mydata.cohort_final_1_A;

class  white(ref='0') black(ref='0') hisp(ref='0')

      diabetes(ref='0') hypertension(ref='0') wasting(ref='0')
malaise(ref='0') osteoporosis(ref='0') pituitary_dysf(ref='0')

      depression(ref='0') hyperlipidemia(ref='0') cardiovascular(ref='0')
insulin(ref='0') cci_cat(ref='0')  testosterone(ref='0')/param=ref;

strata match_id;

model timeB*oc_high(0)=age_exposed white black hisp diabetes hypertension
wasting malaise osteoporosis pituitary_dysf

            depression hyperlipidemia cardiovascular insulin cci_cat
visits_no education poverty /*breast_test_no col_test_no*/ ov_test_no

            testosterone/risklimits;

weight weight;

run;



proc surveyphreg data=mydata.cohort_final_1_A;

class  white(ref='0') black(ref='0') hisp(ref='0')

      diabetes(ref='0') hypertension(ref='0') wasting(ref='0')
malaise(ref='0') osteoporosis(ref='0') pituitary_dysf(ref='0')

      depression(ref='0') hyperlipidemia(ref='0') cardiovascular(ref='0')
insulin(ref='0') cci_cat(ref='0')  drug_cat(ref='None')/param=ref;

strata match_id;
```

```
model timeB*oc_high(0)=age_exposed white black hisp diabetes hypertension
wasting malaise osteoporosis pituitary_dysf

            depression hyperlipidemia cardiovascular insulin cci_cat
visits_no education poverty /*breast_test_no col_test_no*/ ov_test_no

            drug_cat/risklimits;

weight weight;

run;

**Among endometrial cancer only;

title 'Among endometrial cancer only  - outcome=High grade cancer';

proc freq data=mydata.cohort_final_1_A; table
endo_cancer_diag*high_tumor_grade; run;

data mydata.cohort_final_1_A;
set mydata.cohort_final_1_A;
if endo_cancer_diag=1 and high_tumor_grade='1' then ec_high=1;
else if endo_cancer_diag=0 then ec_high=0;
else ec_high=.;
run;

proc freq data=mydata.cohort_final_1_A; table ec_high; run; /* OK */

proc freq data=mydata.cohort_final_1_A; table (statin testosterone
drug_cat)*ec_high; weight weight; run;

proc surveyphreg data=mydata.cohort_final_1_A;

class  /*high(ref='0')*/ white(ref='0') black(ref='0') hisp(ref='0')

      diabetes(ref='0') hypertension(ref='0') wasting(ref='0')
malaise(ref='0') osteoporosis(ref='0') pituitary_dysf(ref='0')

      depression(ref='0') hyperlipidemia(ref='0') cardiovascular(ref='0')
insulin(ref='0') cci_cat(ref='0')  statin(ref='0')/param=ref;

strata match_id;

model timeB*ec_high(0)=age_exposed white black hisp diabetes hypertension
wasting malaise osteoporosis pituitary_dysf

            depression hyperlipidemia cardiovascular insulin cci_cat
visits_no education poverty /*breast_test_no col_test_no ov_test_no*/

            statin/risklimits;

weight weight;

run;


proc surveyphreg data=mydata.cohort_final_1_A;
```

```
class  /*high(ref='0')*/ white(ref='0') black(ref='0') hisp(ref='0')

      diabetes(ref='0') hypertension(ref='0') wasting(ref='0')
malaise(ref='0') osteoporosis(ref='0') pituitary_dysf(ref='0')

      depression(ref='0') hyperlipidemia(ref='0') cardiovascular(ref='0')
insulin(ref='0') cci_cat(ref='0')  testosterone(ref='0')/param=ref;

strata match_id;

model timeB*ec_high(0)=age_exposed white black hisp diabetes hypertension
wasting malaise osteoporosis pituitary_dysf

            depression hyperlipidemia cardiovascular insulin cci_cat
visits_no education poverty /*breast_test_no col_test_no ov_test_no*/

            testosterone/risklimits;

weight weight;

run;



proc surveyphreg data=mydata.cohort_final_1_A;

class  /*high(ref='0')*/ white(ref='0') black(ref='0') hisp(ref='0')

      diabetes(ref='0') hypertension(ref='0') wasting(ref='0')
malaise(ref='0') osteoporosis(ref='0') pituitary_dysf(ref='0')

      depression(ref='0') hyperlipidemia(ref='0') cardiovascular(ref='0')
insulin(ref='0') cci_cat(ref='0')  drug_cat(ref='None')/param=ref;

strata match_id;

model timeB*ec_high(0)=age_exposed white black hisp diabetes hypertension
wasting malaise osteoporosis pituitary_dysf

            depression hyperlipidemia cardiovascular insulin cci_cat
visits_no education poverty /*breast_test_no col_test_no ov_test_no*/

            drug_cat/risklimits;

weight weight;

run;




/*3-Outcome: advanced-stage HRCs versus non-cancer cases */

title 'All cancers (breast, colorectal, ovarian, endometrial) -
outcome=Advanced stage cancer';
```

```
proc freq data=mydata.cohort_final_1_A; table
cancer_diagnosed*advanced_cancer_stage; run;

data mydata.cohort_final_1_A;
set mydata.cohort_final_1_A;
if cancer_diagnosed='1' and advanced_cancer_stage='1' then advanced='1';
*Advanced-stage cancer among cancer patients only, exclude cancer  patients
with non advanced stage;
if cancer_diagnosed='0' then advanced='0'; *Non-cancer patients, no existing
advanced stage;
run;
/* NOTE: The data set MYDATA.COHORT_FINAL_1_A has 142772 observations and 108
variables. */

proc freq data=mydata.cohort_final_1_A; table advanced; run; /* OK */

proc freq data=mydata.cohort_final_1_A; table (statin testosterone
drug_cat)*advanced; weight weight; run; /* OK */


/* Create time variable */
data mydata.cohort_final_1_A;
set mydata.cohort_final_1_A;
if advanced='1' then timeC=dx_date-index_dt_new; else timeC="31DEC2016"D-
index_dt_new;
advanced_new=advanced*1; *Numeric version of the variable;
run;

proc freq data=mydata.cohort_final_1_A; table timeC; run; /* OK, min ttime is
6 months. */

proc surveyphreg data=mydata.cohort_final_1_A;

class white(ref='0') black(ref='0') hisp(ref='0')

      diabetes(ref='0') hypertension(ref='0') wasting(ref='0')
malaise(ref='0') osteoporosis(ref='0') pituitary_dysf(ref='0')

      depression(ref='0') hyperlipidemia(ref='0') cardiovascular(ref='0')
insulin(ref='0') cci_cat(ref='0')  statin(ref='0')/param=ref;

strata match_id;

model timeC*advanced_new(0)=age_exposed white black hisp diabetes
hypertension wasting malaise osteoporosis pituitary_dysf

            depression hyperlipidemia cardiovascular insulin cci_cat
visits_no education poverty breast_test_no col_test_no ov_test_no

            statin/risklimits;

weight weight;

run;


proc surveyphreg data=mydata.cohort_final_1_A;
```

```
class white(ref='0') black(ref='0') hisp(ref='0')

      diabetes(ref='0') hypertension(ref='0') wasting(ref='0')
malaise(ref='0') osteoporosis(ref='0') pituitary_dysf(ref='0')

      depression(ref='0') hyperlipidemia(ref='0') cardiovascular(ref='0')
insulin(ref='0') cci_cat(ref='0')  testosterone(ref='0')/param=ref;

strata match_id;

model timeC*advanced_new(0)=age_exposed white black hisp diabetes
hypertension wasting malaise osteoporosis pituitary_dysf

            depression hyperlipidemia cardiovascular insulin cci_cat
visits_no education poverty breast_test_no col_test_no ov_test_no

            testosterone/risklimits;

weight weight;

run;


proc surveyphreg data=mydata.cohort_final_1_A;

class white(ref='0') black(ref='0') hisp(ref='0')

      diabetes(ref='0') hypertension(ref='0') wasting(ref='0')
malaise(ref='0') osteoporosis(ref='0') pituitary_dysf(ref='0')

      depression(ref='0') hyperlipidemia(ref='0') cardiovascular(ref='0')
insulin(ref='0') cci_cat(ref='0')  drug_cat(ref='None')/param=ref;

strata match_id;

model timeC*advanced_new(0)=age_exposed white black hisp diabetes
hypertension wasting malaise osteoporosis pituitary_dysf

            depression hyperlipidemia cardiovascular insulin cci_cat
visits_no education poverty breast_test_no col_test_no ov_test_no

            drug_cat/risklimits;

weight weight;

run;

**Among breast cancer only;

title 'Among breast cancer only - outcome=Advanced stage cancer';

proc freq data=mydata.cohort_final_1_A; table
breast_cancer_diag*advanced_cancer_stage; run;

data mydata.cohort_final_1_A;
set mydata.cohort_final_1_A;
```

```
if breast_cancer_diag='1' and advanced_cancer_stage='1' then bc_advanced=1;
else if breast_cancer_diag='0' then bc_advanced=0;
else bc_advanced=.;
run;


proc freq data=mydata.cohort_final_1_A; table bc_advanced; run;

proc freq data=mydata.cohort_final_1_A; table (statin testosterone
drug_cat)*bc_advanced; weight weight; run; /* OK */

proc surveyphreg data=mydata.cohort_final_1_A;

class /*advanced(ref='0')*/ white(ref='0') black(ref='0') hisp(ref='0')

      diabetes(ref='0') hypertension(ref='0') wasting(ref='0')
malaise(ref='0') osteoporosis(ref='0') pituitary_dysf(ref='0')

      depression(ref='0') hyperlipidemia(ref='0') cardiovascular(ref='0')
insulin(ref='0') cci_cat(ref='0')  statin(ref='0')/param=ref;

strata match_id;

model timeC*bc_advanced(0)=age_exposed white black hisp diabetes hypertension
wasting malaise osteoporosis pituitary_dysf

            depression hyperlipidemia cardiovascular insulin cci_cat
visits_no education poverty breast_test_no /*col_test_no ov_test_no*/

            statin/risklimits;

weight weight;

run;


proc surveyphreg data=mydata.cohort_final_1_A;

class /*advanced(ref='0')*/ white(ref='0') black(ref='0') hisp(ref='0')

      diabetes(ref='0') hypertension(ref='0') wasting(ref='0')
malaise(ref='0') osteoporosis(ref='0') pituitary_dysf(ref='0')

      depression(ref='0') hyperlipidemia(ref='0') cardiovascular(ref='0')
insulin(ref='0') cci_cat(ref='0')  testosterone(ref='0')/param=ref;

strata match_id;

model timeC*bc_advanced(0)=age_exposed white black hisp diabetes hypertension
wasting malaise osteoporosis pituitary_dysf

            depression hyperlipidemia cardiovascular insulin cci_cat
visits_no education poverty breast_test_no /*col_test_no ov_test_no*/

            testosterone/risklimits;

weight weight;
```

```
run;

proc surveyphreg data=mydata.cohort_final_1_A;

class /*advanced(ref='0')*/ white(ref='0') black(ref='0') hisp(ref='0')

      diabetes(ref='0') hypertension(ref='0') wasting(ref='0')
malaise(ref='0') osteoporosis(ref='0') pituitary_dysf(ref='0')

      depression(ref='0') hyperlipidemia(ref='0') cardiovascular(ref='0')
insulin(ref='0') cci_cat(ref='0')  drug_cat(ref='None')/param=ref;

strata match_id;

model timeC*bc_advanced(0)=age_exposed white black hisp diabetes hypertension
wasting malaise osteoporosis pituitary_dysf

            depression hyperlipidemia cardiovascular insulin cci_cat
visits_no education poverty breast_test_no /*col_test_no ov_test_no*/

            drug_cat/risklimits;

weight weight;

run;


**Among colorectal cancer only;

title 'Among colorectal cancer only - outcome=Advanced stage cancer';

proc freq data=mydata.cohort_final_1_A; table
colorectal_cancer_diag*advanced_cancer_stage; run;

data mydata.cohort_final_1_A;
set mydata.cohort_final_1_A;
if colorectal_cancer_diag='1' and advanced_cancer_stage='1' then
cc_advanced=1;
else if colorectal_cancer_diag='0' then cc_advanced=0;
else cc_advanced=.;
run;


proc freq data=mydata.cohort_final_1_A; table cc_advanced; run;

proc freq data=mydata.cohort_final_1_A; table (statin testosterone
drug_cat)*cc_advanced; weight weight; run; /* OK */

proc surveyphreg data=mydata.cohort_final_1_A;

class white(ref='0') black(ref='0') hisp(ref='0')

      diabetes(ref='0') hypertension(ref='0') wasting(ref='0')
malaise(ref='0') osteoporosis(ref='0') pituitary_dysf(ref='0')
```

```
	depression(ref='0') hyperlipidemia(ref='0') cardiovascular(ref='0') 
insulin(ref='0') cci_cat(ref='0')  statin(ref='0')/param=ref;

strata match_id;

model timeC*cc_advanced(0)=age_exposed white black hisp diabetes hypertension 
wasting malaise osteoporosis pituitary_dysf

		depression hyperlipidemia cardiovascular insulin cci_cat 
visits_no education poverty /*breast_test_no*/ col_test_no /*ov_test_no*/

		statin/risklimits;

weight weight;

run;

proc surveyphreg data=mydata.cohort_final_1_A;

class white(ref='0') black(ref='0') hisp(ref='0')

	diabetes(ref='0') hypertension(ref='0') wasting(ref='0') 
malaise(ref='0') osteoporosis(ref='0') pituitary_dysf(ref='0')

	depression(ref='0') hyperlipidemia(ref='0') cardiovascular(ref='0') 
insulin(ref='0') cci_cat(ref='0')  testosterone(ref='0')/param=ref;

strata match_id;

model timeC*cc_advanced(0)=age_exposed white black hisp diabetes hypertension 
wasting malaise osteoporosis pituitary_dysf

		depression hyperlipidemia cardiovascular insulin cci_cat 
visits_no education poverty /*breast_test_no*/ col_test_no /*ov_test_no*/

		testosterone/risklimits;

weight weight;

run;


proc surveyphreg data=mydata.cohort_final_1_A;

class white(ref='0') black(ref='0') hisp(ref='0')

	diabetes(ref='0') hypertension(ref='0') wasting(ref='0') 
malaise(ref='0') osteoporosis(ref='0') pituitary_dysf(ref='0')

	depression(ref='0') hyperlipidemia(ref='0') cardiovascular(ref='0') 
insulin(ref='0') cci_cat(ref='0')  drug_cat(ref='None')/param=ref;

strata match_id;

model timeC*cc_advanced(0)=age_exposed white black hisp diabetes hypertension 
wasting malaise osteoporosis pituitary_dysf
```

```
            depression hyperlipidemia cardiovascular insulin cci_cat
visits_no education poverty /*breast_test_no*/ col_test_no /*ov_test_no*/

            drug_cat/risklimits;

weight weight;

run;






**Among ovarian cancer only;

title 'Among ovarian cancer only - outcome=Advanced stage cancer';

proc freq data=mydata.cohort_final_1_A; table
ovarian_cancer_diag*advanced_cancer_stage; run;

data mydata.cohort_final_1_A;
set mydata.cohort_final_1_A;
if ovarian_cancer_diag='1' and advanced_cancer_stage='1' then oc_advanced=1;
else if ovarian_cancer_diag='0' then oc_advanced=0;
else cc_advanced=.;
run;


proc freq data=mydata.cohort_final_1_A; table oc_advanced; run;

proc freq data=mydata.cohort_final_1_A; table (statin testosterone
drug_cat)*oc_advanced; weight weight; run; /* OK */

proc surveyphreg data=mydata.cohort_final_1_A;

class white(ref='0') black(ref='0') hisp(ref='0')

      diabetes(ref='0') hypertension(ref='0') wasting(ref='0')
malaise(ref='0') osteoporosis(ref='0') pituitary_dysf(ref='0')

      depression(ref='0') hyperlipidemia(ref='0') cardiovascular(ref='0')
insulin(ref='0') cci_cat(ref='0')  statin(ref='0')/param=ref;

strata match_id;

model timeC*oc_advanced(0)=age_exposed white black hisp diabetes hypertension
wasting malaise osteoporosis pituitary_dysf

            depression hyperlipidemia cardiovascular insulin cci_cat
visits_no education poverty /*breast_test_no*/ col_test_no /*ov_test_no*/

            statin/risklimits;

weight weight;
```

```
run;


proc surveyphreg data=mydata.cohort_final_1_A;

class white(ref='0') black(ref='0') hisp(ref='0')

      diabetes(ref='0') hypertension(ref='0') wasting(ref='0')
malaise(ref='0') osteoporosis(ref='0') pituitary_dysf(ref='0')

      depression(ref='0') hyperlipidemia(ref='0') cardiovascular(ref='0')
insulin(ref='0') cci_cat(ref='0')  testosterone(ref='0')/param=ref;

strata match_id;

model timeC*oc_advanced(0)=age_exposed white black hisp diabetes hypertension
wasting malaise osteoporosis pituitary_dysf

            depression hyperlipidemia cardiovascular insulin cci_cat
visits_no education poverty /*breast_test_no*/ col_test_no /*ov_test_no*/

            testosterone/risklimits;

weight weight;

run;


proc surveyphreg data=mydata.cohort_final_1_A;

class white(ref='0') black(ref='0') hisp(ref='0')

      diabetes(ref='0') hypertension(ref='0') wasting(ref='0')
malaise(ref='0') osteoporosis(ref='0') pituitary_dysf(ref='0')

      depression(ref='0') hyperlipidemia(ref='0') cardiovascular(ref='0')
insulin(ref='0') cci_cat(ref='0')  drug_cat(ref='None')/param=ref;

strata match_id;

model timeC*oc_advanced(0)=age_exposed white black hisp diabetes hypertension
wasting malaise osteoporosis pituitary_dysf

            depression hyperlipidemia cardiovascular insulin cci_cat
visits_no education poverty /*breast_test_no*/ col_test_no /*ov_test_no*/

            drug_cat/risklimits;

weight weight;

run;


**Among endometrian cancer only;

title 'Among endometrial cancer only - outcome=Advanced stage cancer';
```

```
proc freq data=mydata.cohort_final_1_A; table
endo_cancer_diag*advanced_cancer_stage; run;

data mydata.cohort_final_1_A;
set mydata.cohort_final_1_A;
if endo_cancer_diag='1' and advanced_cancer_stage='1' then ec_advanced=1;
else if endo_cancer_diag='0' then ec_advanced=0;
else ec_advanced=.;
run;


proc freq data=mydata.cohort_final_1_A; table ec_advanced; run;

proc freq data=mydata.cohort_final_1_A; table (statin testosterone
drug_cat)*ec_advanced; weight weight; run; /* OK */


proc surveyphreg data=mydata.cohort_final_1_A;

class white(ref='0') black(ref='0') hisp(ref='0')

      diabetes(ref='0') hypertension(ref='0') wasting(ref='0')
malaise(ref='0') osteoporosis(ref='0') pituitary_dysf(ref='0')

      depression(ref='0') hyperlipidemia(ref='0') cardiovascular(ref='0')
insulin(ref='0') cci_cat(ref='0')  statin(ref='0')/param=ref;

strata match_id;

model timeC*ec_advanced(0)=age_exposed white black hisp diabetes hypertension
wasting malaise osteoporosis pituitary_dysf

            depression hyperlipidemia cardiovascular insulin cci_cat
visits_no education poverty /*breast_test_no col_test_no ov_test_no*/

           statin/risklimits;

weight weight;

run;



proc surveyphreg data=mydata.cohort_final_1_A;

class white(ref='0') black(ref='0') hisp(ref='0')

      diabetes(ref='0') hypertension(ref='0') wasting(ref='0')
malaise(ref='0') osteoporosis(ref='0') pituitary_dysf(ref='0')

      depression(ref='0') hyperlipidemia(ref='0') cardiovascular(ref='0')
insulin(ref='0') cci_cat(ref='0')  testosterone(ref='0')/param=ref;

strata match_id;

model timeC*ec_advanced(0)=age_exposed white black hisp diabetes hypertension
wasting malaise osteoporosis pituitary_dysf
```

```
            depression hyperlipidemia cardiovascular insulin cci_cat
visits_no education poverty /*breast_test_no col_test_no ov_test_no*/

           testosterone/risklimits;

weight weight;

run;


proc surveyphreg data=mydata.cohort_final_1_A;

class white(ref='0') black(ref='0') hisp(ref='0')

      diabetes(ref='0') hypertension(ref='0') wasting(ref='0')
malaise(ref='0') osteoporosis(ref='0') pituitary_dysf(ref='0')

      depression(ref='0') hyperlipidemia(ref='0') cardiovascular(ref='0')
insulin(ref='0') cci_cat(ref='0')  drug_cat(ref='None')/param=ref;

strata match_id;

model timeC*ec_advanced(0)=age_exposed white black hisp diabetes hypertension
wasting malaise osteoporosis pituitary_dysf

            depression hyperlipidemia cardiovascular insulin cci_cat
visits_no education poverty /*breast_test_no col_test_no ov_test_no*/

           drug_cat/risklimits;

weight weight;

run;


/*4-Outcome=HRC mortality vs non-HRC cases alive */

title 'All cancers (breast, colorectal, ovarian, endometrial) - outcome=HRC
mortality';

proc freq data=mydata.cohort_final_1_A; table cancer_diagnosed*death; run;

data mydata.cohort_final_1_A;
set mydata.cohort_final_1_A;

death_new=death*1; *Numeric version;

if death='1' and cancer_diagnosed='1' then cancer_mortality=1; else
cancer_mortality=0; ***Cancer and death;

if high_tumor_grade='1' then high_hrc='1'; else high_hrc='0';

if advanced_cancer_stage='1' then stage_hrc='1'; else stage_hrc='0';

run;
/*
```

```
if death='1' then do;
if cancer_diagnosed='1' then cancer_mortality=1; ***Cancer and death;
if cancer_diagnosed='0' then cancer_mortality=2; **Death but not related to
cancer (censored);
end;

if death='0' and cancer_diagnosed='0' then cancer_mortality=3; **Non-cancer
mortality;
*/



proc freq data=mydata.cohort_final_1_A; tables cancer_mortality; run; /* OK
*/

proc freq data=mydata.cohort_final_1_A; tables
high_tumor_grade*cancer_diagnosed high_hrc
advanced_cancer_stage*cancer_diagnosed stage_hrc; run; /* OK */

proc freq data=mydata.cohort_final_1_A; table dod; run;

proc freq data=mydata.cohort_final_1_A; table index_dt_new; run;



/* Create time variable */

data mydata.cohort_final_1_A;
set mydata.cohort_final_1_A;
if cancer_mortality=1 then timeD=mdy(month(dod),01,year(dod))-index_dt_new;
else timeD="31DEC2016"D-index_dt_new;
run;
/* NOTE: The data set MYDATA.COHORT_FINAL_1_A has 142772 observations and 119
variables. */


proc freq data=mydata.cohort_final_1_A; table timeD; run;
**All cancer;

proc freq data=mydata.cohort_final_1_A; table (statin testosterone
drug_cat)*cancer_mortality; weight weight; run; /* OK */


proc surveyphreg data=mydata.cohort_final_1_A;

class  white(ref='0') black(ref='0') hisp(ref='0')

      high_hrc(ref='0') stage_hrc(ref='0')

      diabetes(ref='0') hypertension(ref='0') wasting(ref='0')
malaise(ref='0') osteoporosis(ref='0') pituitary_dysf(ref='0')

      depression(ref='0') hyperlipidemia(ref='0') cardiovascular(ref='0')
insulin(ref='0') cci_cat(ref='0') statin(ref='0')/param=ref;

strata match_id;
```

```
model timeD*cancer_mortality(0)=age_exposed white black hisp diabetes
high_hrc stage_hrc

            hypertension wasting malaise osteoporosis pituitary_dysf

            depression hyperlipidemia cardiovascular insulin cci_cat
visits_no education poverty breast_test_no col_test_no ov_test_no

            statin/risklimits;

weight weight;

run;


proc surveyphreg data=mydata.cohort_final_1_A;

class  white(ref='0') black(ref='0') hisp(ref='0')

      high_hrc(ref='0') stage_hrc(ref='0')

      diabetes(ref='0') hypertension(ref='0') wasting(ref='0')
malaise(ref='0') osteoporosis(ref='0') pituitary_dysf(ref='0')

      depression(ref='0') hyperlipidemia(ref='0') cardiovascular(ref='0')
insulin(ref='0') cci_cat(ref='0') testosterone(ref='0')/param=ref;

strata match_id;

model timeD*cancer_mortality(0)=age_exposed white black hisp diabetes
high_hrc stage_hrc

            hypertension wasting malaise osteoporosis pituitary_dysf

            depression hyperlipidemia cardiovascular insulin cci_cat
visits_no education poverty breast_test_no col_test_no ov_test_no

            testosterone/risklimits;

weight weight;

run;


proc surveyphreg data=mydata.cohort_final_1_A;

class  white(ref='0') black(ref='0') hisp(ref='0')

      high_hrc(ref='0') stage_hrc(ref='0')

      diabetes(ref='0') hypertension(ref='0') wasting(ref='0')
malaise(ref='0') osteoporosis(ref='0') pituitary_dysf(ref='0')

      depression(ref='0') hyperlipidemia(ref='0') cardiovascular(ref='0')
insulin(ref='0') cci_cat(ref='0') drug_cat(ref='None')/param=ref;

strata match_id;
```

```
model timeD*cancer_mortality(0)=age_exposed white black hisp diabetes
high_hrc stage_hrc

            hypertension wasting malaise osteoporosis pituitary_dysf

            depression hyperlipidemia cardiovascular insulin cci_cat
visits_no education poverty breast_test_no col_test_no ov_test_no

            drug_cat/risklimits;

weight weight;

run;




**Among breast cancer only;

title 'Among breast cancer only - outcome=HRC mortality';

proc freq data=mydata.cohort_final_1_A; table
breast_cancer_diag*cancer_mortality; run; /* OK */

data mydata.cohort_final_1_A;
set mydata.cohort_final_1_A;
if  breast_cancer_diag='1' and cancer_mortality=1 then bc_mortality=1;
***Breast cancer and death;
else bc_mortality=0;
run;


proc freq data=mydata.cohort_final_1_A; table bc_mortality; run; /* OK */

proc freq data=mydata.cohort_final_1_A; table (statin testosterone
drug_cat)*bc_mortality; weight weight; run; /* OK */

proc surveyphreg data=mydata.cohort_final_1_A;

class  white(ref='0') black(ref='0') hisp(ref='0')

      high_hrc(ref='0') stage_hrc(ref='0')

      diabetes(ref='0') hypertension(ref='0') wasting(ref='0')
malaise(ref='0') osteoporosis(ref='0') pituitary_dysf(ref='0')

      depression(ref='0') hyperlipidemia(ref='0') cardiovascular(ref='0')
insulin(ref='0') cci_cat(ref='0') statin(ref='0')/param=ref;

strata match_id;

model timeD*bc_mortality(0)=age_exposed white black hisp diabetes high_hrc
stage_hrc

            hypertension wasting malaise osteoporosis pituitary_dysf
```

```
            depression hyperlipidemia cardiovascular insulin cci_cat
visits_no education poverty breast_test_no /*col_test_no ov_test_no*/

            statin/risklimits;

weight weight;

run;


proc surveyphreg data=mydata.cohort_final_1_A;

class  white(ref='0') black(ref='0') hisp(ref='0')

      high_hrc(ref='0') stage_hrc(ref='0')

      diabetes(ref='0') hypertension(ref='0') wasting(ref='0')
malaise(ref='0') osteoporosis(ref='0') pituitary_dysf(ref='0')

      depression(ref='0') hyperlipidemia(ref='0') cardiovascular(ref='0')
insulin(ref='0') cci_cat(ref='0') testosterone(ref='0')/param=ref;

strata match_id;

model timeD*bc_mortality(0)=age_exposed white black hisp diabetes high_hrc
stage_hrc

            hypertension wasting malaise osteoporosis pituitary_dysf

            depression hyperlipidemia cardiovascular insulin cci_cat
visits_no education poverty breast_test_no /*col_test_no ov_test_no*/

            testosterone/risklimits;

weight weight;

run;


proc surveyphreg data=mydata.cohort_final_1_A;

class  white(ref='0') black(ref='0') hisp(ref='0')

      high_hrc(ref='0') stage_hrc(ref='0')

      diabetes(ref='0') hypertension(ref='0') wasting(ref='0')
malaise(ref='0') osteoporosis(ref='0') pituitary_dysf(ref='0')

      depression(ref='0') hyperlipidemia(ref='0') cardiovascular(ref='0')
insulin(ref='0') cci_cat(ref='0') drug_cat(ref='None')/param=ref;

strata match_id;

model timeD*bc_mortality(0)=age_exposed white black hisp diabetes high_hrc
stage_hrc

            hypertension wasting malaise osteoporosis pituitary_dysf
```

```
            depression hyperlipidemia cardiovascular insulin cci_cat
visits_no education poverty breast_test_no /*col_test_no ov_test_no*/

            drug_cat/risklimits;

weight weight;

run;


**Among colorectal cancer only;

title 'Among colorectal cancer only - outcome= HRC mortality';

proc freq data=mydata.cohort_final_1_A; table
colorectal_cancer_diag*cancer_mortality; run; /* OK */

data mydata.cohort_final_1_A;
set mydata.cohort_final_1_A;
if  colorectal_cancer_diag='1' and cancer_mortality=1 then cc_mortality=1;
***Colorectal cancer and death;
else cc_mortality=0;
run;


proc freq data=mydata.cohort_final_1_A; table cc_mortality; run; /* OK */

proc freq data=mydata.cohort_final_1_A; table (statin testosterone
drug_cat)*cc_mortality; weight weight; run; /* OK */


proc surveyphreg data=mydata.cohort_final_1_A;

class  white(ref='0') black(ref='0') hisp(ref='0')

      high_hrc(ref='0') stage_hrc(ref='0')

      diabetes(ref='0') hypertension(ref='0') wasting(ref='0')
malaise(ref='0') osteoporosis(ref='0') pituitary_dysf(ref='0')

      depression(ref='0') hyperlipidemia(ref='0') cardiovascular(ref='0')
insulin(ref='0') cci_cat(ref='0') statin(ref='0')/param=ref;

strata match_id;

model timeD*cc_mortality(0)=age_exposed white black hisp diabetes high_hrc
stage_hrc

            hypertension wasting malaise osteoporosis pituitary_dysf

            depression hyperlipidemia cardiovascular insulin cci_cat
visits_no education poverty /*breast_test_no*/ col_test_no /*ov_test_no*/

            statin/risklimits;

weight weight;
```

```
run;

proc surveyphreg data=mydata.cohort_final_1_A;
class  white(ref='0') black(ref='0') hisp(ref='0')
      high_hrc(ref='0') stage_hrc(ref='0')
      diabetes(ref='0') hypertension(ref='0') wasting(ref='0')
malaise(ref='0') osteoporosis(ref='0') pituitary_dysf(ref='0')
      depression(ref='0') hyperlipidemia(ref='0') cardiovascular(ref='0')
insulin(ref='0') cci_cat(ref='0') testosterone(ref='0')/param=ref;
strata match_id;
model timeD*cc_mortality(0)=age_exposed white black hisp diabetes high_hrc
stage_hrc
            hypertension wasting malaise osteoporosis pituitary_dysf
            depression hyperlipidemia cardiovascular insulin cci_cat
visits_no education poverty /*breast_test_no*/ col_test_no /*ov_test_no*/
            testosterone/risklimits;
weight weight;
run;

proc surveyphreg data=mydata.cohort_final_1_A;
class  white(ref='0') black(ref='0') hisp(ref='0')
      high_hrc(ref='0') stage_hrc(ref='0')
      diabetes(ref='0') hypertension(ref='0') wasting(ref='0')
malaise(ref='0') osteoporosis(ref='0') pituitary_dysf(ref='0')
      depression(ref='0') hyperlipidemia(ref='0') cardiovascular(ref='0')
insulin(ref='0') cci_cat(ref='0') drug_cat(ref='None')/param=ref;
strata match_id;
model timeD*cc_mortality(0)=age_exposed white black hisp diabetes high_hrc
stage_hrc
            hypertension wasting malaise osteoporosis pituitary_dysf
            depression hyperlipidemia cardiovascular insulin cci_cat
visits_no education poverty /*breast_test_no*/ col_test_no /*ov_test_no*/
            drug_cat/risklimits;
```

```
weight weight;

run;




**Among ovarian cancer only;

title 'Among ovarian cancer only - outcome= HRC mortality';

proc freq data=mydata.cohort_final_1_A; table
ovarian_cancer_diag*cancer_mortality; run; /* OK */

data mydata.cohort_final_1_A;
set mydata.cohort_final_1_A;
if  ovarian_cancer_diag='1' and cancer_mortality='1' then oc_mortality=1;
***Ovarian cancer and death;
else oc_mortality=0;
run;


proc freq data=mydata.cohort_final_1_A; table oc_mortality; run; /* OK */

proc freq data=mydata.cohort_final_1_A; table (statin testosterone
drug_cat)*oc_mortality; weight weight; run; /* OK */

proc surveyphreg data=mydata.cohort_final_1_A;

class  white(ref='0') black(ref='0') hisp(ref='0')

      high_hrc(ref='0') stage_hrc(ref='0')

      diabetes(ref='0') hypertension(ref='0') wasting(ref='0')
malaise(ref='0') osteoporosis(ref='0') pituitary_dysf(ref='0')

      depression(ref='0') hyperlipidemia(ref='0') cardiovascular(ref='0')
insulin(ref='0') cci_cat(ref='0') statin(ref='0')/param=ref;

strata match_id;

model timeD*oc_mortality(0)=age_exposed white black hisp diabetes high_hrc
stage_hrc

            hypertension wasting malaise osteoporosis pituitary_dysf

            depression hyperlipidemia cardiovascular insulin cci_cat
visits_no education poverty /*breast_test_no col_test_no*/ ov_test_no

            statin/risklimits;

weight weight;

run;
```

```
proc surveyphreg data=mydata.cohort_final_1_A;

class  white(ref='0') black(ref='0') hisp(ref='0')

      high_hrc(ref='0') stage_hrc(ref='0')

      diabetes(ref='0') hypertension(ref='0') wasting(ref='0')
malaise(ref='0') osteoporosis(ref='0') pituitary_dysf(ref='0')

      depression(ref='0') hyperlipidemia(ref='0') cardiovascular(ref='0')
insulin(ref='0') cci_cat(ref='0') testosterone(ref='0')/param=ref;

strata match_id;

model timeD*oc_mortality(0)=age_exposed white black hisp diabetes high_hrc
stage_hrc

            hypertension wasting malaise osteoporosis pituitary_dysf

            depression hyperlipidemia cardiovascular insulin cci_cat
visits_no education poverty /*breast_test_no col_test_no*/ ov_test_no

            testosterone/risklimits;

weight weight;

run;


proc surveyphreg data=mydata.cohort_final_1_A;

class  white(ref='0') black(ref='0') hisp(ref='0')

      high_hrc(ref='0') stage_hrc(ref='0')

      diabetes(ref='0') hypertension(ref='0') wasting(ref='0')
malaise(ref='0') osteoporosis(ref='0') pituitary_dysf(ref='0')

      depression(ref='0') hyperlipidemia(ref='0') cardiovascular(ref='0')
insulin(ref='0') cci_cat(ref='0') drug_cat(ref='None')/param=ref;

strata match_id;

model timeD*oc_mortality(0)=age_exposed white black hisp diabetes high_hrc
stage_hrc

            hypertension wasting malaise osteoporosis pituitary_dysf

            depression hyperlipidemia cardiovascular insulin cci_cat
visits_no education poverty /*breast_test_no col_test_no*/ ov_test_no

            drug_cat/risklimits;

weight weight;
```

```
run;




**Among endometrial cancer only;

title "Among endometrial cancer only -outcome=HRC mortality";

proc freq data=mydata.cohort_final_1_A; table
endo_cancer_diag*cancer_mortality; run; /* OK */

data mydata.cohort_final_1_A;
set mydata.cohort_final_1_A;
if  endo_cancer_diag='1' and cancer_mortality=1 then ec_mortality=1;
***Endometrial cancer and death;
else ec_mortality=0;
run;
/* NOTE: The data set MYDATA.COHORT_FINAL_1 has 142776 observations and 122
variables. */

proc freq data=mydata.cohort_final_1_A; table ec_mortality; run; /* OK */


proc freq data=mydata.cohort_final_1_A; table (statin testosterone
drug_cat)*ec_mortality; weight weight; run; /* OK */


proc surveyphreg data=mydata.cohort_final_1_A;

class  white(ref='0') black(ref='0') hisp(ref='0')

      high_hrc(ref='0') stage_hrc(ref='0')

      diabetes(ref='0') hypertension(ref='0') wasting(ref='0')
malaise(ref='0') osteoporosis(ref='0') pituitary_dysf(ref='0')

      depression(ref='0') hyperlipidemia(ref='0') cardiovascular(ref='0')
insulin(ref='0') cci_cat(ref='0') statin(ref='0')/param=ref;

strata match_id;

model timeD*ec_mortality(0)=age_exposed white black hisp diabetes high_hrc
stage_hrc

            hypertension wasting malaise osteoporosis pituitary_dysf

            depression hyperlipidemia cardiovascular insulin cci_cat
visits_no education poverty /*breast_test_no col_test_no ov_test_no*/

            statin/risklimits;

weight weight;

run;
```

```
proc surveyphreg data=mydata.cohort_final_1_A;

class  white(ref='0') black(ref='0') hisp(ref='0')

      high_hrc(ref='0') stage_hrc(ref='0')

      diabetes(ref='0') hypertension(ref='0') wasting(ref='0')
malaise(ref='0') osteoporosis(ref='0') pituitary_dysf(ref='0')

      depression(ref='0') hyperlipidemia(ref='0') cardiovascular(ref='0')
insulin(ref='0') cci_cat(ref='0') testosterone(ref='0')/param=ref;

strata match_id;

model timeD*ec_mortality(0)=age_exposed white black hisp diabetes high_hrc
stage_hrc

            hypertension wasting malaise osteoporosis pituitary_dysf

            depression hyperlipidemia cardiovascular insulin cci_cat
visits_no education poverty /*breast_test_no col_test_no ov_test_no*/

            testosterone/risklimits;

weight weight;

run;


proc surveyphreg data=mydata.cohort_final_1_A;

class  white(ref='0') black(ref='0') hisp(ref='0')

      high_hrc(ref='0') stage_hrc(ref='0')

      diabetes(ref='0') hypertension(ref='0') wasting(ref='0')
malaise(ref='0') osteoporosis(ref='0') pituitary_dysf(ref='0')

      depression(ref='0') hyperlipidemia(ref='0') cardiovascular(ref='0')
insulin(ref='0') cci_cat(ref='0') drug_cat(ref='None')/param=ref;

strata match_id;

model timeD*ec_mortality(0)=age_exposed white black hisp diabetes high_hrc
stage_hrc

            hypertension wasting malaise osteoporosis pituitary_dysf

            depression hyperlipidemia cardiovascular insulin cci_cat
visits_no education poverty /*breast_test_no col_test_no ov_test_no*/

            drug_cat/risklimits;

weight weight;
```

```
run;

ods rtf close;

/* KM curves */

%ProvideSurvivalMacros

%let yOptions = label="Survival"
                linearopts=(viewmin=0.4 viewmax=1
                            tickvaluelist=(0 .2 .4 .6 .8 1.0));

%CompileSurvivalTemplates

ods graphics on;

proc lifetest data=mydata.cohort4 plots=s(test atrisk(atrisktickonly
outside)=0 365 730 1095 1460 1825 2190 2555 2920 3285 3650);

/*where drug_cat ne '0';*/

time time*death_new(0);

strata drug_cat/order=internal;

ods output survivalplot=sp;

run;

ods graphics off;

ods graphics on;

proc lifetest data=mydata.cohort4 plots=s(test atrisk(atrisktickonly
outside)=0 365 730 1095 1460 1825 2190 2555 2920 3285 3650);

where cancer_diagnosed='1';

time time*death_new(0);

strata drug_cat/order=internal;

ods output survivalplot=sp;

time time*death_new(0);

strata testosterone/order=internal;
```

```
ods output survivalplot=sp;

run;

ods graphics off;

th_new(0)=age_exposed white black hisp advanced_cancer_stage high_tumor_grade
diabetes hypertension wasting malaise osteoporosis pituitary_dysf

            depression hyperlipidemia cardiovascular insulin cci_cat
visits_no education poverty breast_test_no col_test_no ov_test_no

            drug_cat/risklimits;
run;


*Among non-cancers diagnosed patients only;
proc phreg data=mydata.cohort4;

where cancer_diagnosed='0';

class death(ref='0') white(ref='0') black(ref='0') hisp(ref='0')
/*advanced_cancer_stage(ref='0') high_tumor_grade(ref='0')*/

      diabetes(ref='0') hypertension(ref='0') wasting(ref='0')
malaise(ref='0') osteoporosis(ref='0') pituitary_dysf(ref='0')

      depression(ref='0') hyperlipidemia(ref='0') cardiovascular(ref='0')
insulin(ref='0') cci_cat(ref='0')  sta(ref='0')/param=ref;

strata match_id;

model time*death_new(0)=age_exposed white black hisp /*advanced_cancer_stage
high_tumor_grade*/ diabetes hypertension wasting malaise osteoporosis
pituitary_dysf

            depression hyperlipidemia cardiovascular insulin cci_cat
visits_no education poverty breast_test_no col_test_no ov_test_no

            sta/risklimits;

run;


/* Rates */

proc freq data=mydata.cohort4;
table (sta tes drug_cat)*death/chisq fisher;
run;

proc freq data=mydata.cohort4;
where bca='1';
table (sta tes drug_cat)*death/chisq fisher;
run;

proc freq data=mydata.cohort4;
where cca='1';
```

```
table (sta tes drug_cat)*death/chisq fisher;
run;

proc freq data=mydata.cohort4;
where oca='1';
table (sta tes drug_cat)*death/chisq fisher;
run;

proc freq data=mydata.cohort4;
where eca='1';
table (sta tes drug_cat)*death/chisq fisher;
run;


proc freq data=mydata.cohort4;
where cancer_diagnosed='1';
table (sta tes drug_cat)*death/chisq fisher;
run;

proc freq data=mydata.cohort4;
where cancer_diagnosed='0';
table (sta tes drug_cat)*death/chisq fisher;
run;
```
